# Automation-Assisted Photoinduced Atom Transfer Radical Polymerization

**DOI:** 10.1021/acspolymersau.5c00067

**Published:** 2025-08-28

**Authors:** Cesar Ramirez, Eman Ahmed, Elena Di Mare, Maria Pineiro-Goncalves, Apostolos Maroulis, Prajakatta Mulay, D. Christopher Radford, Adam J. Gormley

**Affiliations:** Department of Biomedical Engineering, Rutgers, The State University of New Jersey, Piscataway, New Jersey 08854, United States

**Keywords:** atom transfer radical polymerization, robotics, machine learning, high-throughput, combinatorial

## Abstract

Oxygen-tolerant reversible-deactivation radical polymerizations (RDRP) now allow many of these reactions to proceed in open labware, such as well plates. This enables the high-throughput synthesis of tailored polymers and lowers the knowledge barrier required to obtain these materials. Building on our previous work automating photoinduced electron/energy transfer reversible addition–fragmentation chain transfer (PET–RAFT) and enzyme-assisted RAFT (Enz-RAFT) polymerization, we now introduce automated atom transfer radical polymerization (ATRP). Here, we demonstrate the potential of this platform for the high-throughput optimization of ATRP chemistry. Furthermore, we demonstrate that this workflow can help provide insights into the selection of reaction components, such as ligands and initiators, for the polymerization of kinetically difficult monomers such as methyl methacrylate with smaller rates of propagation than acrylates. This coupling paves the way for data-driven optimization of ATRP reactions, accelerated by the generation of high-throughput data sets. To facilitate the integration of robotics for high-throughput applications in polymer synthesis and optimization of photo-ATRP, we have made a Python package available to assist with experimental planning. The tool accepts Excel sheets with user-defined molar ratios, target monomer concentrations, and reagent stock concentrations and outputs an Excel sheet with actionable recipes that can be readily implemented in liquid handling transfer steps via manual or automated pipetting.

## Graphical abstract

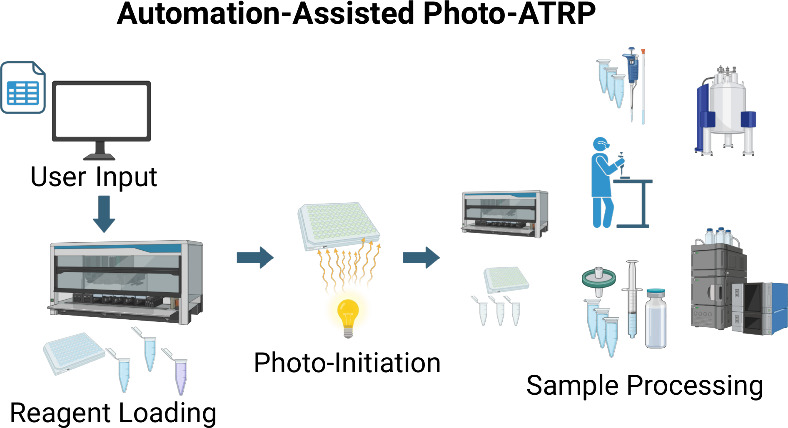

## 1 Introduction

Polymers are widely used for an extending range of applications in biology and medicine, including protein stabilization, drug delivery, and tissue engineering.^1–3^ Oxygen-tolerant reversible-deactivation radical polymerizations (RDRPs) such as reversible addition–fragmentation chain transfer (RAFT) and atom transfer radical polymerization (ATRP) have enabled more practical and scalable approaches in polymer synthesis.^4–10^ These also provide versatile tools to control architecture, composition, and molecular weight (MW) distributions from simple and open labware.^11,12^ The application of light as an energy source has also been a significant advancement in polymer science.^12–14^ Thus, techniques like photoinduced ATRP (photo-ATRP), photoinduced electron/energy transfer RAFT (PET-RAFT), and enzyme degassing of RAFT (Enz-RAFT) have allowed the synthesis of polymer materials under environmentally benign conditions.^4,8,12^

ATRP is a catalytic process that utilizes a metal (often copper, Cu), and a ligand (L) to form a metal–ligand complex, where the transition metal can exist in two oxidation states; an alkyl halide as an initiator; and, depending on the chemistry, a photocatalyst.^15–19^ Polymers obtained through ATRP have an alkyl group at the α-chain end and a halide moiety at the ω-chain end from the alkyl halide initiator.^19^ In ATRP, a halogen transfers from a dormant C­(sp^3^)–X polymer chain end to the [Cu^I^/L]^+^ activator, forming the [X–Cu^II^/L]^+^ deactivator (Figure S1). New techniques use activator regeneration for oxygen tolerance, as this catalytic system acts as an oxygen scavenger.^12,18–20^ Photo-ATRP and PET-RAFT are both versatile techniques, allowing the synthesis of polymers from various monomer families while providing well-defined control over polymerizations.^18^ Thus, knowledge of both techniques provides unprecedented versatility in polymer research, especially for high-throughput applications.^20,21^

The versatility of ATRP chemistry also creates a challenge when defining reagent conditions (i.e., ligand, alkyl halide initiator, etc.) where optimization is required.^14,17,22^ For instance, solvent viscosity and polarity impact conversion, oxygen dissolution, activator concentration, stability of the initiator, and polymerization rate.^14,19^ Typically, reaction optimization is iterative and low-throughput, necessitating systematic approaches to accelerate learning.^12,15^ Developments in high-throughput experimentation and machine learning (ML) offer the potential to accelerate reaction optimizations. ML can provide structure–function insights due to its ability to model high dimensional problems within a design space.^23–25^ The advent of approachable ML toolkits and user’s guides can lower the barrier of entry for new users of these tools,^26^ whereby data-driven material discovery incorporating automation embodies a new frontier in material research.^27^ However, ML modeling of physical experiments is often limited by the need for large data sets.^23^ This highlights the value of high-throughput and automated experiments to effectively exploit ML for reaction optimization.

The application of automation in polymer synthesis is an ongoing area of interest.^28^ For instance, chemistries and techniques suitable for well plates and high-throughput workflows continue to be developed.^12,18^ High-throughput methods have been previously used to facilitate the optimization of reaction conditions for activator regenerated by electron transfer (ARGET) ATRP, which inspired this work of automation-assisted photo-ATRP.^15^ This utilized a liquid handling robot to dispense into sealed vials under stirring, showing significant improvement in lowering labor burden and thus accelerating reaction optimization.^15^ PET-RAFT and Enz-RAFT were previously automated using a Hamilton liquid handling robot.^4,29^ Advantages of automated chemistry include high reproducibility and increased efficiency by lowering the need for manual labor and specialized knowledge. Automated PET-RAFT has been previously used to accelerate the discovery of polymer–protein hybrids using methacrylate polymers.^30^ This automation was pivotal to accelerate the synthesis of tailored polymers discovered through active learning and their characterization in high-throughput.^24,30^ Using liquid handling robots for polymer synthesis can simplify complex chemistries, reducing both the labor and time required for tasks such as reagent optimization. For example, liquid handling robots have been utilized for high-throughput polymer synthesis of methacrylates.^4,30,31^ To the best of our knowledge, this is the first reported automation of oxygen‑tolerant photo‑ATRP for polymer synthesis and optimization. This work thus represents a new integration of these technologies and paves the way for more streamlined, automated approaches to polymer research. Automated polymer synthesis continues to expand, where automation has been used to develop advanced polymer materials including multiblock copolymers and for the synthesis of polymer libraries, many utilizing RAFT.^32–36^

This work introduces an automation-assisted workflow for oxygen-tolerant photo-ATRP to streamline polymer synthesis, generate data in high throughput, and optimize reactions with ML. Building on the group’s previous work automating PET-RAFT and Enz-RAFT, we now enhance our automated polymer synthesis platform by integrating photo-ATRP.^4,29^ This platform allows the translation of chemistries from the literature to high-throughput formats and the screening of reaction conditions to adapt these in a different setting than their development. Many of these chemistries, as originally developed, are not inherently compatible with high-throughput platforms. For example, they could depend on high final monomer concentration ([M]_f_) resulting in viscous products that are difficult to aspirate or convert in vials, or they may rely on specialized light sources not suited for well-plate systems. We demonstrate this by first adapting chemistry from the literature to be suited for high-throughput synthesis in well plates for acrylates, as proof of concept.^37^ A visual overview of this platform is shown in Figure 1a, which highlights its four core components: automated planning of reaction recipes from reagent ratios, high-throughput liquid handling, ML-based initiation prediction, and combinatorial reagent screening. These elements work in concert to streamline optimization efforts and reduce manual labor. Although the ML implementation was performed retrospectively, it simulates a real-world scenario where early data collected using the platform could train models to predict which reagent combinations are likely to result in successful polymerizations. Lastly, we provide a pip-installable package, polymerization-planner, to facilitate experimental design by enabling the rapid generation of reagent recipes for polymerization screening.

**Figure 1:**
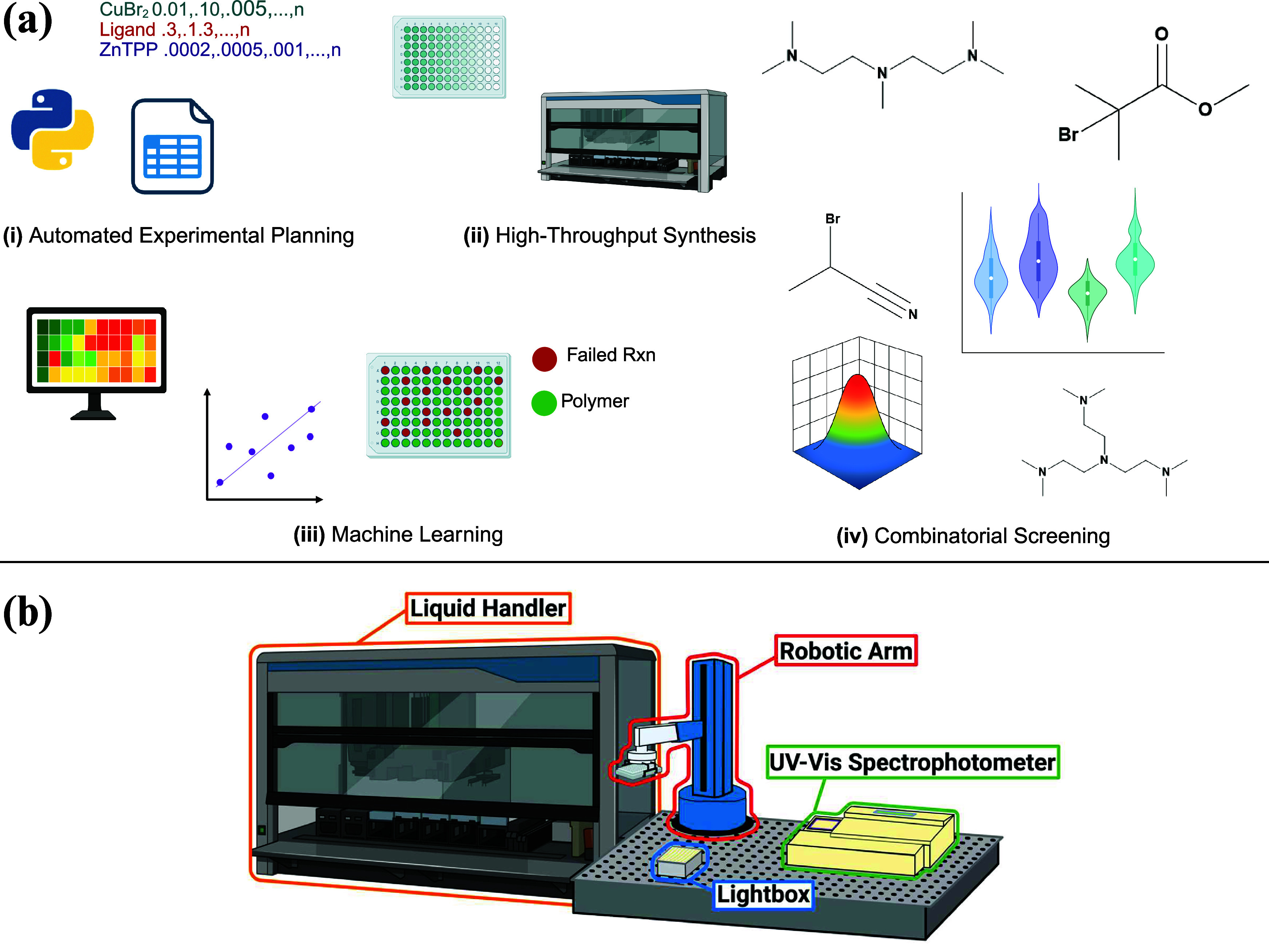
(a) Overview of the automation-assisted workflow for high-throughput oxygen-tolerant photo-ATRP optimization. (i) Automated planning generates reagent recipes by screening combinations of ligand, metal catalyst, and photocatalyst ratios. (ii) A Hamilton liquid handling robot dispenses these recipes into well plates for high-throughput polymerizations. (iii) ML models trained on experimental data predict conditions likely to initiate polymerization, reducing trial-and-error. (iv) Combinatorial screening of ligands and initiators reveals their influence on polymerization outcomes. (b) Components of automated synthesis platform used in this work, which include a Hamilton liquid handling robot, a custom-made light box with 560 nm LEDs (5 mW cm^–2^), a robotic arm, and an UV–vis spectrometer. In this application, only the liquid handler and the light box were utilized. (a) Created with Biorender. (b) Adapted with permission under a Creative Commons CC BY 3.0 license from ref 29. Copyright 2023 The Royal Society of Chemistry.

## 2 Results and Discussion

### 2.1 Automation of ATRP

As a proof of concept, our first objective was to adapt ATRP chemistry from the literature that was developed using red light to the high-throughput polymer synthesis platform in our laboratory (Figure 1b).^37^ Here, the ability to perform controlled polymerization served as a metric for success. This platform includes a Hamilton Microlab STARlet liquid handling robot and a light box with 560 nm LEDs (5 mW cm^–2^).^29^ The components of the platform are controlled by custom Python software, referred to as PolyCraft. This software serves as an interface between the liquid handler and user inputs provided in the form of an Excel sheet. The influence of reagent molar ratios (metal, ligand, and photocatalyst) on the resulting size-exclusion chromatography (SEC) trace was investigated. For this, a custom experiment was designed where the final concentrations of each reagent were varied while keeping the remaining reagent concentrations fixed (Figure S2). During this investigation, the monomer final concentrations were 1 M as this is a standardized final monomer concentration typically used in our high-throughput polymer synthesis platform. The standard practice includes carrying out reactions at 200 μL volumes, thus leaving 100 μL of oxygen headspace. In a custom experiment, the user explicitly designs each transfer step (e.g., the volume of each reagent, reagent pickup and drop-off location). In contrast, an automated experiment requires only that the user input the desired reagent molar ratios and the available stock concentrations. Based on this information, the full sequence of robotic transfer steps needed to carry out the experiment is autonomously generated by a script without requiring the user to manually define each movement or volume.

Linear homopolymers of 2-hydroxyethyl acrylate (HEA) with a degree of polymerization (DP) of 200 were synthesized robotically in 96-well plates. This was done using methyl-α-bromoisobutyrate (MBiB) as the initiator, tris­(2-dimethylamino ethyl)­amine (Me_6_TREN) as the ligand, zinc­(II) tetraphenyl porphine (ZnTPP) as the photocatalyst (PC), and CuBr_2_ as the metal catalyst. Reactions were carried out in dimethyl sulfoxide (DMSO) with 100 μL of headspace, and polymerizations were characterized by using SEC and nuclear magnetic resonance (NMR) spectroscopy. The adapted chemistry used the following reagent ratios: HEA/MBiB/CuBr_2_/Me_6_TREN/ZnTPP = 200/1/0.08/0.2/0.002. Once the chemistry performance was confirmed using the standardized laboratory synthesis procedures, different final monomer concentrations ([M]_f_) were also investigated (Figure S3).

Good MW control was observed across a range of targeted DP (30–200) while maintaining low dispersity (*Đ* < 1.3) (Figure 2a). Results show that MW increases linearly with the DP while dispersity remains low, confirming that our automation-assisted ATRP retains MW control and is amenable to automated procedures. Living character provided by the active halide chain ends of the synthesized polymers was confirmed via chain extension experiments, where a pHEA_50_ macroinitiator was successfully extended with methyl acrylate (MA), resulting in well-defined block copolymers with a final *Đ* of 1.28 (Figure 2b).^14^ Lastly, the ability to synthesize different acrylate polymers and statistical copolymers was investigated (Table 1). These results demonstrate that oxygen-tolerant ATRP can be adapted for high-throughput and automated polymer synthesis in 96-well plates.

**Figure 2:**
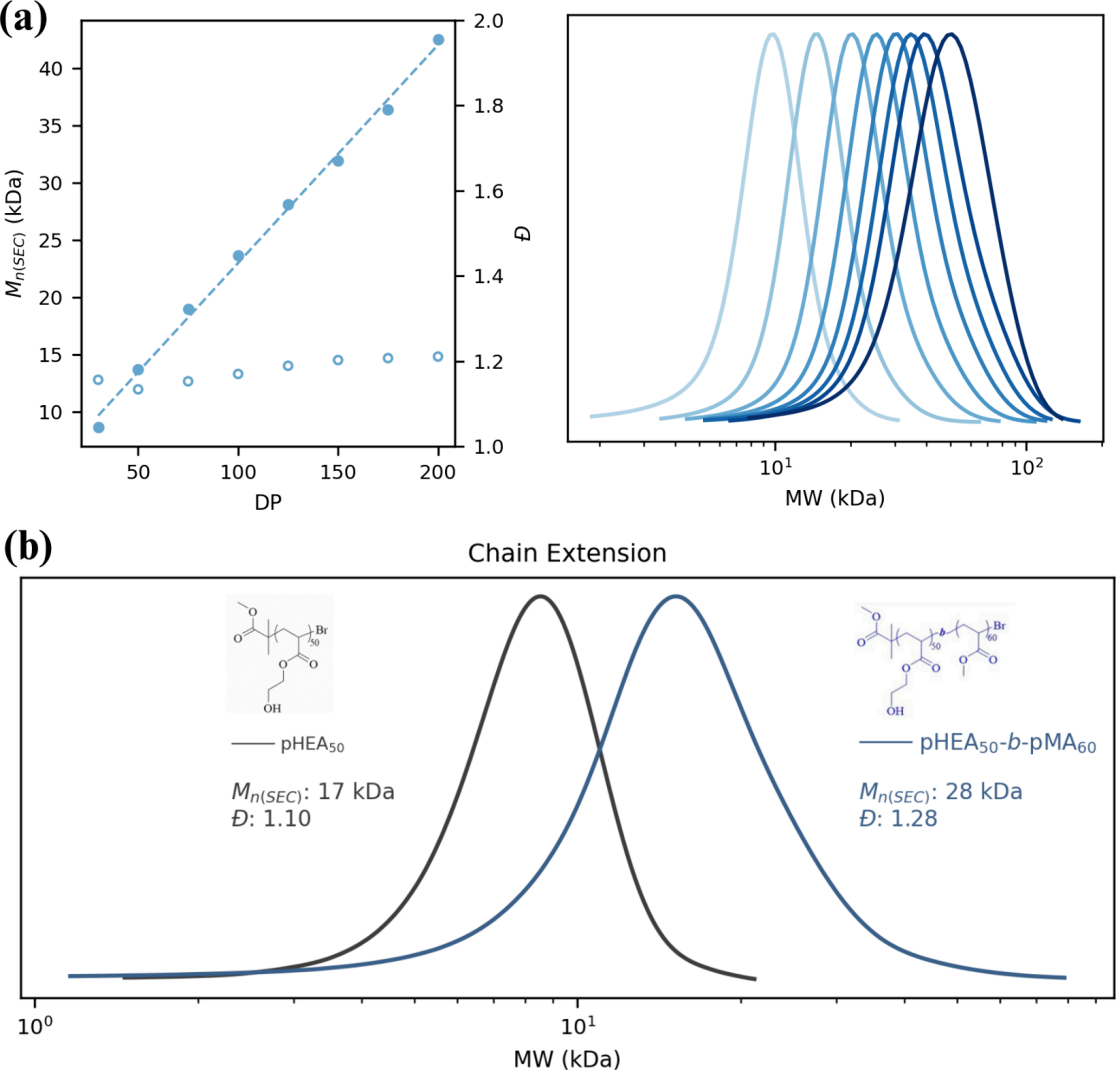
(a) Automation-assisted photo-ATRP of acrylates in well plates showing good control over the MW and narrow *Đ*. DP varied from 30 to 200, final monomer concentration was 2 M, and polymerization proceeded for 5 h. (b) Confirming living character through chain extension of pHEA with a MA by sequential monomer addition.

**Table 1:** Acrylate Polymerizations Prepared by Automated Photo-ATRP in 96-Well Plates

Polymer	DP	*M*_n,th_ (kDa)	*M*_n(SEC)_ (kDa)	*Đ*	Conversion (%)
pHEA	200	23.4	53.2	1.15	95
pMA	200	17.4	17.8	1.12	79
pHPA	200	26.2	52.8	1.16	93
p(HEA-*st*-MA-*st*-HPA)	200	22.3	35.0	1.12	91

### 2.2 High-Throughput Reaction Optimization

Photo-ATRP reaction variables that influence polymerization outcomes include the metal catalyst, PC, ligand, solvent, temperature, and light intensity and wavelength.^13,17,20,22^ Therefore, the primary objective of developing a Python script for autonomous reagent handling is to enable rapid systematic exploration of these conditions with high throughput. This is especially relevant to ATRP as small variations in reaction conditions can easily influence polymerization reactions.^14,20^ For instance, the induction period can be influenced by factors including the head space volume (oxygen concentration) as well as light intensity and wavelength.^13^ The optimization of reagent molar ratios is critical to overcome these limitations and ensure reproducibility and controlled MW distributions.^14,15^ ATRP literature was surveyed to identify common practices in reaction optimization and select various reagent molar ratio combinations to test script robustness and identify limitations.^13,15–18,38^ The selected ratios are shown in Table S1, representing a total of 270 possible reagent molar ratios to test. HEA was used as a model monomer with a target DP of 200 and a final monomer concentration of 1 M. From the 270 possible ratio combinations, 167 were synthesized and characterized through SEC using a high-throughput column, with NMR performed on a portion of these. Out of the 270, the script generated reaction handling steps for all test ratios, demonstrating robustness at handling multiple reagent ratios for optimization campaigns. This script is not limited to ATRP reactions, as the concepts employed here can easily be adapted for optimization of PET-RAFT reactions as well.

A diagram behind the script inputs and logic employed to determine whether a user-specified reaction is possible is shown in Figure S4. Inputs include molar ratios, the final monomer concentration, and the concentration of the stock reagents, where high stock concentrations are preferred, as this allows more flexibility for dilutions to prepare autonomously. Tables S2, S3 show sample user-input Excel sheets for optimization using a single monomer. In the context of this work, only the ratios of metal, ligand, and PC were targets for evaluating ability to carry out optimization synthesis. Table S3 shows the sheet defining the monomer and copolymer compositions for up to four different monomers. This accelerates optimization campaigns by automating reagent transfers into well plates and, when necessary, the preparation of dilutions of stock reagents to meet volume and concentration requirements for each reagent being optimized. The choice of additional dilutions of a stock reagent is made based on the concentrations that apply to multiple test ratios within the experiment to minimize unnecessary reagent preparation. The user may indicate alternative dilutions that the script may consider and prepare if necessary. For example, the user can provide a ligand stock solution at 100 mM and inform the script that this can make dilutions at 50, 25, and 11.5 mM if needed. The script can carry out experiments with different initiators based on their pickup location on the robot and their dispense location on the well plate.

A limitation in applying liquid handling robots to high-throughput experimentation often lies in requiring user expertise in programming languages to generate reagent handling steps that can be interpreted by the liquid handling instrument. This limitation can be partially overcome through the adaption of current open-source tools to facilitate interfacing between liquid handling instrumentation such as pyHamilton.^39^ Recently, PyLabRobot was introduced which is an open-source, cross-platform Python interface for liquid handling robots including Hamilton STARs and Vantages, Tecan EVOs, and Opentron OT-2s, further enhancing accessibility to newcomers in automated works.^40^ The automation-assisted ATRP workflow shown here can be applied to different interfacing software following modification of the volume transfers needed from the output DataFrame to the platform utilized by the user. Code developed for reaction planning is publicly available to facilitate adaptation in diverse research settings. The package can be installed via pip install polymerization-planner, either directly from a terminal or within a Google Colab notebook using !pip install polymerization-planner followed by from polymerization_planner import atrp_planner.

Efficiency in optimization experiments was demonstrated by carrying out a mock experiment using water and food coloring, where 24 molar ratios were synthesized manually and using the automation described. The results show significant efficiency gains of when automation is used (Figure 3) compared to manual preparation by an experienced lab member being three times slower. For the stock preparation step, the robot was one min slower than a human. However, the concentrations of different stocks to prepare were informed by the script; thus, no experimental planning was considered (Tables S4, S5). The inclusion of experimental planning would have resulted in increased time required prior to starting the experiment. Furthermore, during manual preparation, three “polymer” samples needed to be remade as the experimenter made three mistakes, of which they were aware. This approach demonstrates significant advancements in the automation of RDRP and reaction optimization processes. Particularly for photo-ATRP, where reagent preparation and reagent transfers are automatically handled, there is an increase in efficiency in experimental procedures.

**Figure 3:**
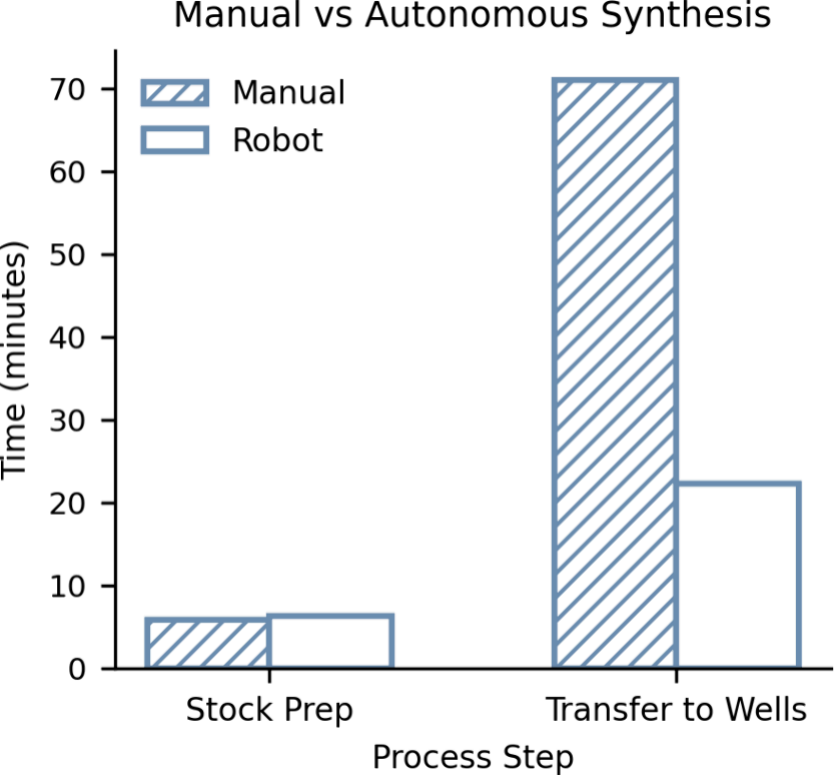
Comparison of time required to prepare stocks and transfers into wells for an optimization experiment of 24 test molar ratios. During manual transfers to wells, three mistakes were made by humans, requiring these to be prepared again.

The reagent recipes generated by the script can be translated into dispensing protocols for liquid handling robots or used to assist in the planning of manual experiments. This package includes functions to plan experiments for both photo-ATRP and PET-RAFT (CTA and PC). We believe that this tool, which converts molar ratios into useful volumes based on available reagent stock concentrations, can increase accessibility and efficiency by facilitating experimental planning, especially for newcomers. The inputs to the planning functions within the Python package consist of reagent molar ratios, the final monomer concentration and volume, and a list of optimizable reagent concentrations available. A straightforward approach is to make a high concentration stock of each optimizable reagent (CTA and PC for PET-RAFT and metal, ligand, and PC for ATRP) and list concentrations that can be prepared by serial dilution. The code will then iterate over these and choose the concentrations that can meet a given reagent molar ratio as well as minimize the number of additional dilutions to prepare. To the best of our knowledge, there are no other public tools to automate the translation of reagent ratios to experimental recipes. Online calculators for dilutions or molar conversions exist, but custom in-lab spreadsheets for ATRP and RAFT reactions are not standardized or publicly available, especially for batch experimental planning. Thus, we hope this tool can support polymer research by alleviating time-consuming calculations and experimental protocol design, lowering the knowledge barrier for newcomers to the technique. It is available via a pip install to serve both the automation and polymer communities. A link to the tutorial can be easily accessed after installation by running from polymerization_planner import show_tutorial followed by show_tutorial­().

### 2.3 Applying Machine Learning (ML) to Predict Reaction Polymerization

Identifying the correct conditions for initiating an ATRP reaction is essential. Only once these conditions are identified is it possible to optimize other aspects of the reaction such as MW, *Đ*, conversion, or end-group fidelity. This challenge is particularly pronounced when considering the adaptation of oxygen-tolerant procedures to high-throughput, as alterations in conditions can disturb the mechanisms providing oxygen-tolerance,^20^ especially considering that when liquid-handling robotics are used, practical constraints arise that might require modification of an experimental protocol to ensure suitability. For example, reactions with very high [M]_f_ can produce viscous or gel-like products that are difficult to aspirate or dispense accurately. Unlike a human, a robot may not detect issues such as partial dispensing or tip occlusion, limiting the feasibility of such conditions in high-throughput formats. Using the data collected during the development of the platform, we carried out a retrospective simulation to explore whether ML could identify suitable conditions by predicting whether polymerization would occur, in this context referred to as “initiation”. Here, we classified the occurrence of initiation based on observable signals from NMR (Figure S5) and SEC (Figure S6) that indicated polymerization of HEA monomers. In NMR, successful initiation would yield a signal appearing in the upfield region of the spectra pertaining to the repeating unit of pHEA and peak shifts from protons in the monomer versus polymer.^38^ In SEC, a flat trace indicated no initiation, as no polymer product was observed. In the script development phase, batches having different numbers of combinations of ligand, metal catalyst, and PC were tested. Only the molar ratios of Me_6_TREN, CuBr_2_, and ZnTPP with respect to the initiator were varied between samples (Table S1), with monomer concentration fixed at 1 M and 10 h of light radiation. Some samples that showed no signs of polymerization after 10 h could have a longer induction period and eventually initiate.^13,17,22^ However, our objective was to evaluate if ML could identify conditions that would result in polymerization within 10 h.

We trained binary classification models to simulate real-world sequential optimization by treating each batch of data as if this represented a different stage in the optimization campaign. The first batch was used to train a model to predict outcomes in the second batch. The model was then retrained with the combined data from both batches to predict the outcomes in the third batch. Stochastic gradient descent (SGD) classifiers were trained using the molar ratios of Me_6_TREN, CuBr_2_, and ZnTPP as input features, following standard ML practices for data preprocessing and model evaluation (see section 4.7).^26^ Balanced accuracy was given a preference for model evaluation to account for the inherent class imbalance often present during reaction screening (Table S6). Data from the first batch was used to train a model to predict the unseen data from the second batch resulting in a balanced accuracy score of 88% (Figure 4a, Table S7). This was followed by combining the data from the first two batches and retraining the model to predict the initiation of the samples in the third batch where the model correctly predicted polymerization occurrence for all samples (Figure 4a, Table S8). The results from this simulation suggest how ML can be a tool to facilitate reaction optimization by accurately predicting reagent molar ratio combinations that will likely result in polymerization. While an experienced ATRP chemist may anticipate these trends, the ability to train models from data makes this approach accessible to nonexperts and suitable for autonomous optimization. This framework can also be extended to predict other properties such as *Đ* or MW, paving the way for broader ML-driven exploration in ATRP, similar to what has been demonstrated in RAFT systems.^28,41^ Therefore, these findings showcase how ML can be strategically integrated with automated platforms to improve the experimental efficiency. In our case, after training on the first two batches, the model achieved perfect accuracy. The batches used here were selected arbitrarily to validate the script rather than to maximize model learning. Experimenters could begin optimization campaigns testing small numbers of reagent ratio combinations, that ideally have been chosen via efficient sampling techniques such as Latin hypercube sampling to explore the design space, and ML could distinguish viable from nonviable polymerization conditions. Once the model exhibits a high predictive performance, subsequent larger batches can be synthesized with greater confidence that the selected conditions will result in a polymer that can be characterized. This in turn will result in a reduction in material waste and failed reactions. Meanwhile, maximizing the likelihood that each experiment yields informative data is particularly relevant when optimizing properties like *Đ*.

**Figure 4:**
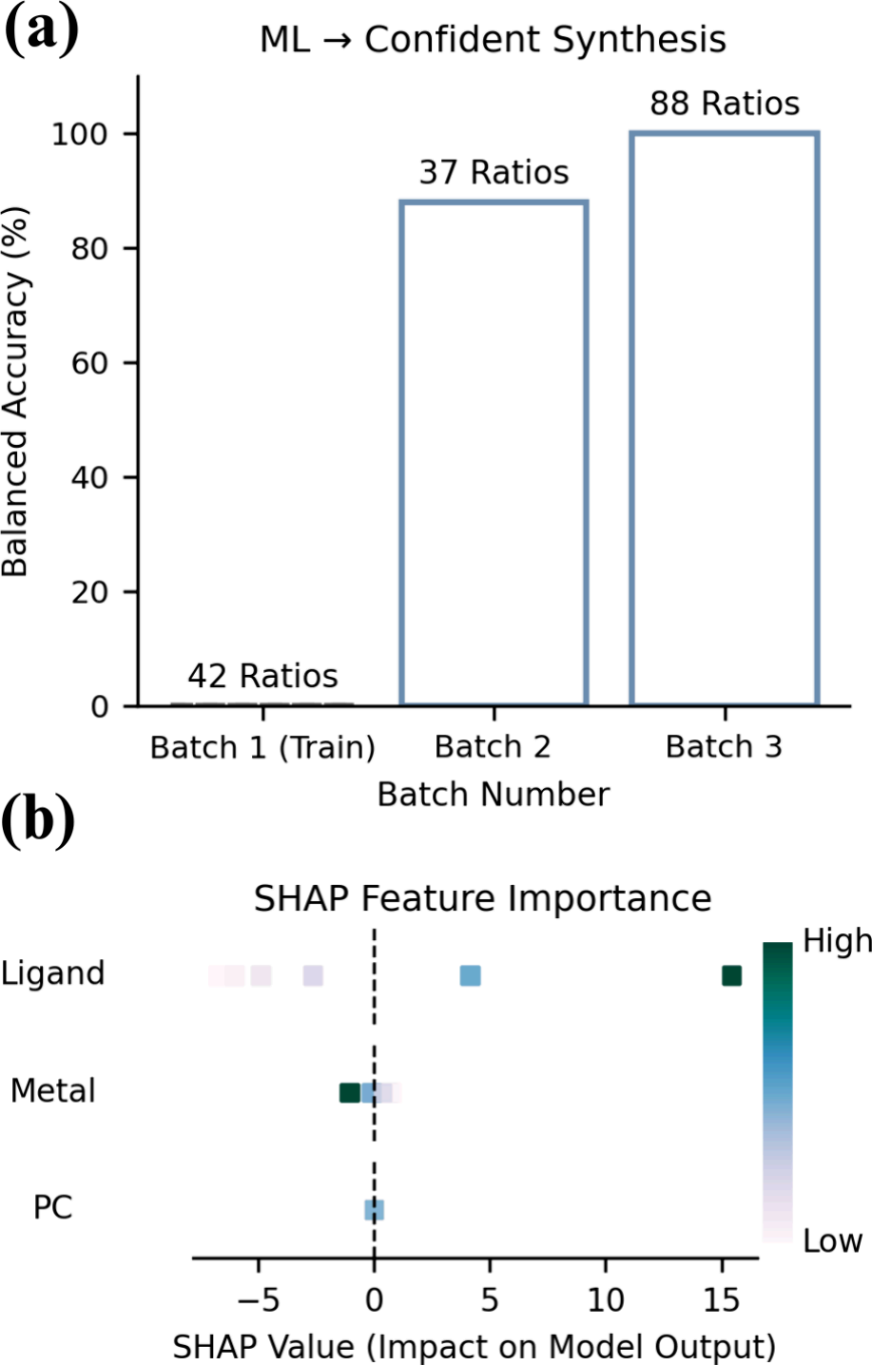
(a) Model accuracy improves across sequential batches of different reagent ratios, enabling a confident prediction of initiation outcomes after just two small-scale experiments. This facilitates broader exploration of molar ratios with fewer failed reactions, critical when optimizing properties like MW, where each successful polymerization yields informative data. (b) SHAP analysis of the classifier trained on the first two batches to predict outcomes in the third batch. Higher ligand concentrations positively influenced model predictions, while higher metal concentrations had a negative effect, consistent with known ATRP chemistry in which the metal–ligand complex drives activation–deactivation balance.

Beyond predictive performance, understanding which features drive the model’s decisions can reveal mechanistic trends and guide further optimization efforts. Shapely Additive Explanations (SHAP) is a useful tool for understanding how each feature influences model predictions, assigning values to individual features by applying concepts from game theory.^26,42,43^ In this context, this informs how the molar ratio of the PC, metal, and ligand influences the probability that a sample will show a trace on the SEC and a signal in the NMR spectra indicating polymerization.^38^ SHAP analysis applied to the model trained on the combined data from the first two batches is shown in Figure 4b, where SHAP indicates that the ligand concentration has the greatest influence in the model predictions, followed by the metal catalyst, with the PC having the least influence. This is not surprising given that the metal–ligand complex drives the reaction as this alternates oxidation states to serve as the activator and deactivator.^13,15–20^ This is generally seen in ATRP where the concentration of ligand and metal can significantly affect conversion and the MW distribution.^14,20^ In regenerative ATRP, the polymerization rate is proportional to the square root of the ratio of Cu^I^ regeneration to the termination rate coefficient, while temporal control depends on ligand activity.^20^ The importance of the ligand and metal in ATRP was also evident in early stage experiments, where we independently varied the concentration of each reagent while keeping the others fixed. Adjusting the ligand or metal catalyst by a factor of 2 led to clear shifts in the SEC trace, whereas even a 4-fold change in the PC concentration had comparatively little effect (Figure S2). This analysis demonstrates the potential of ML beyond predictions but as a tool to reveal mechanistic insights. In addition to lowering the need for inefficient and extensive experimentation, ML can lower the barrier to entry for researchers adopting ATRP by elucidating key reaction components that influence the properties of a reaction.

In the paradigm of the self-driven lab, robotics, artificial intelligence, and automated data processing are integrated, resulting in autonomous data-driven experimental campaigns (Figure 5).^27,44^ The development of tools like inline NMR and online SEC further pave the way and are being integrated for the development of autonomous closed-loop experimentation.^41^ Knox and co-workers used a closed-loop system for optimization of RAFT polymerizations.^28^ Here, classification was used to demonstrate the potential of ML in predicting reaction “initiation” (SEC trace or NMR polymer signal) from an unbalanced data set. This was meant to pose a realistic example of polymerization optimization data sets as there will often be more instances of initiation or some polymerization, but polymer *Đ*, MW, or conversion might require further optimization. With the ability to generate data sets in high-throughput, ML approaches like the application of Gaussian process regression (GPR) might be utilized to further assist experimenters in optimization campaigns.^26,30^ GPRs are probabilistic ML models which provide uncertainty in their predictions.^25,45^ The uncertainty from GPRs can be applied to navigate the chemical space, dictate polymer properties, and efficiently plan experiments that minimize experimentation and thus accelerate optimization campaigns.^25,45^

**Figure 5:**
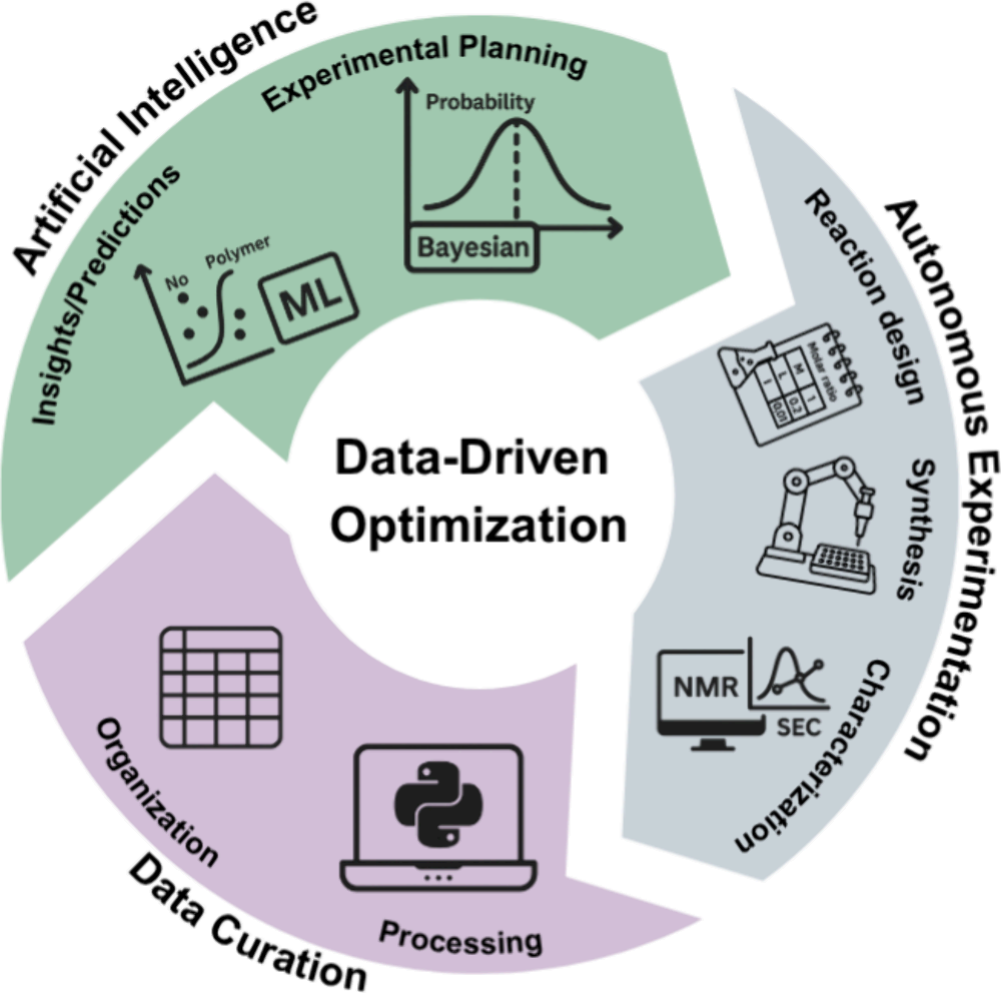
Schematic representation of a modular, data-driven polymer optimization framework. The process begins with reaction design, automated synthesis, and characterization (e.g., SEC and NMR). Extracted data is then processed and organized before undergoing ML analysis and Bayesian-based experimental planning. This iterative cycle enables efficient experimental design and supports the development of semi-autonomous or fully autonomous workflows. The platform presented here can support this future vision by facilitating experimental planning. Created using Canva.

### 2.4 Validating the Platform’s Ability to Perform Different Photo-ATRP Chemistries

The automation-assisted platform was validated by applying it to different reaction conditions and a new monomer class. The purpose of these validation experiments was to highlight the ability of the platform to facilitate the translation of chemistries into a high-throughput format and the exploration of new polymerization conditions. The first chemistry employed for validation was an open-air photo-ATRP system with Eosin Y (EYH_2_) as the PC, MBiB as the initiator, CuBr_2_ as the metal catalyst, Me_6_TREN as the ligand, and DMSO as the solvent developed by Szczepaniak and co-workers.^18^ While this chemistry had been previously reported for open-air polymerization of methyl acrylate (MA) in organic solvent within vial-based formats, it had not yet been adapted for high-throughput synthesis. Building on this foundational work, we translated the chemistry into a well-plate format compatible with automated liquid handling, enabling scalable experimentation. To better accommodate the high-throughput plate format used in this work,a less volatile monomer was selected. HEA was chosen for its lower vapor pressure and higher boiling point compared to MA, improving compatibility with the open setup.^46^

This adaptation process was carried out in three rational, iterative rounds to transition an open-air photo-ATRP system into a format compatible with high-throughput, well-plate-based synthesis, highlighting how automation alone even in the absence of ML can facilitate rapid and accessible optimization, empowering nonexperts and newcomers to attempt complex chemistries, such as ATRP.^15^ In the first round, literature conditions were applied directly using a 5.5 M monomer concentration to evaluate the compatibility. This was followed by SEC characterization after 1 and 5 h of light radiation with a green LED light box (2.61 mW/cm^2^, optical power characterized at 515 nm; Figure S7). The products from this first round produced highly viscous polymers that were difficult to handle using liquid handling robotics, prompting the need to reduce the monomer concentration. High-viscosity samples can pose a challenge for automated workflows due to the inability to properly aspirate and dispense these, which in turn leads to inaccurate reagent transfers. DP and polymer concentration influence viscosity in solution polymerization, so these parameters are potential avenues to address this challenge. Here, we aimed to maintain the same DP reported for this chemistry.^18^

In the second round, we tested both reagent ratios reported in the literature and new variations at [M]_f_ of 4.5, 3.5, and 2.5 M.^18^ In this step we aimed to identify a lower [M]_f_ to continue adaptation under conditions that would yield decreased polymer viscosity following 5 h of light radiation. We tested seven reagent molar ratios, where three ratios resulted in polymerization at 2.5 M, which is the lowest [M]_f_ tested. These were characterized by SEC having *Đ* ranging from 1.62 to 2.54 and *M*_n,SEC_ ranging from 77.1 to 100.5 kDa (Figure 6b, Table S9). The *M*_n,(SEC)_ values of most samples at these lower [M]_f_ were above the target range of 45–55 kDa, which we hypothesized was likely due to the quenching of initiator radicals by oxygen.^17,22^ This target range was chosen based on the proximity of *M*_n(SEC)_ obtained from synthesis of pHEA_200_ using the previously adapted oxygen-tolerant chemistry as detailed in section 2.1. Oxygen can be detrimental to ATRP systems as this oxidizes the lower valent copper catalyst, which limits initiator activation and propagation and causes termination.^14^ In the third round, [M]_f_ was fixed at 2.5 M, and metal, ligand, and initiator ratios were varied to fine-tune *M*_n(SEC)_ and *Đ* and to better understand their effects. The molar ratio chosen for fixed ligand or metal conditions was guided by literature, prioritizing ratios reported in the original study describing this system.^18^ We also tested a 2-fold increase in initiator concentration, based on our hypothesis that the previous increase in *M*_n,(SEC)_ observed in the second adaptation round was due to radical quenching by oxygen.^17^ Since the alkyl halide concentration is directly related to MW, increasing it was expected to lower *M*_n(SEC)_ near the target range.^14^ The increase in alkyl halide loading led to improvements in both the *M*_n(SEC)_ and *Đ*.

**Figure 6:**
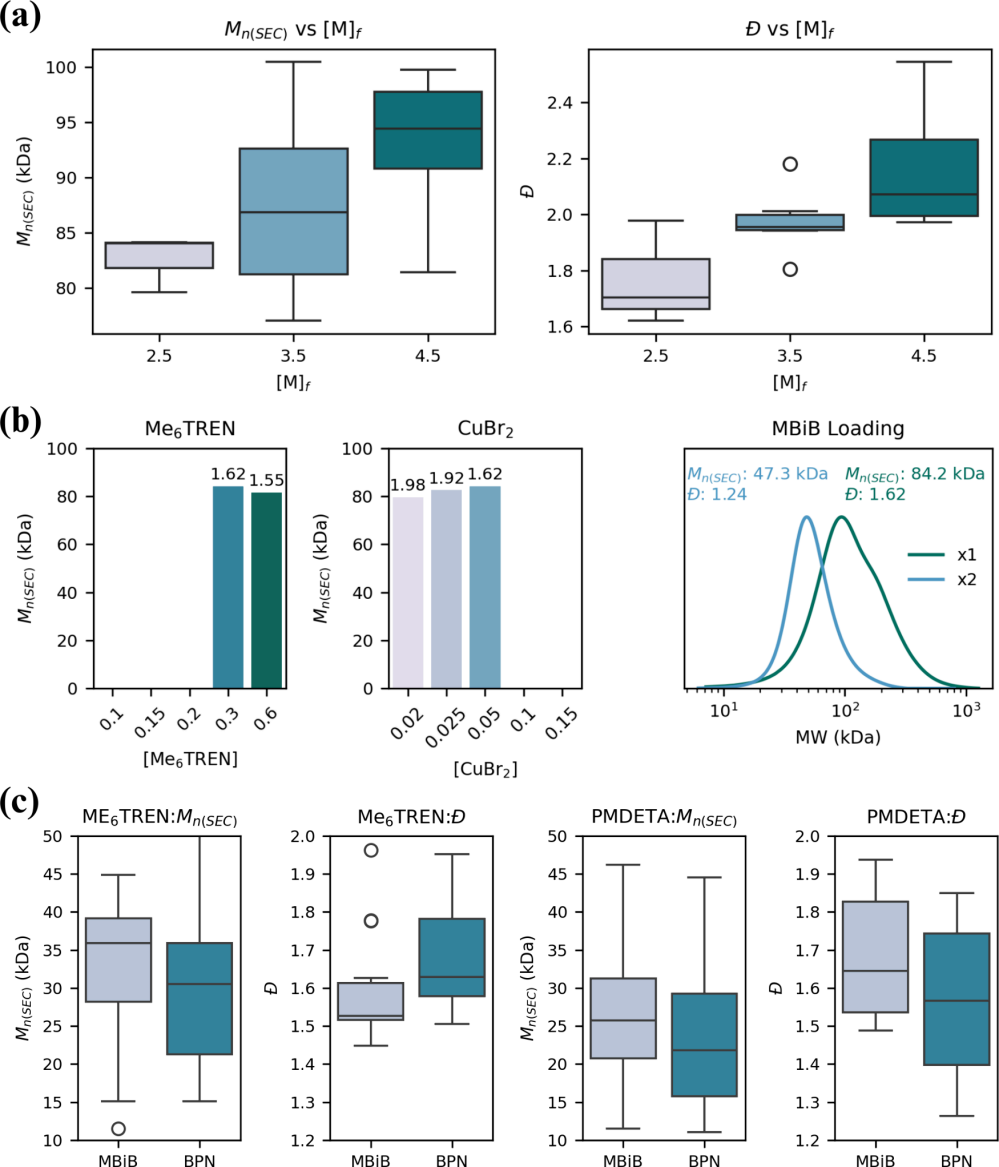
(a) Effect of [M]_f_ on *M*_n(SEC)_ and *Đ* across seven molar ratios to identify a lower [M]_f_ for viscosity reduction. Three of seven ratios resulted in polymerization at 2.5 M. (b) Effect of Me_6_TREN (left) and CuBr_2_ (middle) ratios on *M*_n(SEC)_, with *Đ* values indicated above each bar. EYH_2_ was fixed at 0.005, with either Me_6_TREN fixed at 0.3 or CuBr_2_ fixed at 0.05. Polymerizations only succeeded at or above 0.3 equiv of Me_6_TREN and below 0.08 equiv of CuBr_2_. (Right) SEC of optimized condition (2× initiator) at 2.5 M [M]_f_ and the unadjusted condition. (c) Distributions of *M*_n(SEC)_ and *Đ* for MMA polymerization using Me_6_TREN or PMDETA. Each box plot summarizes 32 polymerizations (2 initiators × 16 reagent ratios).

In the last adaptation round, *Đ* was reduced to 1.24, with *M*_n(SEC)_ reaching 47.3 kDa, within our target range (Table S10, Figure 6b). Notably, doubling the initiator under the conditions used in the initial compatibility evaluation while maintaining [M]_f_ at 2.5 M led to a marked improvement in *Đ*, with *M*_n(SEC)_ falling within the target range. In contrast, using [M]_f_ = 5.5 M yielded polymers with higher *Đ* and viscosity, despite achieving *M*_n(SEC)_ near the target. When [M]_f_ was held at 2.5 M but the initiator was not adjusted, *M*_n(SEC)_ deviated substantially from the target concentration (Figure 6b). These findings demonstrate how slight changes in conditions can dramatically affect synthesis outcomes, reinforcing the importance of high-throughput experimentation for adapting chemistries to new formats. The results from this adaptation underscore the value of automation in accelerating progress and its potential for broader application, especially for challenging chemistries like ATRP. The high-throughput nature of automation-assisted polymer synthesis lowers the barrier to exploring complex polymerizations and lays the groundwork for further improvements using machine learning-guided experimentation.

Polymerization of different monomer classes is challenging as the suitability of a catalytic system may vary based on monomer class.^14,47^ Prior findings have shown catalytic systems that are effective for polymerization of acrylates are not suitable for methyl methacrylate (MMA), where often ligands such as tris­(2-pyridylmethyl)­amine (TPMA) and *N*,*N*,*N*′,*N*″,*N*″-pentamethyldiethylenetriamine (PMDETA) are favored over Me_6_TREN.^14,16,22,47^ The ligand can have significant influence in a polymerization reaction; Me_6_TREN has been previously recognized as an inefficient deactivator for photo-ATRP of methacrylates.^14,22,47^ Me_6_TREN and PMDETA were compared as ligands for MMA polymerization in addition to two initiators, MBiB and 2-bromopropionitrile (BPN). MBiB has previously been used as an initiator in acrylate polymerization, while BPN has been previously used in MMA polymerization.^18,22,48^

Given the known differences in reactivity between acrylates and methacrylates, we sought to determine whether MMA could be effectively polymerized using reagents previously used for acrylates, particularly the same ligands and initiators. Additionally, since MMA represents a distinct monomer class, we aimed to explore how ligand and initiator identity influenced polymerization performance by varying these components while keeping the reagent molar ratios fixed. Three ratios of ligand (1, 0.5, and 0.2), Me_6_TREN or PMDETA, three ratios of CuBr_2_ (0.4, 0.08, and 0.2), and four ratios of ZnTPP (0.02, 0.1, 0.002, and 0.001) were evaluated for MMA polymerization. These 16 unique reagent molar ratios were each tested with both ligands and both initiators (MBiB and BPN), resulting in a total of 64 polymerization conditions with 16 h of light irradiation at 560 nm (5 mW cm^–2^). The results show that combinations of reagents previously used for acrylate polymerization (MBiB and Me_6_TREN) do lead to polymerization of MMA. Some of the ratios attempted here reached MW near the target value, albeit with moderately high *Đ* (>1.44), and some samples exceeded the target *M*_n,(SEC)_ range of 18–24 kDa (Table S11, 12). These results align with reports where acrylate catalytic systems using Me_6_TREN are unsuitable compared to PMDETA or TPMA ligands for methacrylate polymerization.^22,47^ Previous studies using DMSO as solvent have reported that Me_6_TREN exhibits higher activity than TPMA and results in polymers with significantly broader *Đ*, despite similar control over molar mass.^22^ In contrast, well-defined polymers with narrow MW distributions can be obtained for acrylates.^22,37,47^

We compared the effect of different ligands and initiators by comparing the ratio yielding the best result seen from SEC when the ligand or initiator is substituted (ratio No. 14, Tables S11, S12). This shows increased *M*_n(SEC)_ and *Đ* when using MBiB with Me6TREN, as opposed to PMDETA and BPN for MMA polymerization. BPN as the initiator and PMDETA as the ligand resulted in the polymerization of MMA with *M*_n(SEC)_ of 23.4 kDa, *Đ* of 1.26, and conversion of 74% within the target range *M*_n(SEC)_. To further evaluate performance trends, we compared the overall distributions within this chemical space in reference to both *M*_n(SEC)_ and *Đ* (Figure 6c). From this analysis, PMDETA and BPN emerged as the most suitable reagents for high-throughput MMA synthesis and are ideal starting points for any further optimization efforts using this platform. PMDETA, regardless of initiator, resulted in *M*_n(SEC)_ values centered near the target molecular weight, whereas Me_6_TREN consistently produced a higher average *M*_n(SEC)_. While BPN showed a broader spread in *Đ* values, it also enabled much lower *Đ* when paired with PMDETA. In contrast, Me6TREN exhibited a narrower *Đ* distribution but at consistently higher values, suggesting that further improvement with this system may be more challenging. Overall, the trends observed across the full chemical space support previous findings that Me_6_TREN is poorly suited for MMA polymerization, consistently yielding polymers with a high *Đ*. A system suitable for MMA polymerization was identified by applying the platform to systematically screen polymerization conditions. In doing so, we not only identified effective reagent combinations, but also demonstrated how the platform enables broader comparative analyses across a chemical space of interest, supporting the discovery and refinement of new reaction conditions. This effort extended the platform’s application to MMA, which was a new monomer class, highlighting its utility beyond acrylates. Facilitating this type of exploration through automation is particularly valuable in ATRP, where factors such as ligand and initiator identity can substantially influence key parameters like the induction period, *Đ*, and conversion.^22,48^

## 3 Conclusion

The coupling of oxygen-tolerant RDRP, as well as oxygen-resistant chemistries, with automation, such as liquid handling robots, enables high-throughput polymer synthesis and reaction optimization, lowering the knowledge barrier to access advanced polymer materials. To the best of our knowledge, this is the first demonstration of adapting oxygen-tolerant and open-air photo-ATRP chemistries into a high-throughput format by using liquid handling automation. In this work, we have developed a platform to facilitate the translation of photo-ATRP chemistries from the literature to high-throughput settings using a Hamilton STARlet liquid handling robot. We used our automation-assisted photo-ATRP workflow to adapt chemistry from the literature to our high-throughput platform, then generated a data set to demonstrate the potential to integrate ML methods into polymerization reaction optimization by training a classifier to predict initiation from reagent molar ratios. The classifier achieved 88% balanced accuracy in predicting initiation and, after training on only two small batches, reached a perfect prediction on the third. SHAP analysis revealed that ligand and metal catalyst concentrations were the most influential parameters, consistent with an established ATRP mechanistic understanding. This reinforces that the model learned chemically meaningful patterns without prior mechanistic knowledge, validating its use as a tool to lower knowledge barrier in ATRP. The generated data set also showcased our script’s robustness in exploring diverse reagent molar ratios, which is crucial for reaction optimization. For example, adapting open-air photo-ATRP to a high-throughput format required modifying reported conditions for HEA to avoid viscosity issues that are incompatible with automation. Additionally, reagent screening for MMA demonstrated the platform’s applicability across monomer classes and feasibility to systematically explore system components, where findings align with established trends in ligand and initiator selection. This work can pave the way for applying more complex ML approaches, including the integration of probabilistic models like GPRs for polymerization reaction optimization. A significant contribution of this work is the ability to generate data in high throughput, which opens the door to more advanced ML strategies such as active learning and closed-loop optimization of ATRP. Even in the absence of more advanced optimization approaches, automation-assisted photo-ATRP still offers significant improvements in efficiency and accessibility. It enables the translation of chemistries from the literature into a high-throughput setting while lowering the effort and burden of experimental planning. We envision the work presented here will facilitate accessing advanced polymer materials and reaction optimizations by nonexperts and provide an accessible tool to support carrying out experiments at larger scale while lowering the labor and effort required, thus increasing productivity.

## 4 Experimental Section

### 4.1 Materials

Materials and chemicals were purchased from commercial suppliers and used without additional purification unless otherwise stated. Tris­(2-dimethylaminoethyl)­amine (Me_6_TREN, 98+%) and mesitylene (98%) were obtained from Thermo Scientific Chemicals. Copper­(II) bromide (≥99%) was purchased from VWR. 2-Hydroxyethyl acrylate (HEA), methyl methacrylate (MMA, 99%, ≤30 ppm MEHQ as an inhibitor), and methyl acrylate (MA, 99%, ≤100 ppm MEHQ as an inhibitor) were purchased from Sigma-Aldrich. All monomers were stored at 4 °C and purified prior to use by passing through a short column of basic alumina. Deuterated dimethyl sulfoxide (DMSO-*d*_6_, 99.5 atom % D), 5,10,15,20-tetraphenyl-21*H*,23*H*-porphine zinc (ZnTPP), dimethyl sulfoxide (DMSO), eosin Y, 2-bromopropionitrile (BPN), and *N*,*N*,*N*′,*N*″,*N*′′-pentamethyldiethylenetriamine (PMDETA) were also purchased from Sigma-Aldrich. 22.5 mM solutions of CuBr_2_ were sonicated. Among the reagent stocks, only CuBr_2_ required special handling for solubility and was sonicated for 1 h to fully dissolve in DMSO.

### 4.2 Size Exclusion Chromatography (SEC)

Polymer samples were analyzed by size exclusion chromatography (SEC) using an Agilent 1200 Series system with online UV and RI (Agilent 1260 Series) detectors. The system was equipped with a PL Rapide L GPC column (10.0 mm × 100 mm, 3 μm, Agilent) when validating the script and evaluating control over MW as DP increased. For all other samples, the PLgel SEC column (7.5 mm × 300 mm, 5 μm, Agilent) was used. DMF supplemented with 50 mM LiBr was used as the mobile phase. Molecular weight data (*M*_n(SEC)_, MW, and *Đ*) were determined using a series of PMMA standards of known molecular weight (Agilent EasyVial PMMA Calibration Kit) based on the respective RI chromatographs. Sample preparation for SEC utilized the liquid handling robotics for preparing samples when large batches were analyzed; meanwhile, for small batches these were prepared manually. Samples for data collected for script validation (also used in ML training) were prepared using the Hamilton with a custom reagent handling script where 5 μL of reaction solution [M]_f_ = 1 M was added to 980 μL of DMF eluent solution. This procedure was repeated for the PMMA samples with 5 μL of reaction solution [M]_f_ = 2 M added to 980 μL of DMF eluent solution. The samples for the open-air batches, chain extension, and characterization of MW increase with DP were prepared manually. For the open-air samples, since some samples were of high concentration, for samples with [M]_f_ = 4.5 M, 11 μL of reaction solution was used, and for those with [M]_f_ = 3.5 M, 14 μL was used in a total volume of 1 mL in SEC eluent solution. This was done to prevent column overload by high concentration samples. All samples displaying high viscosity that could hinder proper aspiration by the robot were prepared manually.

### 4.3 Nuclear Magnetic Resonance (NMR)

Samples were diluted in *d*_6_-DMSO using mesitylene as a reference standard and characterized using a Varian VNMRS 500 MHz spectrometer. For open-air ATRP and MMA polymerization, NMR was only performed on the best performing samples.

### 4.4 Optical Power Measurements of Light Boxes

A Newport 1917-R optical power meter with a 3D printed head that only measures one well at a time was used to find the average optical power at a specified wavelength; for the light box using for green light polymerization, optical measurements were done at 515 nm. The light box used for polymerizations at 560 nm was previously characterized.^29^

### 4.5 Polymerizations

The components of the automated polymer synthesis platform available in our lab are controlled by a custom-made master Python script referred to as Polycraft. The master Python script enables the interface between the liquid handling robot and user inputs, which are provided in an excel sheet. Polymerizations were carried out using a Hamilton STARlet liquid handling robot that utilized Polycraft to generate transfer steps for the liquid handling robot. The volumes and concentrations of each reagent to use were determined using the ATRP script presented in this work. For each optimizable reagent, high concentration stocks were prepared, and the script was informed that it could use a range of concentrations that can be obtained by serial dilution. CuBr_2_ was used at concentrations up to 22.5 mM, with sonication for 1 h required to achieve complete dissolution. The highest concentrations used were 200 mM for Me_6_TREN, 4 mM for ZnTPP, and 2 mM for Eosin Y. All polymerizations were carried out in 96-well plates, unstirred, with 100 μL of oxygen headspace. For oxygen-tolerant polymerization, the plates were sealed using an adhesive plate seal; meanwhile, those for open-air chemistry were not sealed. During the platform validations using open-air chemistry and MMA polymerization, the target DP was set to 200, and polymerization proceeded for 16 h with [M]_f_ at 2 M. The evaluation of open-air photo-ATRP was done by attempting polymerization with [M]_f_ at 5.5 M and HEA/MBiB/CuBr_2_/Me_6_TREN/EYH_2_ = 200/1/0.05/0.3/0.005 with SEC characterization at 1 and 5 h of green light radiation. All polymerizations were carried out at room temperature using polypropylene plates. For polymerization of different acrylate polymers and statistical copolymers, [M]_f_ was set to 1 M with 10 h of light radiation.

### 4.6 Chain Extension of pHEA_50_ with MA

The macroinitiator was synthesized using the reagent molar ratio HEA/MBiB/CuBr_2_/Me_6_TREN/ZnTPP = 50/1/0.08/0.2/0.002. [M]_f_ was 2 M in a total volume of 200 μL with 100 μL of oxygen headspace. The macroinitiator was placed on the light box for 1.5 h, then the sealing tape was removed, and the contents of the well were mixed with a pipet followed by the removal of 36.00 μL from the reaction mixture in the well. Finally, 36.00 μL of MA at an 11.105 M stock concentration was added to the well, and the reaction solution was mixed with the pipet, sealed with sealing tape, and placed on the light box for 8 h.

### 4.7 ML Classifier

Binary classification was performed by using an SGD classifier from scikit-learn. Data were collected on three different batches where each batch was used in the order it was collected. The first batch was used to train a model to predict outcomes for the second batch, followed by combining the data from the first two batches and retraining the model to predict outcomes for the third batch, thereby simulating the application of ML in a sequential reaction optimization setting.

The data was assigned binary labels based on results from NMR or SEC, where samples that initiated were assigned 1 as the label and those that did not were assigned 0. The first batch consisted of 42 samples, the second batch consisted of 37 samples, and the third batch consisted of 88 samples. The input features were the reagent molar ratios of Me_6_TREN, ZnTPP, and CuBr_2_ with respect to initiator concentration, which was fixed for a target DP of 200. The input features were normalized using scikit-learn’s *StandardScaler­()*. The training data for each model was randomly split with an 80/20 split, using 20% for testing using 5-fold cross validation and hyperparameter tuning done using *RandomSearchCV­().* Following hyperparameter tuning and evaluation of the model on the train test set, the model was used to predict the label in the second batch. After this, the first two batches were combined, and the model was retrained and used to predict the third batch. Classification metrics were obtained during model evaluation, but only balanced accuracy was used for final evaluation given the presence of class imbalance in the data set.

## Supplementary Material



## Data Availability

The source code for polymerization planning is available on GitHub. https://github.com/GormleyLab/polymerization_planner.

## Notes

Sample inputs to polymerization planning script and logic, sample SEC and NMR for ML label assignments, polymerization conditions attempted on open-air and methacrylate chemistries and resulting *M*_n(SEC)_ and *Đ* (PDF)

## Abbreviations

ARGET: activators regenerated by electron transfer
ATRP: atom-transfer radical polymerization
BPN: 2-bromopropionitrile
CTA: chain transfer agent
Cu: copper
CuBr_2_: copper­(II) bromide
DMSO: dimethyl sulfoxide
DP: degree of polymerization
Enz-RAFT: enzyme degassing of RAFT
EYH_2_: eosin y
GPR: Gaussian process regressor
HEA: 2-hydroxylethyl acrylate
HPA: 2-hydroxypropyl acrylate
L: ligand
MA: methyl acrylate
MBiB: methyl α-bromoisobutyrate
[M]_f_: final monomer concentration
MEHQ: monomethyl ether of hydroquinone
Me_6_TREN: tris­(2-dimethylaminoethyl)­amine
ML: machine learning
methyl MMA: methyl methacrylate
MW: molecular weight
NMR: nuclear magnetic resonance
PC: photocatalyst
PET-RAFT: photoinduced electron/energy transfer reversible addition–fragmentation chain-transfer polymerization
PMDETA: *N*,*N*,*N*′,*N*″,*N*′′-pentamethyldiethylenetriamine
RDRP: reversible-deactivation radical polymerization
SEC: size-exclusion chromatography
ZnTPP: zinc­(II) tetraphenylporphyrin
