# Supplementary material for: Automation-Assisted Photoinduced Atom Transfer Radical Polymerization
Source: ACS Polym Au. 2025 Aug 28;6(1):181–93. doi: 10.1021/acspolymersau.5c00067 (PMC12903423; doi:10.1021/acspolymersau.5c00067)
Supplement: Supplementary file 1 [file lg5c00067_si_001.pdf]

Supplementary Information

Automation-Assisted Photo-ATRP

Cesar Ramirez, Eman Ahmed, Elena Di Mare, Maria Pineiro-Goncalves, Apostolos Maroulis, Prajakatta Mulay, D. Christopher Radford, Adam J. Gormley*

Department of Biomedical Engineering, Rutgers, The State University of New Jersey, Piscataway, NJ 08854, USA

*Correspondence to:
Adam J. Gormley: adam.gormley@rutgers.edu

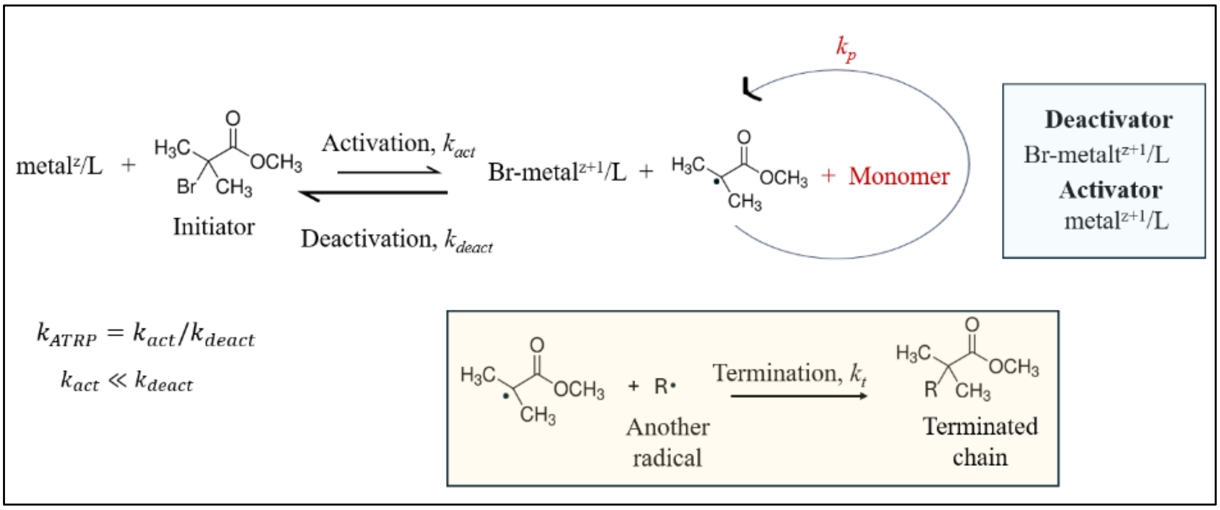

Figure S1. ATRP mechanism. During activation the lower oxidation state metal^z/L complex reacts with the alkyl halide forming a complex with a coordinated halogen anion and a radical in a reversible process. This can react with additional monomers during propagation or another radical and lead to termination. In this schematic methyl-α-bromoisobutyrate represents the alkyl halide initiator.

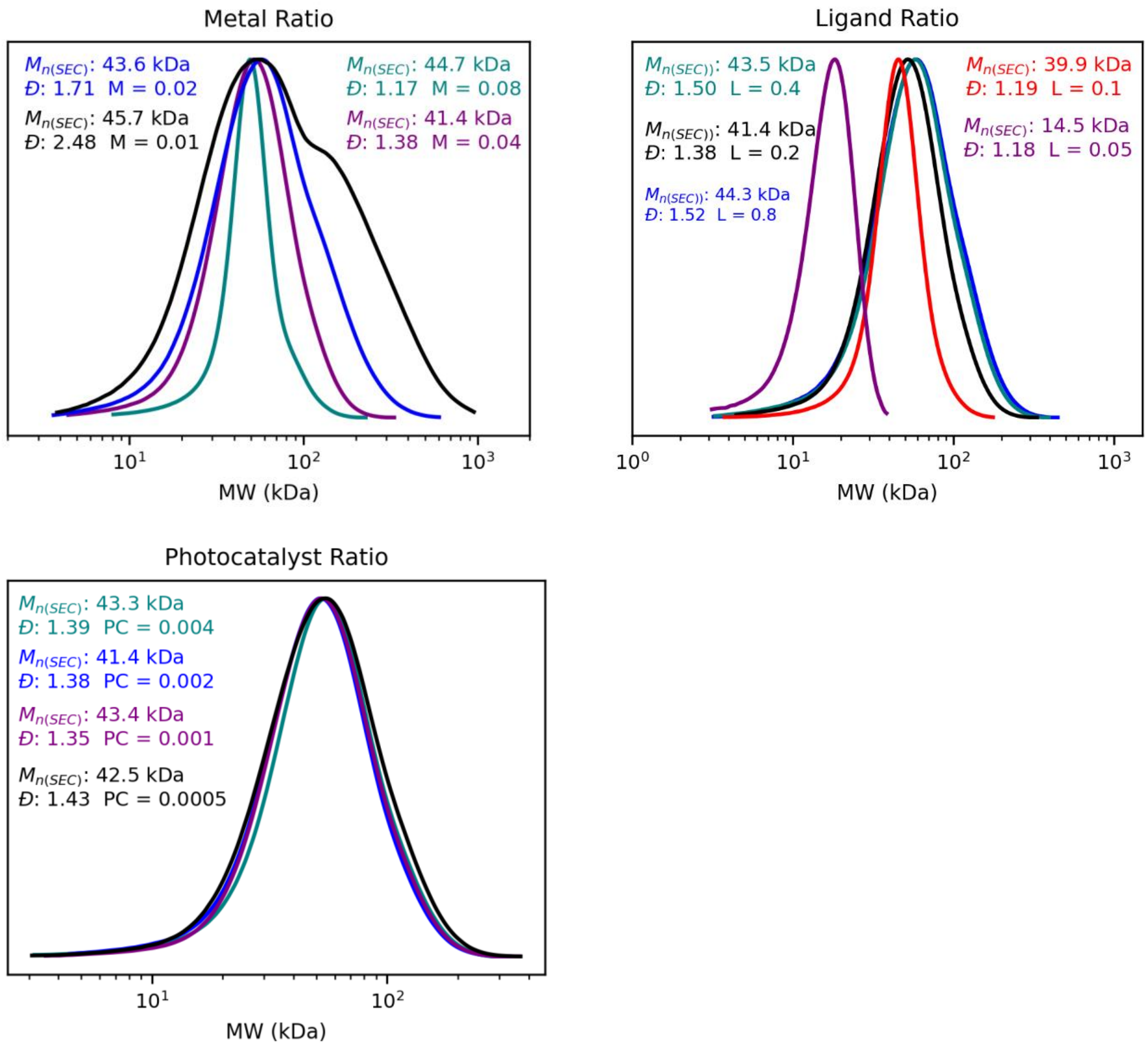

Figure S2. Exploration of reagent molar ratios and their effect on polymerization reactions as determined by size-exclusion chromatography (SEC). The molar ratios for each reagent are shown in the figure, and the final monomer (HEA) concentration was set at 1 M. Showing the effects as the original ratio of 200/1/0.04/0.02/0.002 (HEA/MBiB/$CuBr_2$/Me_6TREN/ZnTPP) were changed for each reagent while keeping other reagent ratios constant. Results indicate ligand (L) and metal(M) have greater influence on polymerization outcome compared to the photocatalyst (PC).

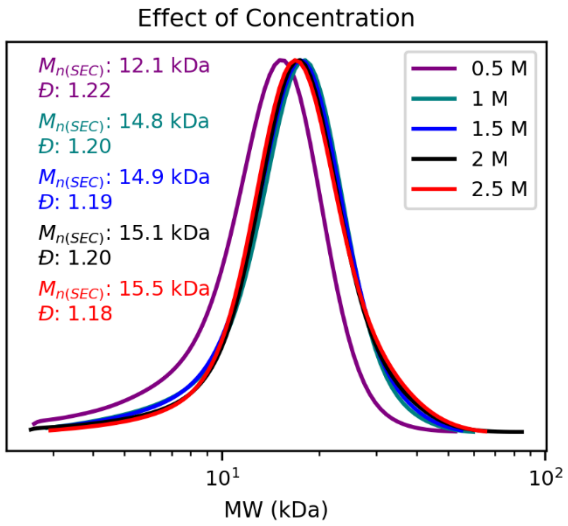

Figure S3. Polymerization of $pHEA_{50}$ at final monomer concentrations of 0.5-2.5 M.

Table S1. Molar ratios for metal, ligand and photo catalyst tested during script validation. Final monomer concentration was fixed at 1M.

Photo Catalyst	Ligand	Metal
0.001	0.04	0.025
0.002	0.1	0.05
0.004	0.2	0.1
0.01	0.4	0.2
0.02	1	0.4
0.04	2	
0.1		
0.2		
0.4		

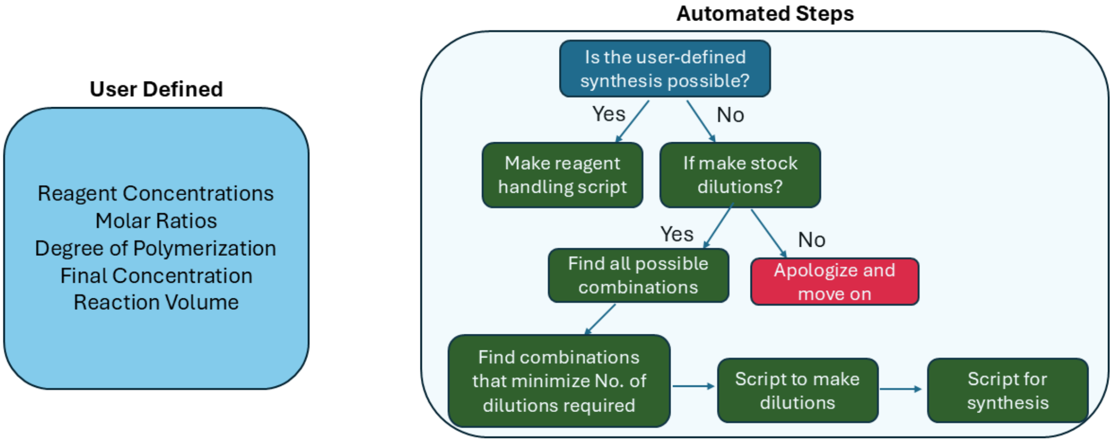

Figure S4. User-defined inputs provided to script in an Excel sheet and logic behind reagent handling for synthesis script creation.

Table S2. Sample input to script, for single monomer polymers and reagent optimization experiments. User provides reagent molar ratios, the concentration of each reagent stock solution, the final monomer concentration, and reaction volume. Concentrations are in mM unless otherwise stated, and volume in µL. M_f is the final monomer concentration.

**Sample Sheet**	**Reagent Ratios**					**Info on Highest Available Stock Concentration**						
Polymer ID	Monomer	Initiator	Metal	Ligand	PC	[Monomer] (M)	[I] (mM)	[Metal] (mM)	[L] (mM)	[PC] (mM)	M_f (M)	Volume
1	25	1	0.08	0.2	0.00 2	11.1	200	22.5	100	4	1	200
2	75	1	0.08	0.4	0.00 2	11.1	200	22.5	100	4	1	150
3	125	1	0.04	0.2	0.00 2	11.1	200	22.5	100	4	2	300
4	150	1	0.08	1	0.00 2	11.1	200	22.5	100	4	2	200

Table S3. User-defined excel sheet for copolymer synthesis of up to four different monomers if only homopolymers are synthesized the remaining columns can be left blank. The order should correspond to the entries for molar ratios in **Table S2** and all monomer stock concentrations should be the same. M_f is the final monomer concentration in mM, Vol is the final reaction volume.

Polymer ID	Mon 1	Mon 1%	Mon 2	Mon 2%	Mon 3	Mon 3%	Mon 4	Mon 4%
1	HEA							
2	HEA	50	MA	50				
3	HEA							
4	HEA							

Table S4. Data frame with information on reagent stock dilutions necessary to conduct experiment on polymer ratios to synthesize.

[Metal] (mM)	Metal Volumes (µL)	[Ligand] (mM)	Ligand Volumes (µL)	[PC] (mM)	PC Volumes (µL)
5.625	262.23	50	412	0.5	296
22.5	271.12	6.25	212.8	4	375
2.8125	262.23	12.5	272	0.25	288
11.25	271.12	25	240	2	205
				0.125	216

Table S5. Data frame with information on the polymer ratios to test, along with the volume and concentration required for each reagent for a given sample. The molar ratios are in the columns as HEA/MBiB/Metal/Ligand/PC. The stock concentration in mM for each reagent is given in brackets [], M_f is the final monomer concentration in mM, Vol is the final reaction volume.

HEA	MBiB	Metal	Ligand	PC	[M] (M)	[I] (mM)	[Metal] (mM)	[L] (mM)	[PC] (mM)	M_f (M)	Vol (µL)
200	1	0.05	0.4	0.02	2	55	5.6	50	0.5	1	200
200	1	0.1	0.04	0.1	2	55	5.6	6.25	4	1	200
200	1	0.4	0.1	0.2	2	55	22.5	12.5	4	1	200
200	1	0.05	1	0.04	2	55	2.8	25	4	1	200
200	1	0.2	0.4	0.004	2	55	11.3	50	0.3	1	200
200	1	0.05	0.2	0.04	2	55	5.6	12.5	4	1	200
200	1	0.05	0.1	0.004	2	55	2.8	12.5	0.5	1	200
200	1	0.025	0.1	0.01	2	55	2.8	12.5	2	1	200
200	1	0.4	2	0.001	2	55	22.5	50	0.1	1	200
200	1	0.4	2	0.004	2	55	22.5	50	0.3	1	200
200	1	0.05	0.04	0.001	2	55	5.6	6.25	0.1	1	200
200	1	0.2	0.4	0.002	2	55	11.3	50	0.3	1	200
200	1	0.05	0.1	0.2	2	55	5.6	12.5	4	1	200
200	1	0.1	1	0.004	2	55	11.3	50	0.3	1	200
200	1	0.2	1	0.004	2	55	11.3	50	0.3	1	200
200	1	0.4	0.2	0.1	2	55	22.5	12.5	4	1	200
200	1	0.1	2	0.004	2	55	11.3	50	0.3	1	200
200	1	0.025	1	0.02	2	55	2.8	50	0.5	1	200
200	1	0.05	0.1	0.02	2	55	5.6	12.5	4	1	200
200	1	0.025	0.4	0.004	2	55	2.8	50	0.5	1	200

Table S5 Continued. The volumes required for each reagent given their concentration in µL.

HEA	MBiB (µL)	Metal (µL)	Ligand (µL)	PC (µL)	DMSO (µL)
100	18.18	8.89	8	40	24.93
100	18.18	17.78	6.4	25	32.64
100	18.18	17.78	8	50	6.04
100	18.18	17.78	40	10	14.04
100	18.18	17.78	8	16	40.04
100	18.18	8.89	16	10	46.93
100	18.18	17.78	8	8	48.04
100	18.18	8.89	8	5	59.93
100	18.18	17.78	40	8	16.04
100	18.18	17.78	40	16	8.04
100	18.18	8.89	6.4	8	58.53
100	18.18	17.78	8	8	48.04
100	18.18	8.89	8	50	14.93
100	18.18	8.89	20	16	36.93
100	18.18	17.78	20	16	28.04
100	18.18	17.78	16	25	23.04
100	18.18	8.89	40	16	16.93
100	18.18	8.89	20	40	12.93
100	18.18	8.89	8	5	59.93
100	18.18	8.89	8	8	56.93

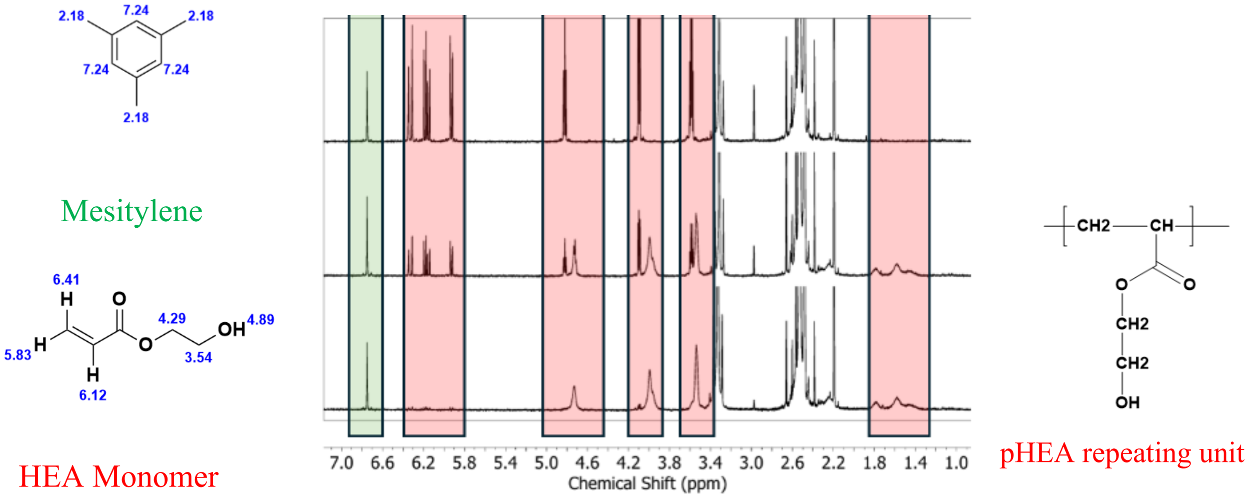

Figure S5. NMR spectra showing examples where polymerization did not occur (top), where some has occurred (middle) and when all monomer has been converted (bottom). The middle panel shows the peak shifting due to differences between protons in the monomer versus the polymer. Shifts from polymer formation can be observed by up field shifts in the NMR peaks of protons belonging to pHEA versus those belonging to HEA monomer are both shown in red. The spectra of pHEA also has a broad peak pertaining to the CH and CH_2 protons in the repeating unit around 1.4-1.8 ppm. Mesitylene standard is shown in green. Spectra collected using d_6-DMSO as the solvent.

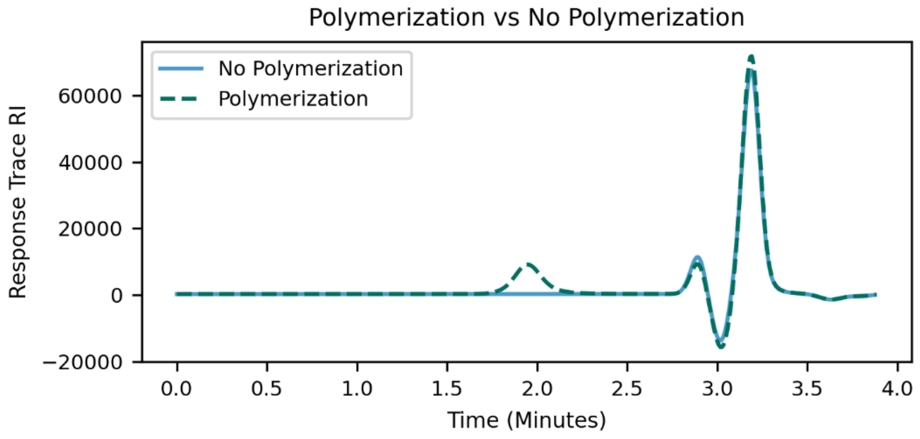

Figure S6. Sample SEC traces using a high-throughput column. When no polymerization occurred (blue) versus when this occurred (green).

Table S6. Samples that showed a signal in the NMR spectra or an SEC trace indicating polymerization and those that did not and therefore considered not initiated in each batch.

Batch #	1	2	3
Polymerized	31	25	77
Unpolymerized	11	12	11
Total	42	37	88

Table S7. Performance of the model trained on data from the first batch used to predict on outcome from the second batch. There were 42 samples in the first batch with 20% set aside as the train test set.

	Accuracy	Balanced Accuracy	Recall	Precision	ROC AUC
Train test set (batch 1)	1	1	1	1	1
Test set (batch 2)	0.91	0.88	1	0.89	0.88

Table S8. Performance of model trained on data from the first two batches used to predict on outcomes from the third batch. There were 79 samples in the first two batches with 20% held out as the train test set.

	Accuracy	Balanced Accuracy	Recall	Precision	ROC AUC
Train test set (batch 1 and 2)	1	1	1	1	1
Experimental (batch 3)	1	1	1	1	1

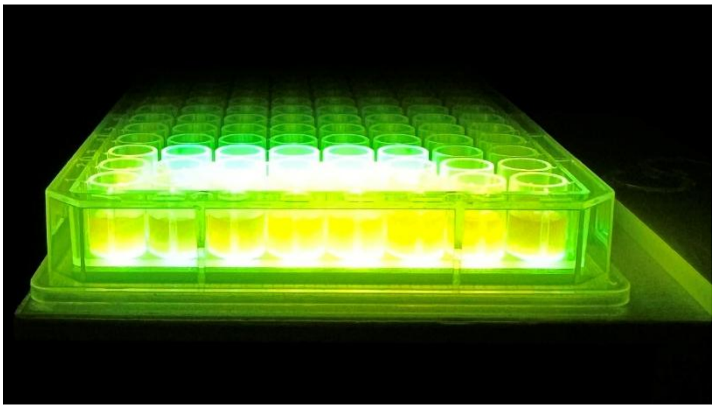

Figure S7. Setup used for open-air polymerization experiments of $pHEA_{200}$ using custom green LED lightbox

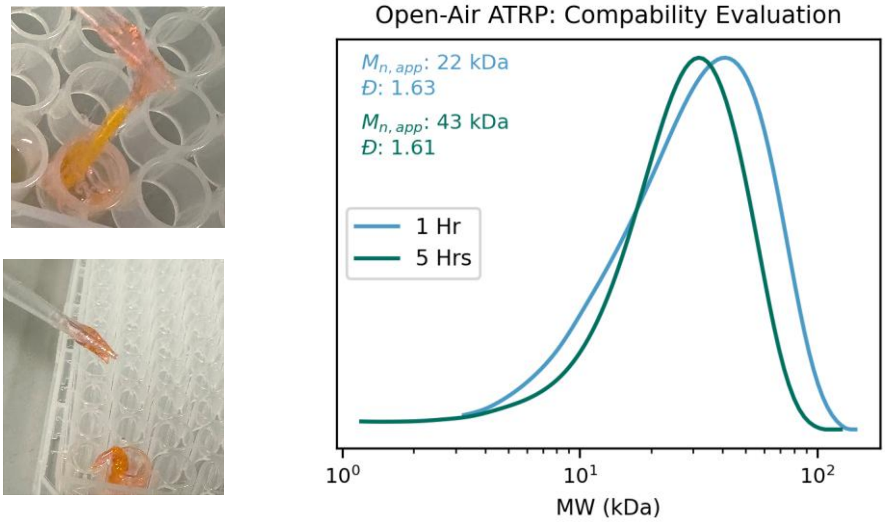

Figure S8. Open-air polymerization of $pHEA_{200}$ at one and five hours, with a final monomer concentration of 5.5 M using the following molar ratio HEA/MBiB/$CuBr_2$/Me_6TREN/EYH_2 = 200/1/0.05/0.3/0.005, resulting in a highly viscous $pHEA_{200}$.[1] The $M_{n(SEC)}$ was near the target range of 45-55 kDa following five hours of light irradiation.

Table S9. Seven reagent molar ratios attempted during the second round to transition an open-air photo-ATRP system into a format compatible with high-throughput synthesis. Three different final monomer concentrations were tested for each ratio to determine if polymerization at lower final monomer concentrations is feasible for the proceeding round.

ID	Monomer	MBiB	$CuBr_2$	Me_6TREN	EYH_2	$[M]_f$ (M)	$M_{n(SEC)}$ (kDa)	*Đ*
1A	200	1	0.05	0.3	0.01	4.5	89.8	2.32
2A	200	1	0.05	0.3	0.005	4.5	93.9	2.12
3A	200	1	0.02	0.12	0.001	4.5	81.4	2.03
4A	200	1	0.02	0.12	0.0002	4.5	-	-
5A	200	1	0.02	0.3	0.005	4.5	99.8	2.54
6A	200	1	0.08	0.3	0.005	4.5	98.7	1.98
7A	200	1	0.05	0.2	0.005	4.5	94.9	1.97
1B	200	1	0.05	0.3	0.01	3.5	100.5	2.01
2B	200	1	0.05	0.3	0.005	3.5	93.7	1.95
3B	200	1	0.02	0.12	0.001	3.5	77.1	1.96
4B	200	1	0.02	0.12	0.0002	3.5	-	-
5B	200	1	0.02	0.3	0.005	3.5	89.3	2.18
6B	200	1	0.08	0.3	0.005	3.5	80.2	1.81
7B	200	1	0.05	0.2	0.005	3.5	84.5	1.94
1C	200	1	0.05	0.3	0.01	2.5	84.1	1.70
2C	200	1	0.05	0.3	0.005	2.5	84.2	1.62
3C	200	1	0.02	0.12	0.001	2.5	NA	NA
4C	200	1	0.02	0.12	0.0002	2.5	NA	NA
5C	200	1	0.02	0.3	0.005	2.5	79.6	1.98
6C	200	1	0.08	0.3	0.005	2.5	-	-
7C	200	1	0.05	0.2	0.005	2.5	-	-

Table S10. SEC results from the third round to transition an open-air photo-ATRP system into a format compatible with high-throughput synthesis polymerization of HEA. The input to the script was DP 100 to double the initiator concentration to decrease $M_{n(SEC)}$. Polymerizations were carried out with five hours of green light irradiation. The final monomer concentration was set at 2.5 M.

Monomer	MBiB	$CuBr_2$	Me_6TREN	EYH_2	$M_{n(SEC)}$ (kDa)	*Đ*
200	1	0.100	0.3	0.005	-	-
200	1	0.150	0.3	0.005	-	-
200	1	0.025	0.3	0.005	82.6	1.92
200	1	0.017	0.3	0.005	91.0	2.04
200	2	0.025	0.3	0.005	48.6	1.46
200	2	0.017	0.3	0.005	50.4	1.62
200	2	0.050	0.3	0.005	47.3	1.24
200	2	0.100	0.3	0.005	42.1	1.35
200	1	0.050	0.15	0.005	NA	NA
200	1	0.050	0.1	0.005	NA	NA
200	1	0.050	0.6	0.005	81.6	1.55
200	2	0.050	0.6	0.005	45.9	1.48

Table S11. SEC results from molar ratios tested for the polymerization of $pMMA_{200}$ at a final monomer concentration of 2 M using Me_6TREN as the ligand and MBiB or BPN as the initiator. Using MMA/Initiator/$CuBr_2$/Me_6TREN/ZnTPP indicated below. MMA and initiator ratio were fixed at 200 and 1 respectively. The ID number denotes the reagent molar ratio, whereas the letter denotes the initiator A = MBiB and B = BPN.

ID	$CuBr_2$	Me_6TREN	ZnTPP	Initiator	$M_{n(SEC)}$ (kDa)	*Đ*
1A	0.4	1	0.02	MBiB	11.5	1.49
2A	0.08	1	0.1	MBiB	15.1	1.96
3A	0.08	0.5	0.02	MBiB	36.9	1.53
4A	0.2	1	0.1	MBiB	34.4	1.78
1B	0.4	1	0.02	BPN	27.7	1.60
2B	0.08	1	0.1	BPN	35.8	1.95
3B	0.08	0.5	0.02	BPN	36.2	1.61
4B	0.2	1	0.1	BPN	19.1	1.65
5A	0.08	1	0.02	MBiB	43.4	1.52
6A	0.4	1	0.1	MBiB	40.5	1.78
7A	0.08	0.5	0.002	MBiB	29.3	1.61
8A	0.2	1	0.02	MBiB	44.9	1.61
5B	0.08	1	0.02	BPN	34.4	1.95
6B	0.4	1	0.1	BPN	23.9	1.78
7B	0.08	0.5	0.002	BPN	33.4	1.75
8B	0.2	1	0.02	BPN	16.7	1.66
9A	0.2	1	0.002	MBiB	39.0	1.45
10A	0.08	0.2	0.1	MBiB	23.2	1.52
11A	0.2	1	0.001	MBiB	34.5	1.51
12A	0.08	0.5	0.1	MBiB	37.8	1.63
9B	0.2	1	0.002	BPN	41.7	1.59
10B	0.08	0.2	0.1	BPN	15.1	1.55
11B	0.2	1	0.001	BPN	36.2	1.51
12B	0.08	0.5	0.1	BPN	33.6	1.78
13A	0.08	0.2	0.02	MBiB	24.7	1.51
14A	0.2	0.5	0.1	MBiB	34.9	1.52
15A	0.08	1	0.002	MBiB	39.7	1.57
16A	0.2	1	0.001	MBiB	37.2	1.53
13B	0.08	0.2	0.02	BPN	16.0	1.52
14B	0.2	0.5	0.1	BPN	27.7	1.54
15B	0.08	1	0.002	BPN	51.0	1.86
16B	0.2	1	0.001	BPN	22.0	1.59

Table S12. SEC results from molar ratios tested for the polymerization of $pMMA_{200}$ at a final monomer concentration of 2 M with PMDETA as the ligand and MBiB or BPN as the initiator. Using MMA/Initiator/$CuBr_2$/PMDETA/ZnTPP indicated below. MMA and initiator ratio were fixed at 200 and 1 respectively. The ID number denotes the reagent molar ratio, whereas the letter denotes the initiator A = MBiB and B = BPN.

ID	Metal	PMDETA	ZnTPP	Initiator	$M_{n(SEC)}$ (kDa)	*Đ*
1A	0.4	1	0.02	MBiB	11.5	1.59
2A	0.08	1	0.1	MBiB	12.8	1.86
3A	0.08	0.5	0.02	MBiB	21.5	1.85
4A	0.2	1	0.1	MBiB	26.7	1.92
1B	0.4	1	0.02	BPN	25.2	1.58
2B	0.08	1	0.1	BPN	16.4	1.78
3B	0.08	0.5	0.02	BPN	16.0	1.77
4B	0.2	1	0.1	BPN	36.9	1.73
5A	0.08	1	0.02	MBiB	14.7	1.94
6A	0.4	1	0.1	MBiB	20.1	1.73
7A	0.08	0.5	0.002	MBiB	24.8	1.70
8A	0.2	1	0.02	MBiB	32.8	1.80
5B	0.08	1	0.02	BPN	18.7	1.85
6B	0.4	1	0.1	BPN	41.5	1.50
7B	0.08	0.5	0.002	BPN	28.5	1.62
8B	0.2	1	0.02	BPN	30.1	1.84
9A	0.2	1	0.002	MBiB	-	-
10A	0.08	0.2	0.1	MBiB	24.2	1.51
11A	0.2	1	0.001	MBiB	30.0	1.60
12A	0.08	0.5	0.1	MBiB	32.4	1.65
9B	0.2	1	0.002	BPN	20.3	1.60
10B	0.08	0.2	0.1	BPN	14.8	1.40
11B	0.2	1	0.001	BPN	11.1	1.37
12B	0.08	0.5	0.1	BPN	29.0	1.55
13A	0.08	0.2	0.02	MBiB	25.7	1.53
14A	0.2	0.5	0.1	MBiB	27.4	1.52
15A	0.08	1	0.002	MBiB	46.2	1.49
16A	0.2	1	0.001	MBiB	32.9	1.54
13B	0.08	0.2	0.02	BPN	15.1	1.40
14B	0.2	0.5	0.1	BPN	23.4	1.26
15B	0.08	1	0.002	BPN	44.6	1.40
16B	0.2	1	0.001	BPN	12.2	1.36

References

1. Szczepaniak, G.; Jeong, J.; Kapil, K.; Dadashi-Silab, S.; Yerneni, S. S.; Ratajczyk, P.; Lathwal, S.; Schild, D. J.; Das, S. R.; Matyjaszewski, K., Open-air green-light-driven ATRP enabled by dual photoredox/copper catalysis. *Chemical Science* **2022,** *13* (39), 11540-11550.

Appendix 1A: SEC Traces for Validation/ML Data (Batch 1)

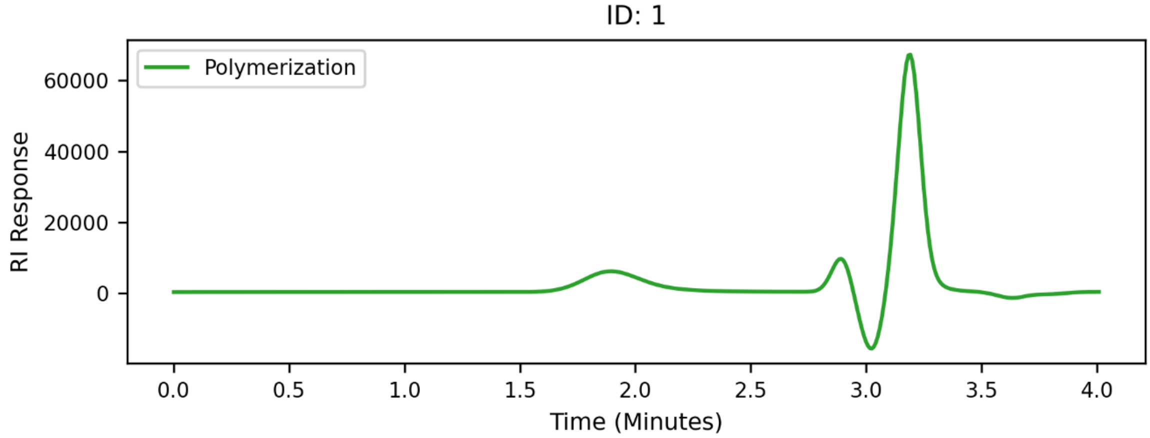

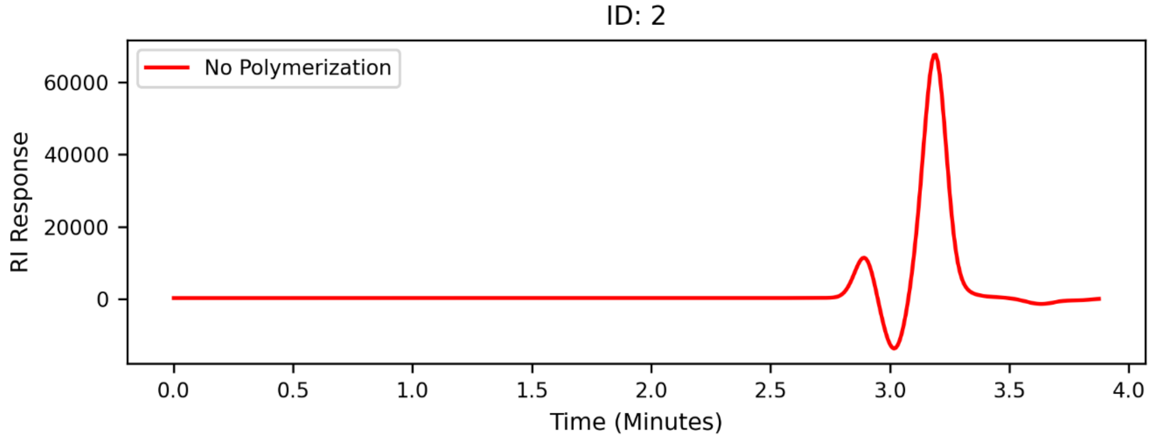

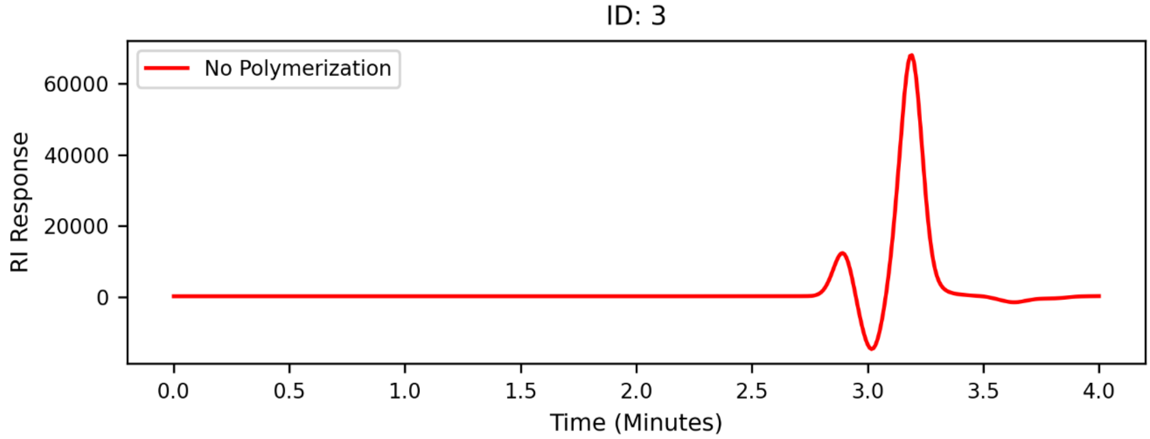

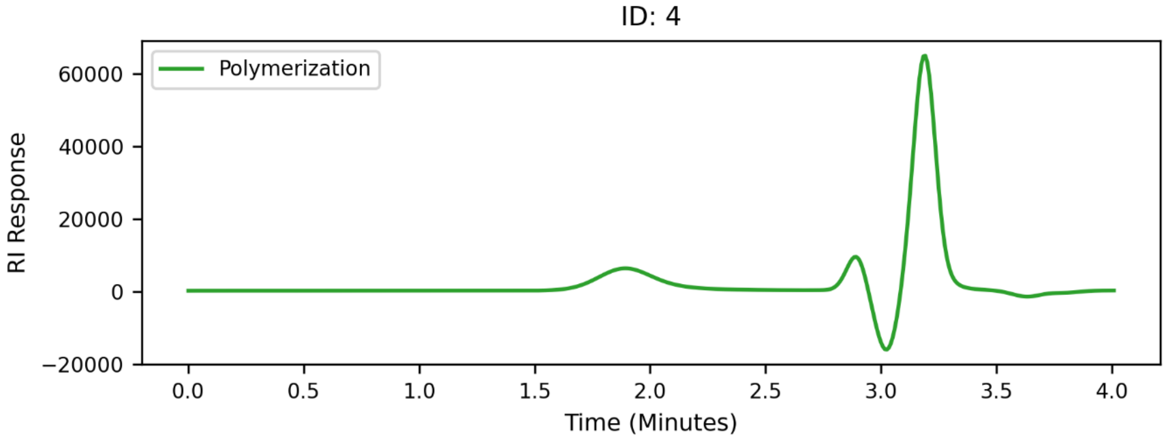

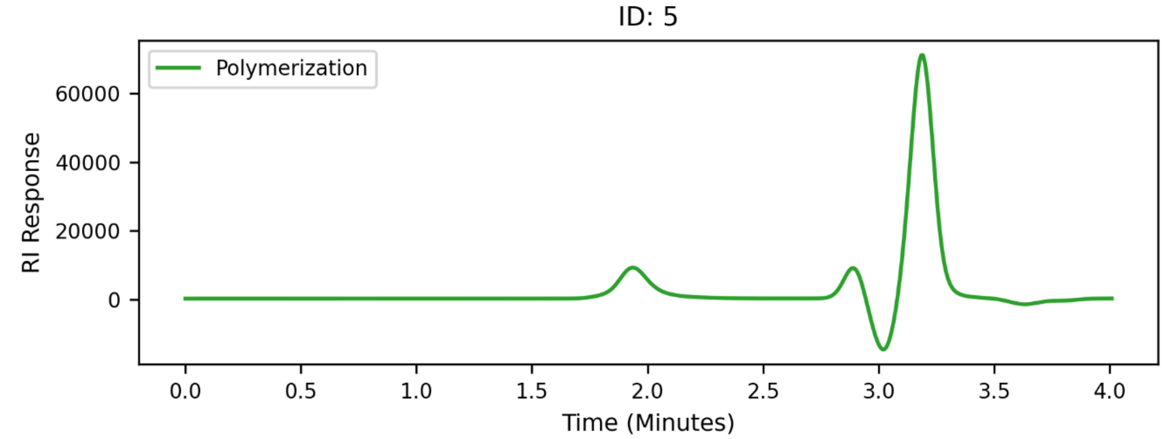
ID: 5
Polymerization
RI Response
60000
40000
20000
0
0.0
0.5
1.0
1.5
2.0
2.5
3.0
3.5
4.0
Time (Minutes)

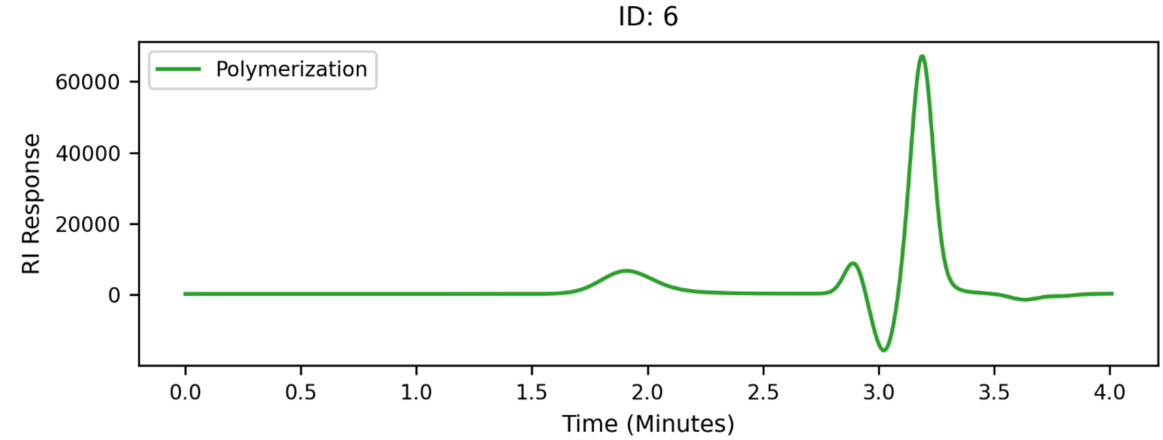
ID: 6
Polymerization
RI Response
60000
40000
20000
0
0.0
0.5
1.0
1.5
2.0
2.5
3.0
3.5
4.0
Time (Minutes)

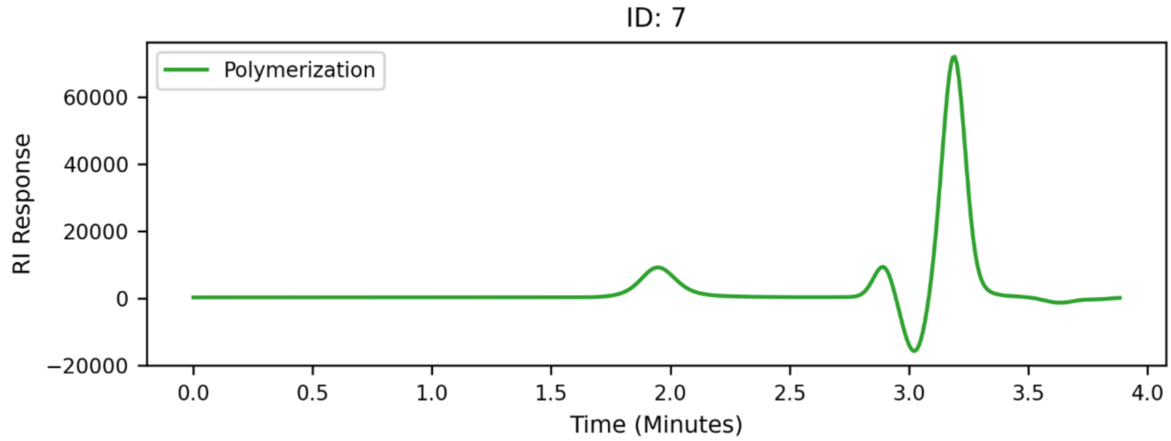
ID: 7
Polymerization
RI Response
60000
40000
20000
0
−20000
0.0
0.5
1.0
1.5
2.0
2.5
3.0
3.5
4.0
Time (Minutes)

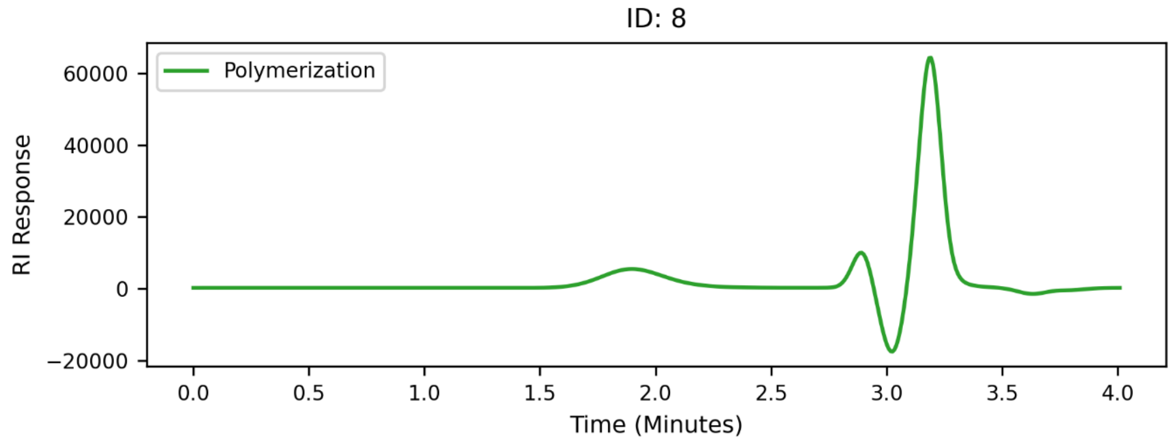
ID: 8
Polymerization
RI Response
60000
40000
20000
0
−20000
0.0
0.5
1.0
1.5
2.0
2.5
3.0
3.5
4.0
Time (Minutes)

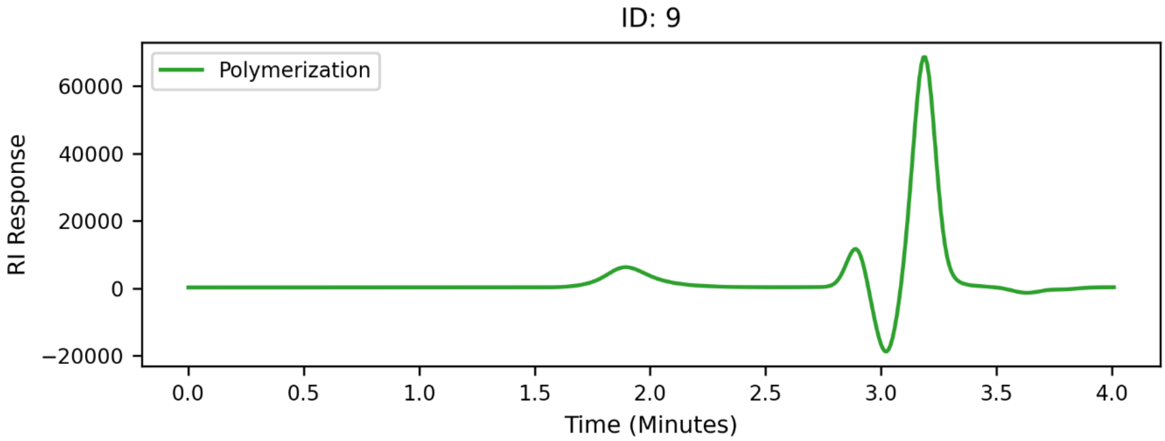
ID: 9
Polymerization
RI Response
Time (Minutes)
60000
40000
20000
0
−20000
0.0
0.5
1.0
1.5
2.0
2.5
3.0
3.5
4.0

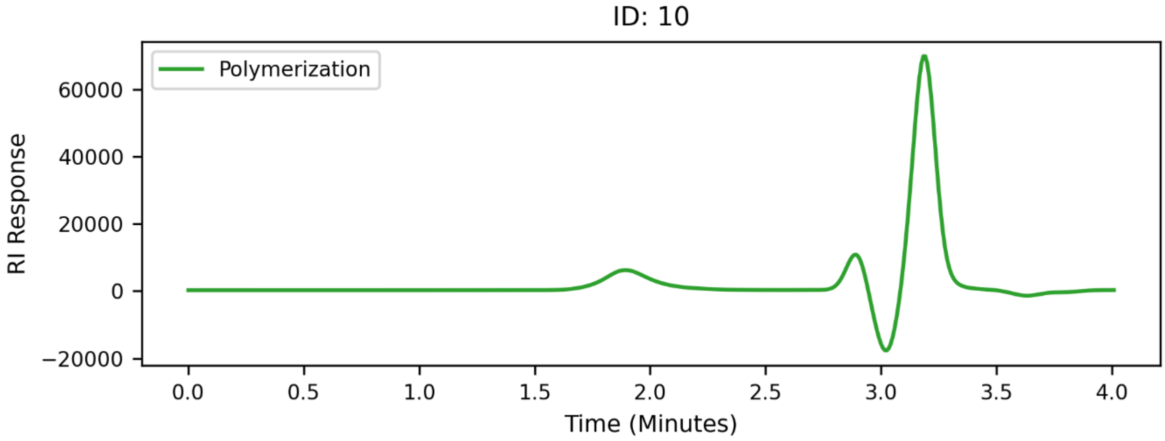
ID: 10
Polymerization
RI Response
Time (Minutes)
60000
40000
20000
0
−20000
0.0
0.5
1.0
1.5
2.0
2.5
3.0
3.5
4.0

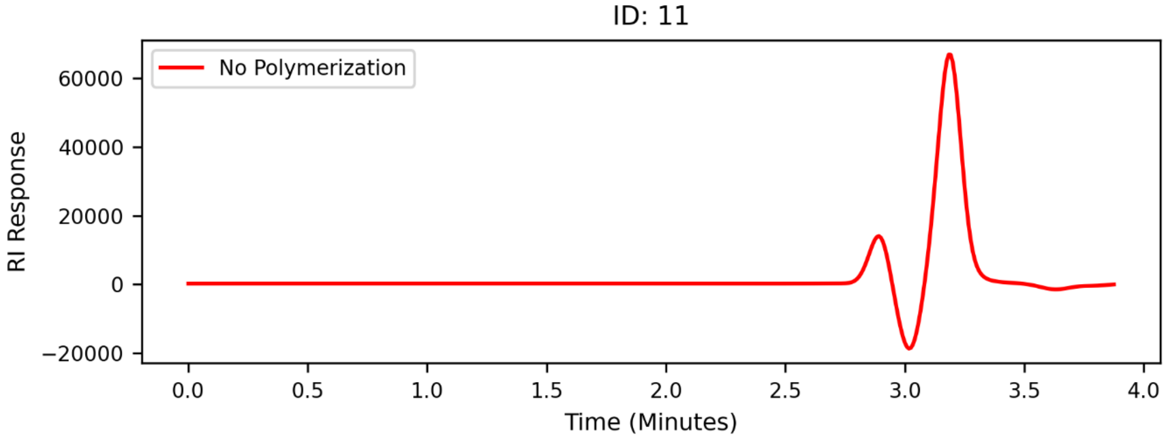
ID: 11
No Polymerization
RI Response
Time (Minutes)
60000
40000
20000
0
−20000
0.0
0.5
1.0
1.5
2.0
2.5
3.0
3.5
4.0

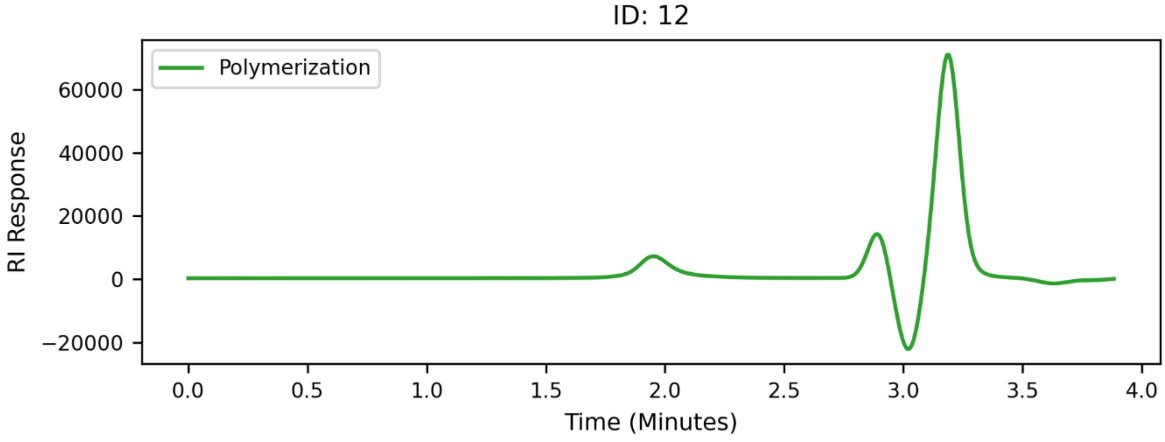
ID: 12
Polymerization
RI Response
Time (Minutes)
60000
40000
20000
0
−20000
0.0
0.5
1.0
1.5
2.0
2.5
3.0
3.5
4.0

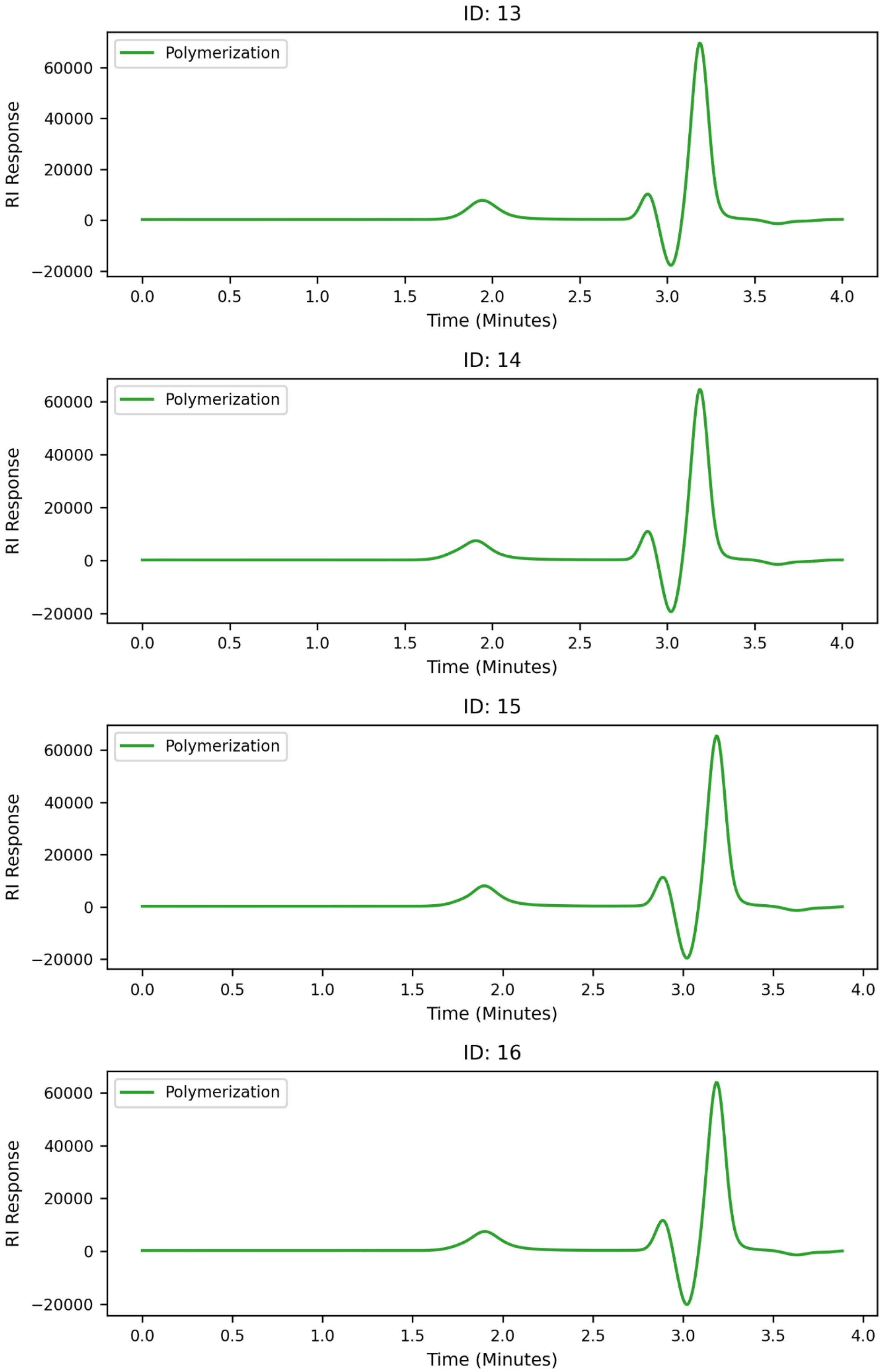

ID: 13
Polymerization
RI Response
Time (Minutes)
ID: 14
Polymerization
RI Response
Time (Minutes)
ID: 15
Polymerization
RI Response
Time (Minutes)
ID: 16
Polymerization
RI Response
Time (Minutes)

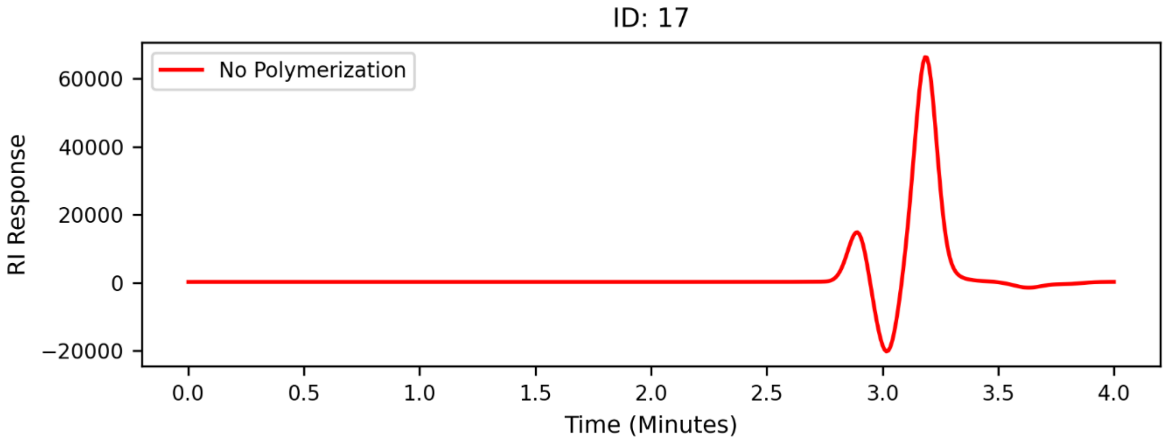
ID: 17
No Polymerization
RI Response
60000
40000
20000
0
−20000
0.0
0.5
1.0
1.5
2.0
2.5
3.0
3.5
4.0
Time (Minutes)

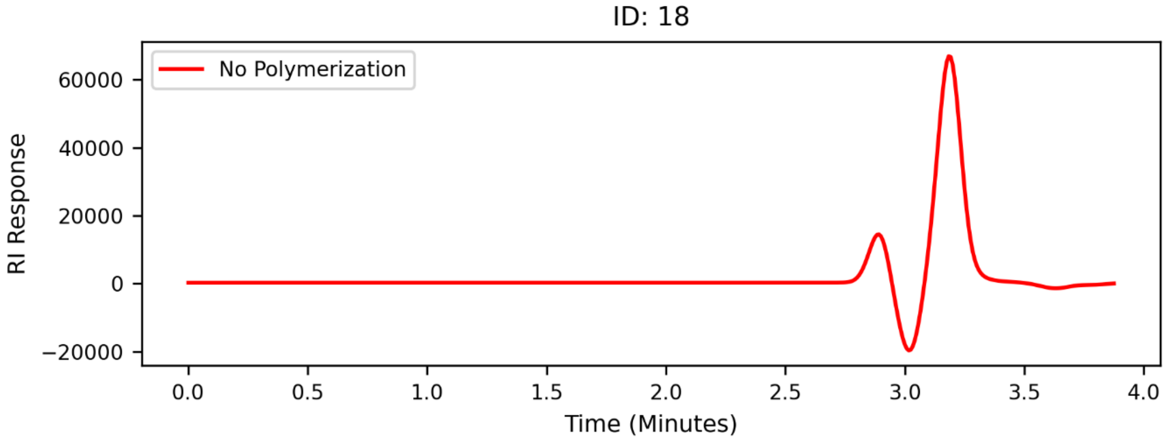
ID: 18
No Polymerization
RI Response
60000
40000
20000
0
−20000
0.0
0.5
1.0
1.5
2.0
2.5
3.0
3.5
4.0
Time (Minutes)

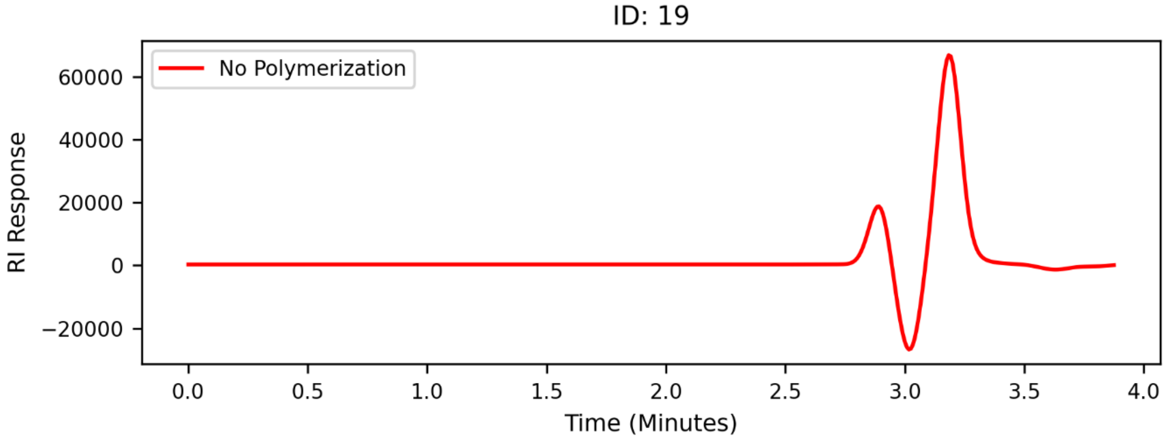
ID: 19
No Polymerization
RI Response
60000
40000
20000
0
−20000
0.0
0.5
1.0
1.5
2.0
2.5
3.0
3.5
4.0
Time (Minutes)

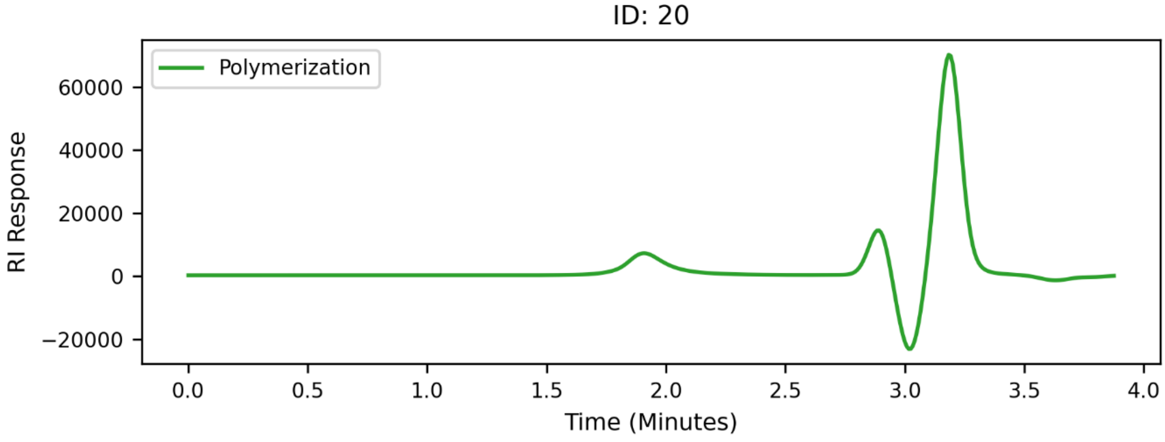
ID: 20
Polymerization
RI Response
60000
40000
20000
0
−20000
0.0
0.5
1.0
1.5
2.0
2.5
3.0
3.5
4.0
Time (Minutes)

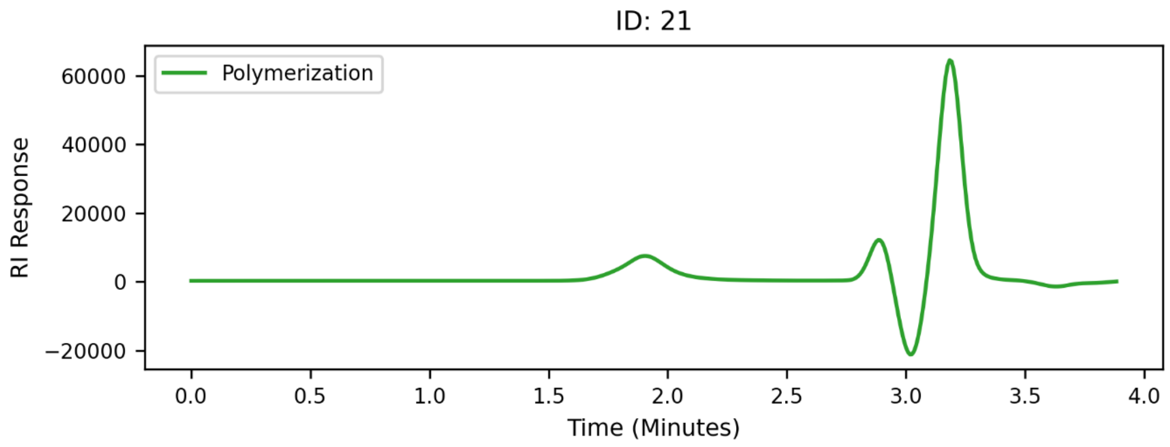
ID: 21
Polymerization
RI Response
Time (Minutes)
60000
40000
20000
0
−20000
0.0
0.5
1.0
1.5
2.0
2.5
3.0
3.5
4.0

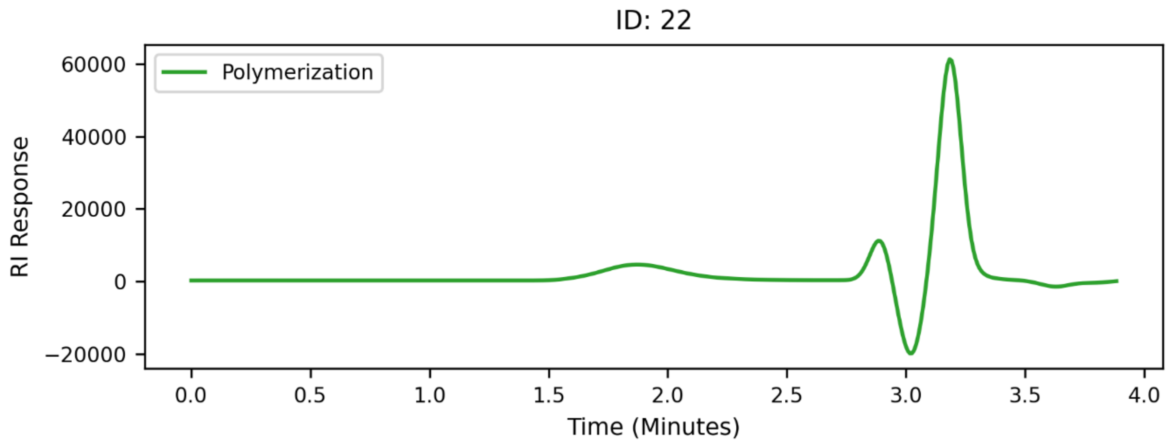
ID: 22
Polymerization
RI Response
Time (Minutes)
60000
40000
20000
0
−20000
0.0
0.5
1.0
1.5
2.0
2.5
3.0
3.5
4.0

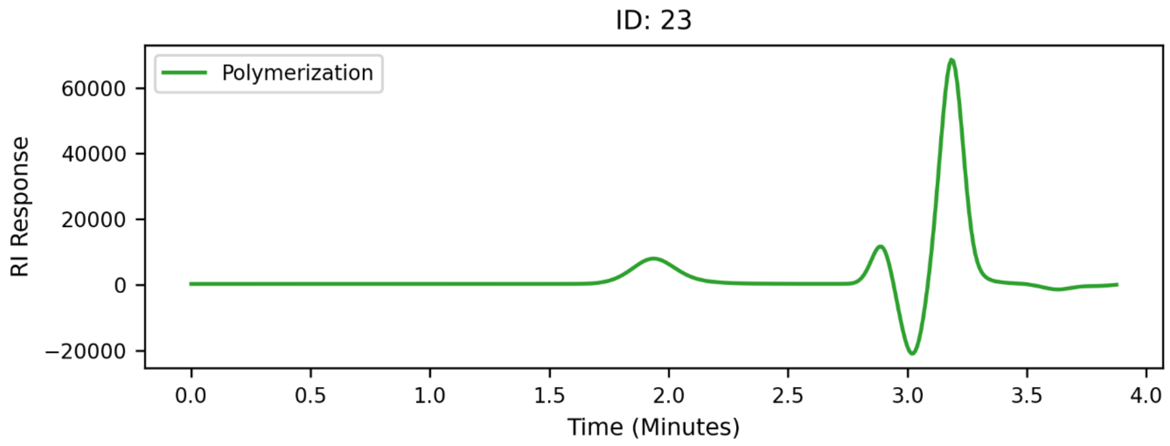
ID: 23
Polymerization
RI Response
Time (Minutes)
60000
40000
20000
0
−20000
0.0
0.5
1.0
1.5
2.0
2.5
3.0
3.5
4.0

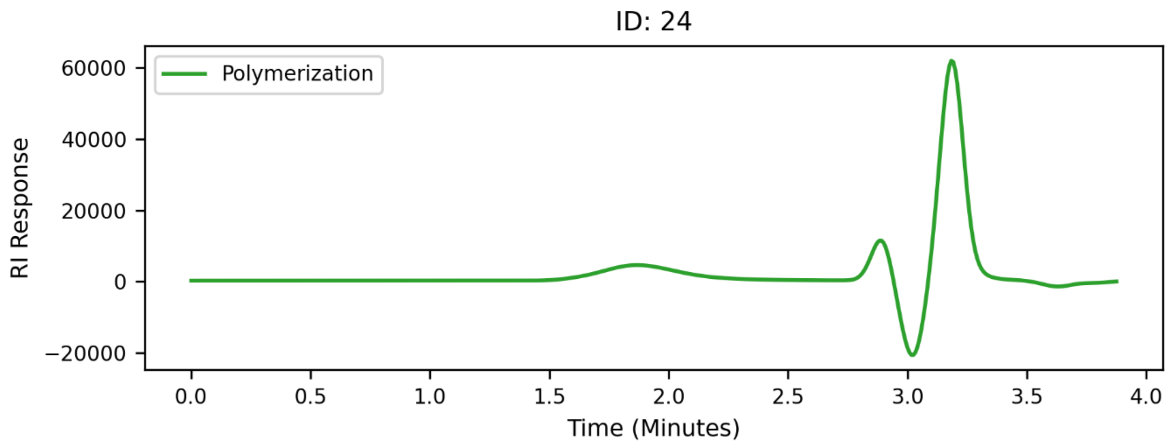
ID: 24
Polymerization
RI Response
Time (Minutes)
60000
40000
20000
0
−20000
0.0
0.5
1.0
1.5
2.0
2.5
3.0
3.5
4.0

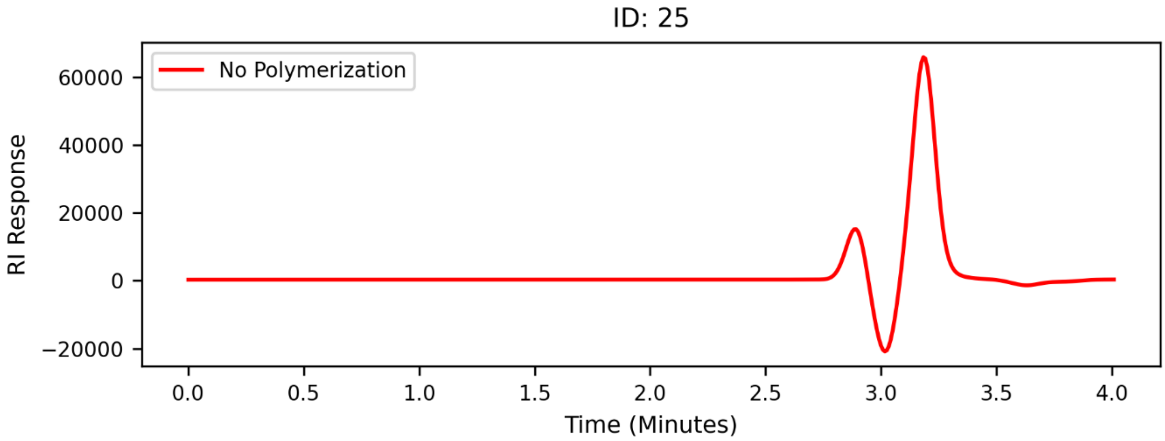
ID: 25
No Polymerization
RI Response
60000
40000
20000
0
−20000
0.0
0.5
1.0
1.5
2.0
2.5
3.0
3.5
4.0
Time (Minutes)

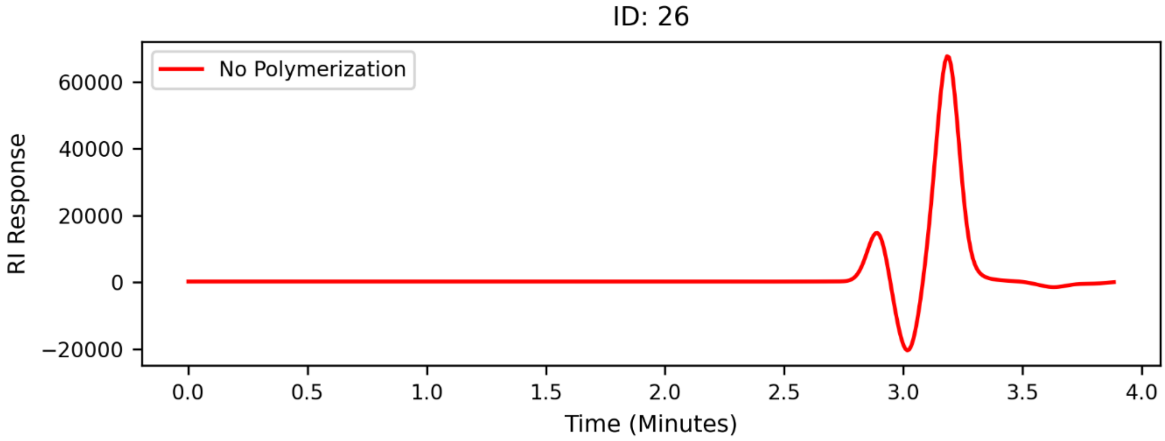
ID: 26
No Polymerization
RI Response
60000
40000
20000
0
−20000
0.0
0.5
1.0
1.5
2.0
2.5
3.0
3.5
4.0
Time (Minutes)

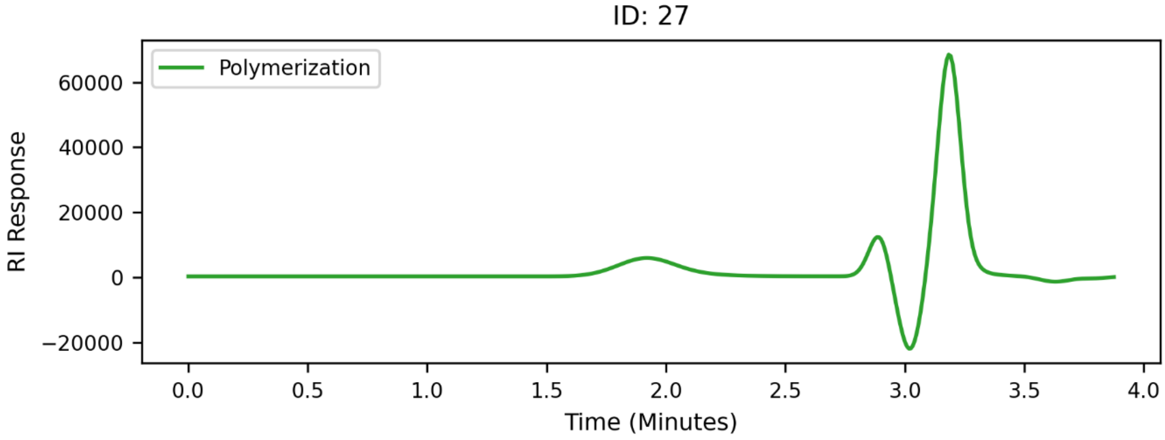
ID: 27
Polymerization
RI Response
60000
40000
20000
0
−20000
0.0
0.5
1.0
1.5
2.0
2.5
3.0
3.5
4.0
Time (Minutes)

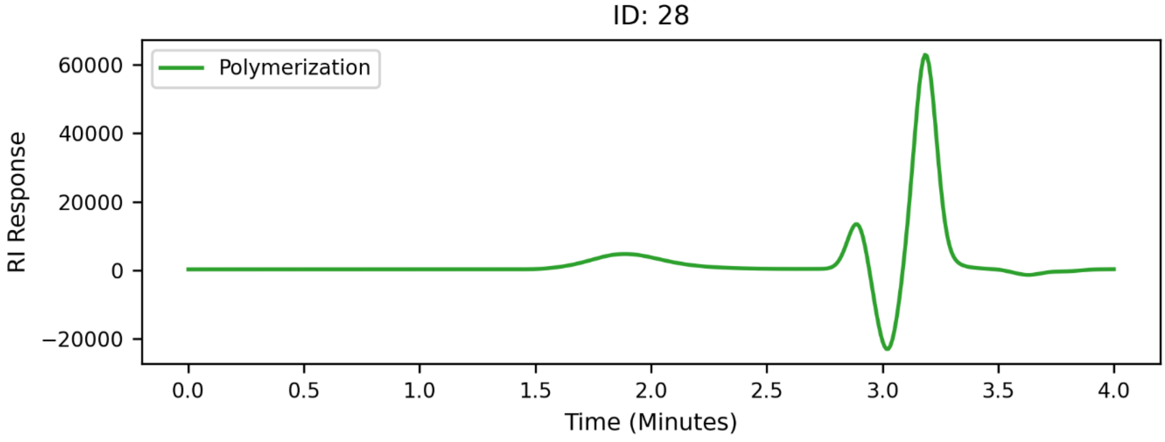
ID: 28
Polymerization
RI Response
60000
40000
20000
0
−20000
0.0
0.5
1.0
1.5
2.0
2.5
3.0
3.5
4.0
Time (Minutes)

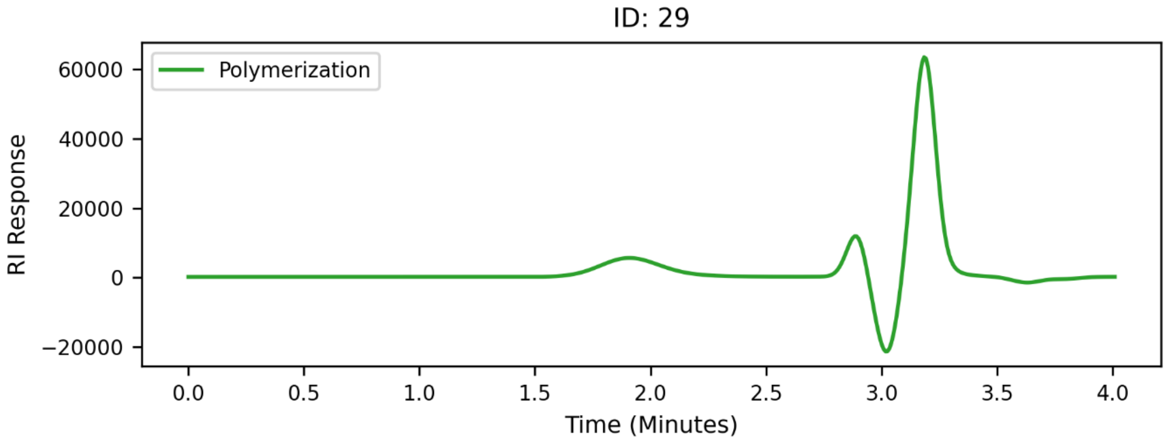
ID: 29
Polymerization
60000
40000
20000
0
−20000
0.0
0.5
1.0
1.5
2.0
2.5
3.0
3.5
4.0
Time (Minutes)
RI Response

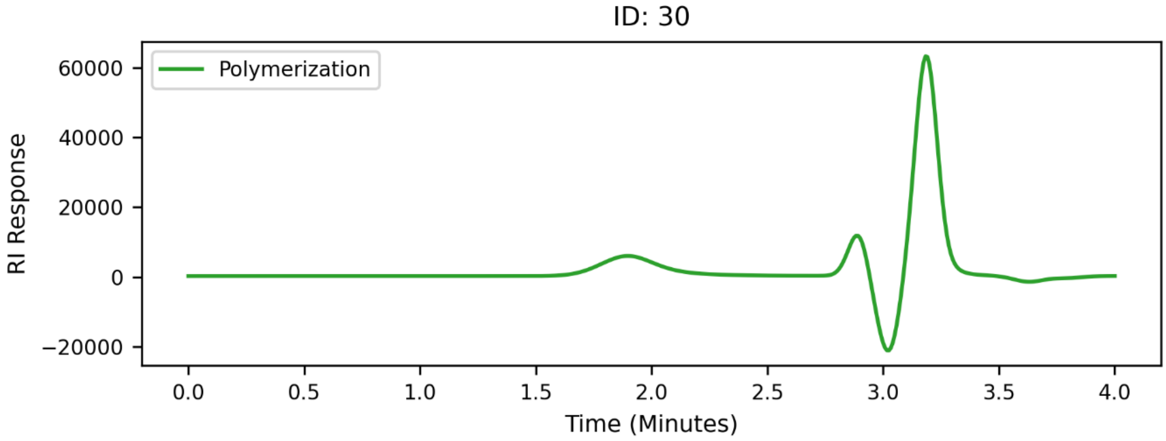
ID: 30
Polymerization
60000
40000
20000
0
−20000
0.0
0.5
1.0
1.5
2.0
2.5
3.0
3.5
4.0
Time (Minutes)
RI Response

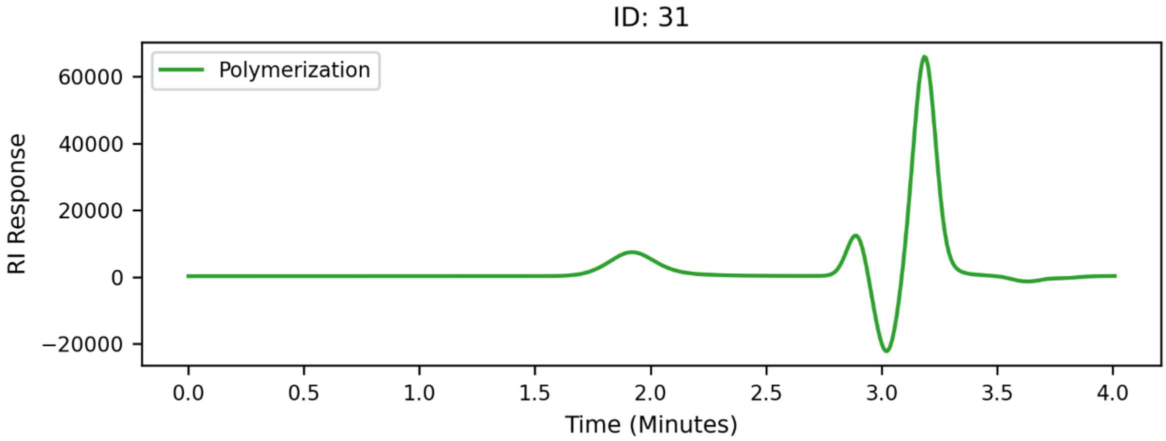
ID: 31
Polymerization
60000
40000
20000
0
−20000
0.0
0.5
1.0
1.5
2.0
2.5
3.0
3.5
4.0
Time (Minutes)
RI Response

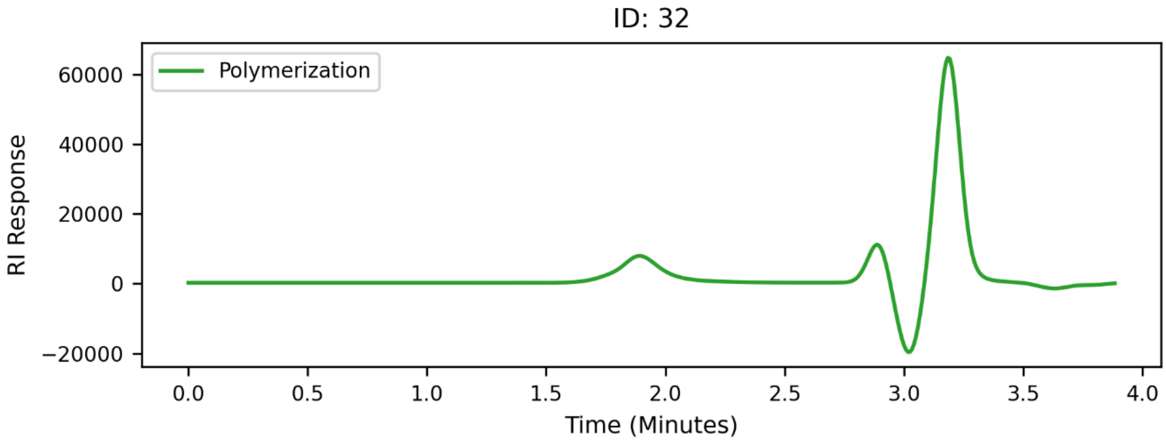
ID: 32
Polymerization
60000
40000
20000
0
−20000
0.0
0.5
1.0
1.5
2.0
2.5
3.0
3.5
4.0
Time (Minutes)
RI Response

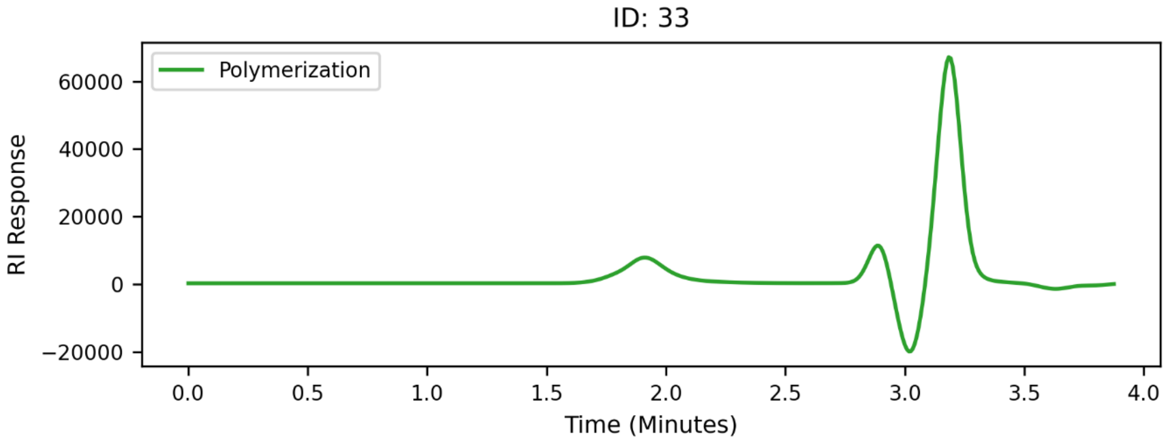
ID: 33
Polymerization
RI Response
60000
40000
20000
0
−20000
0.0
0.5
1.0
1.5
2.0
2.5
3.0
3.5
4.0
Time (Minutes)

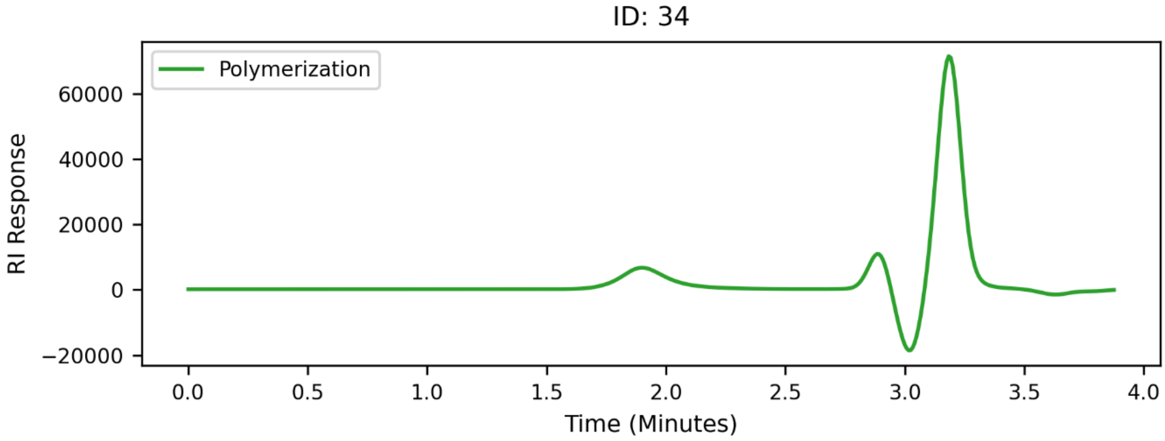
ID: 34
Polymerization
RI Response
60000
40000
20000
0
−20000
0.0
0.5
1.0
1.5
2.0
2.5
3.0
3.5
4.0
Time (Minutes)

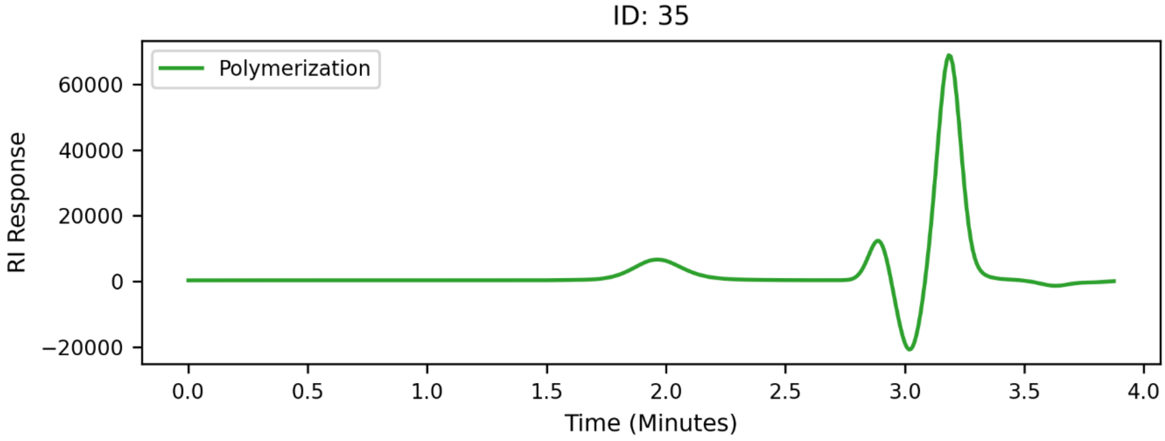
ID: 35
Polymerization
RI Response
60000
40000
20000
0
−20000
0.0
0.5
1.0
1.5
2.0
2.5
3.0
3.5
4.0
Time (Minutes)

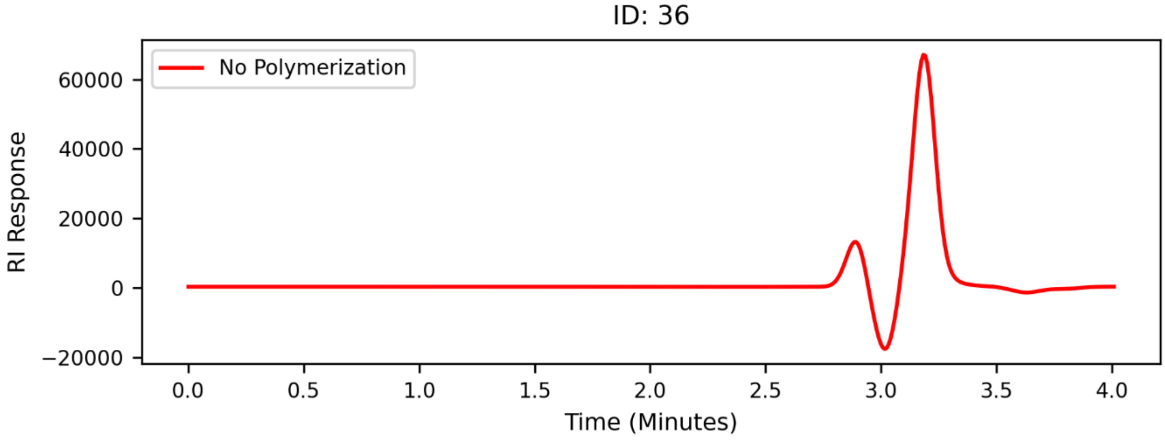
ID: 36
No Polymerization
RI Response
60000
40000
20000
0
−20000
0.0
0.5
1.0
1.5
2.0
2.5
3.0
3.5
4.0
Time (Minutes)

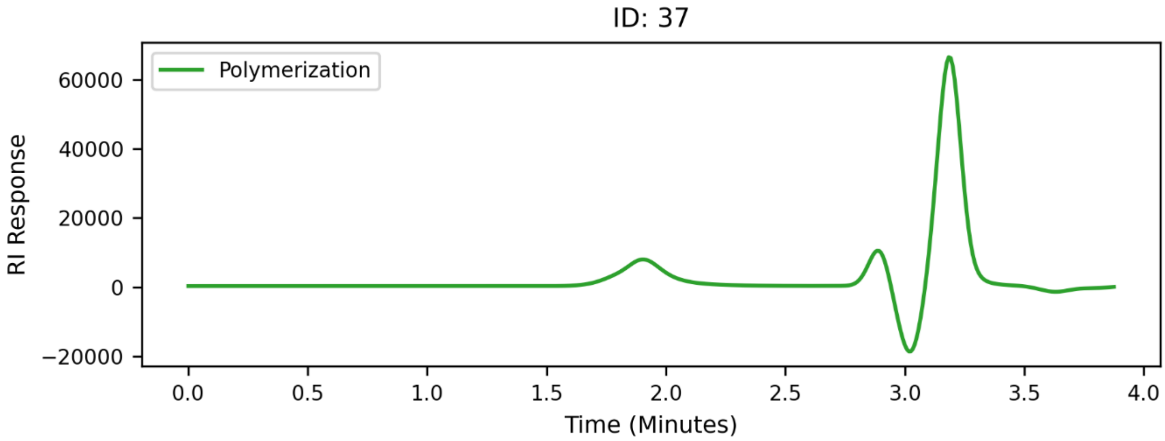
ID: 37
Polymerization
RI Response
60000
40000
20000
0
−20000
0.0
0.5
1.0
1.5
2.0
2.5
3.0
3.5
4.0
Time (Minutes)

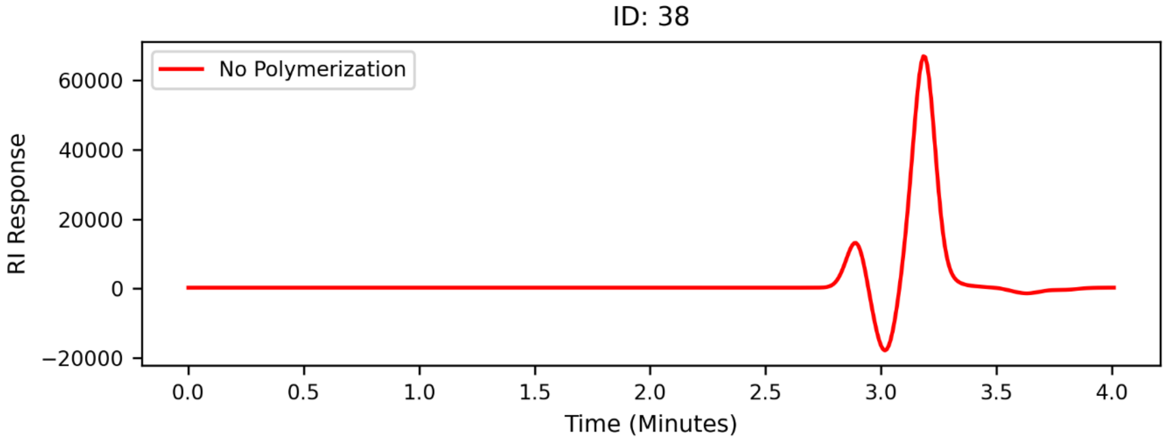
ID: 38
No Polymerization
RI Response
60000
40000
20000
0
−20000
0.0
0.5
1.0
1.5
2.0
2.5
3.0
3.5
4.0
Time (Minutes)

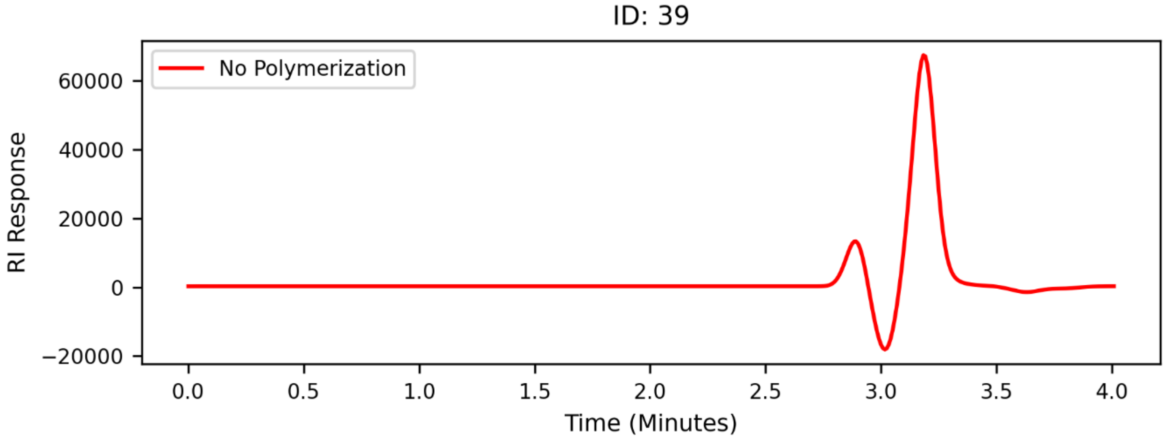
ID: 39
No Polymerization
RI Response
60000
40000
20000
0
−20000
0.0
0.5
1.0
1.5
2.0
2.5
3.0
3.5
4.0
Time (Minutes)

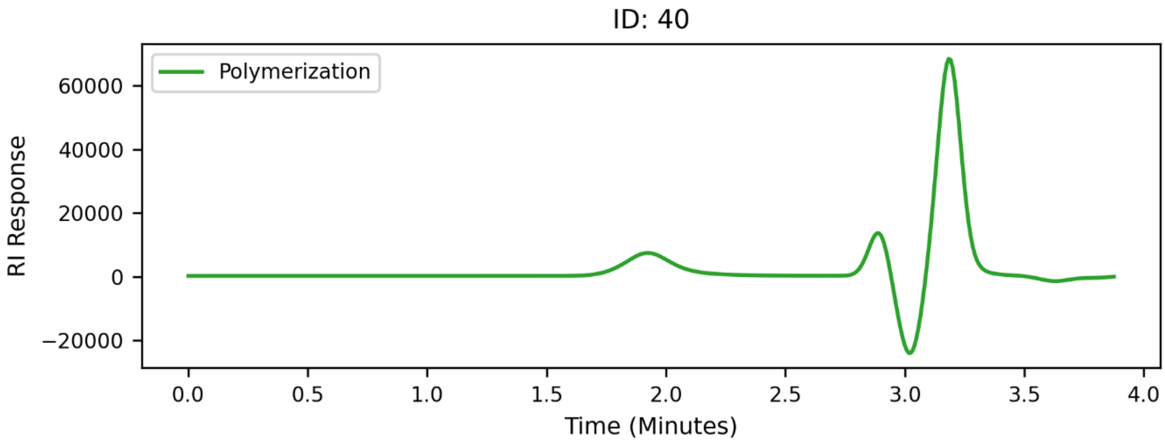
ID: 40
Polymerization
RI Response
60000
40000
20000
0
−20000
0.0
0.5
1.0
1.5
2.0
2.5
3.0
3.5
4.0
Time (Minutes)

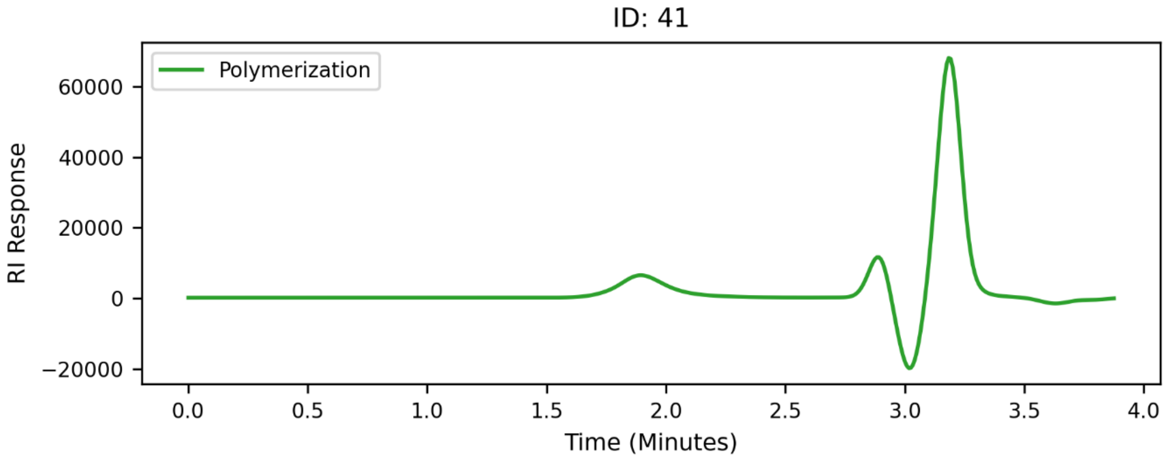
ID: 41
Polymerization
RI Response
60000
40000
20000
0
−20000
0.0
0.5
1.0
1.5
2.0
2.5
3.0
3.5
4.0
Time (Minutes)

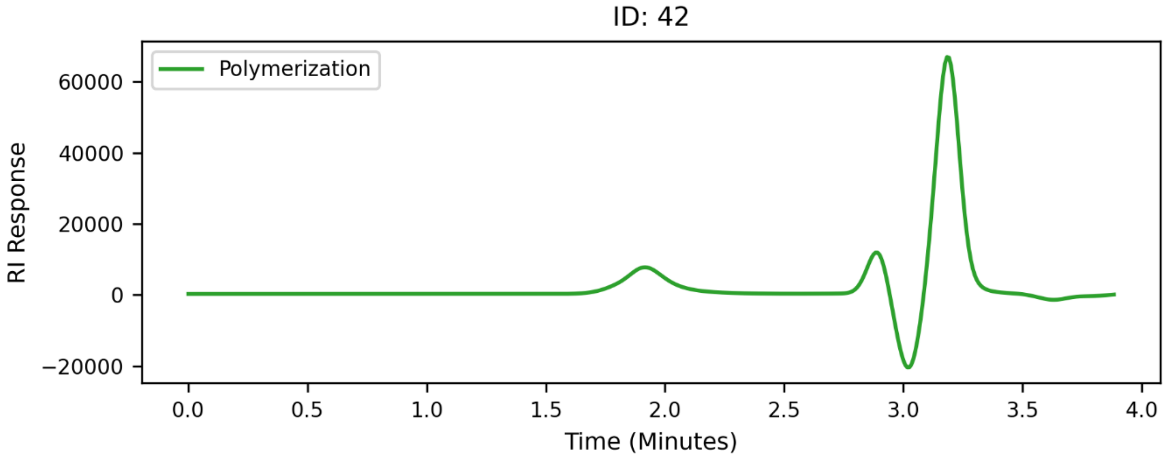
ID: 42
Polymerization
RI Response
60000
40000
20000
0
−20000
0.0
0.5
1.0
1.5
2.0
2.5
3.0
3.5
4.0
Time (Minutes)

Appendix 1B: SEC Traces for Validation/ML Data (Batch 2)

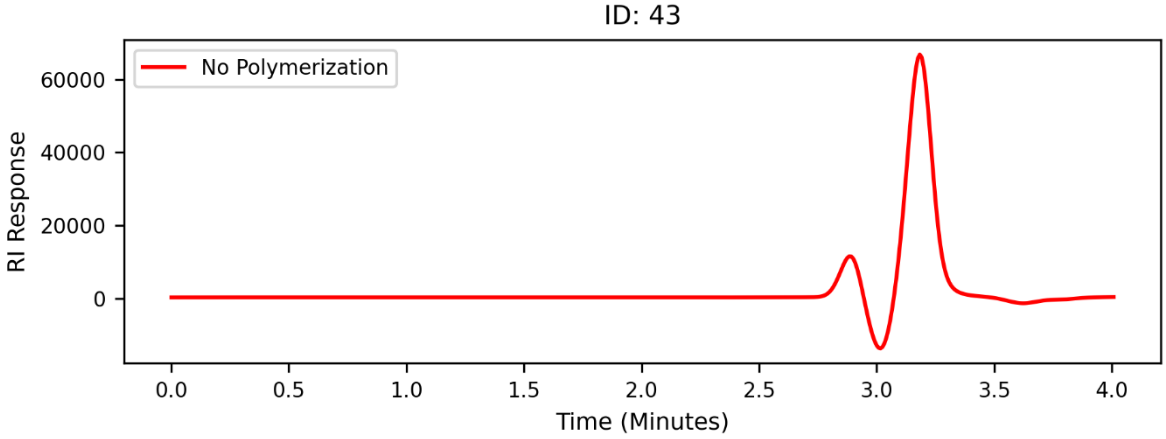

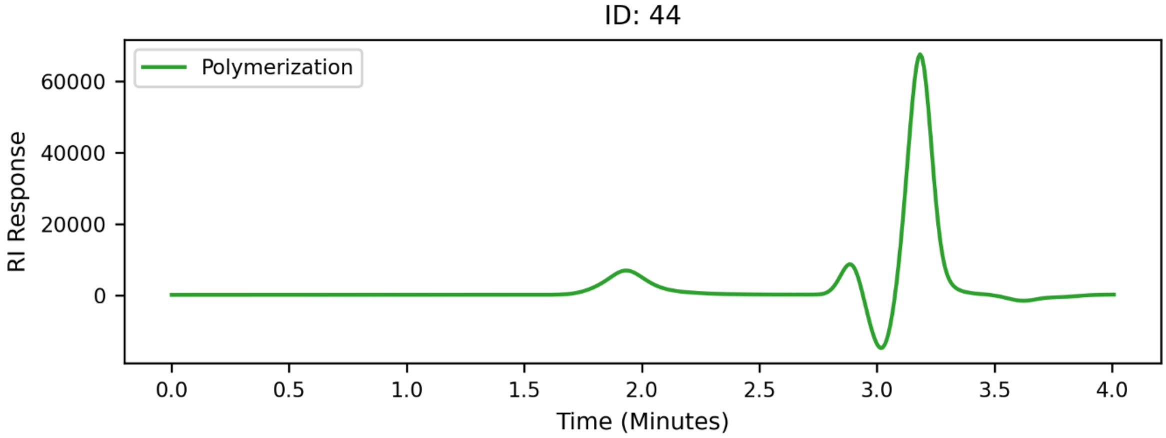

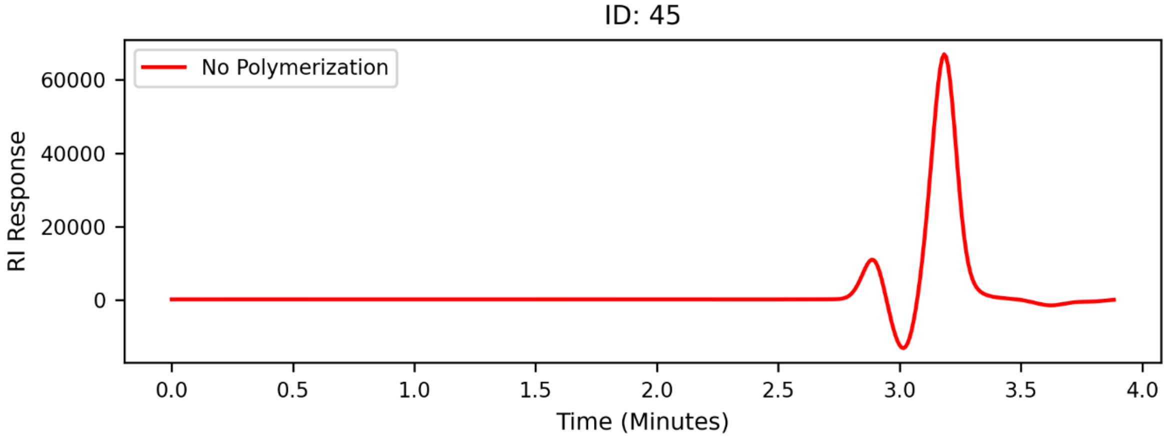

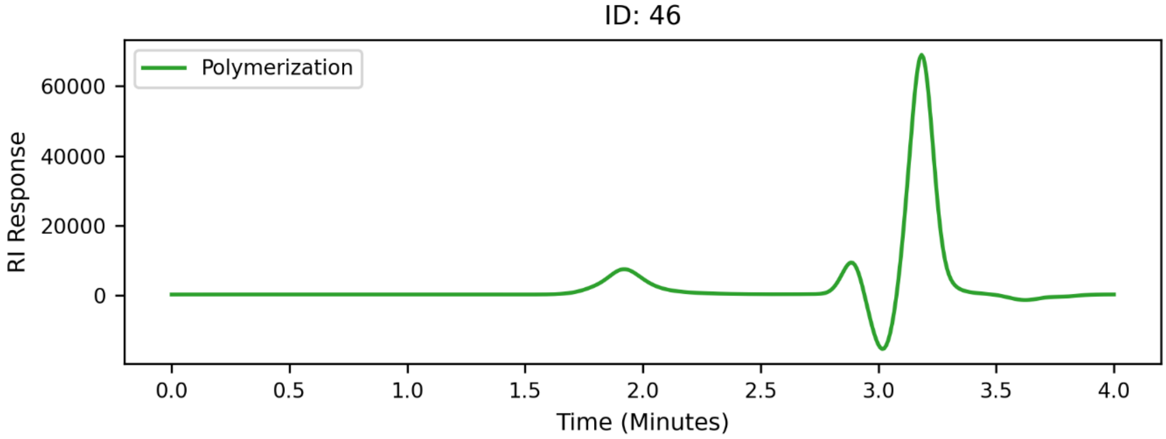

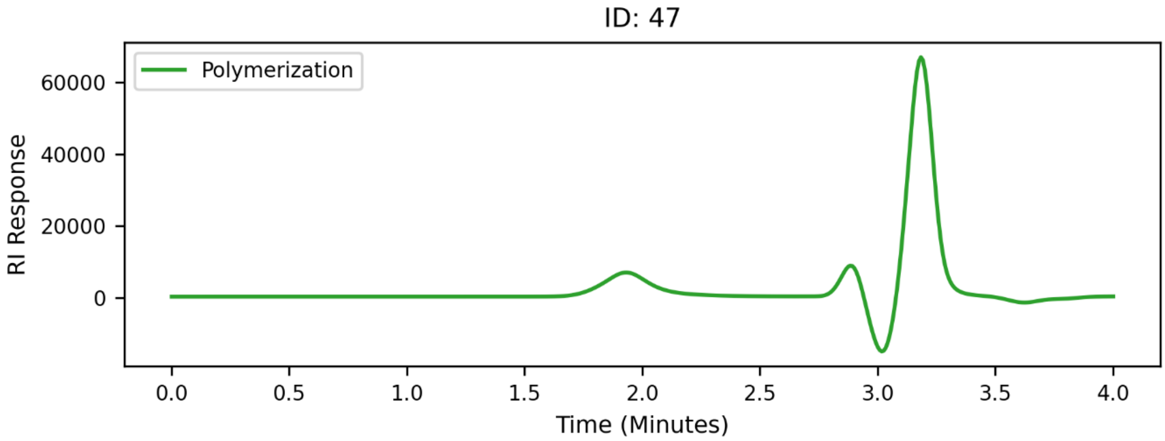
ID: 47
Polymerization
RI Response
Time (Minutes)

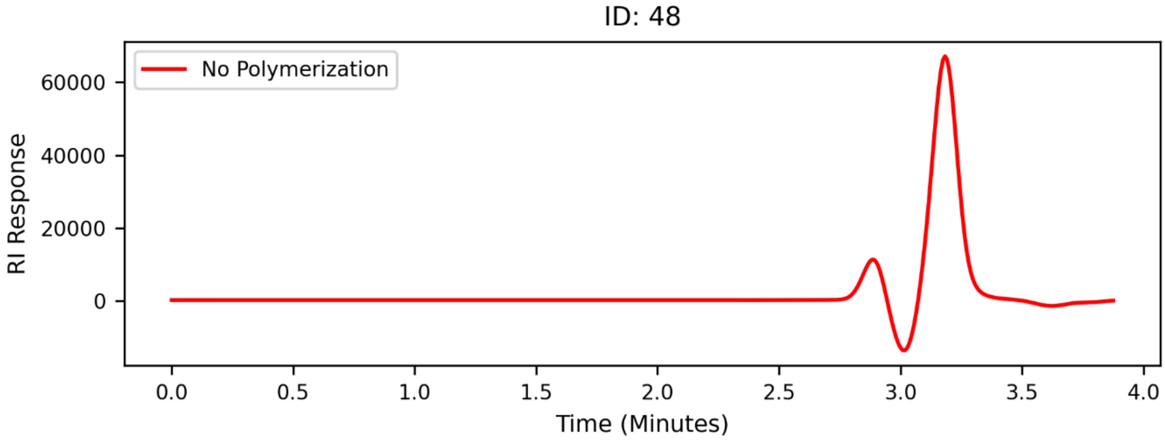
ID: 48
No Polymerization
RI Response
Time (Minutes)

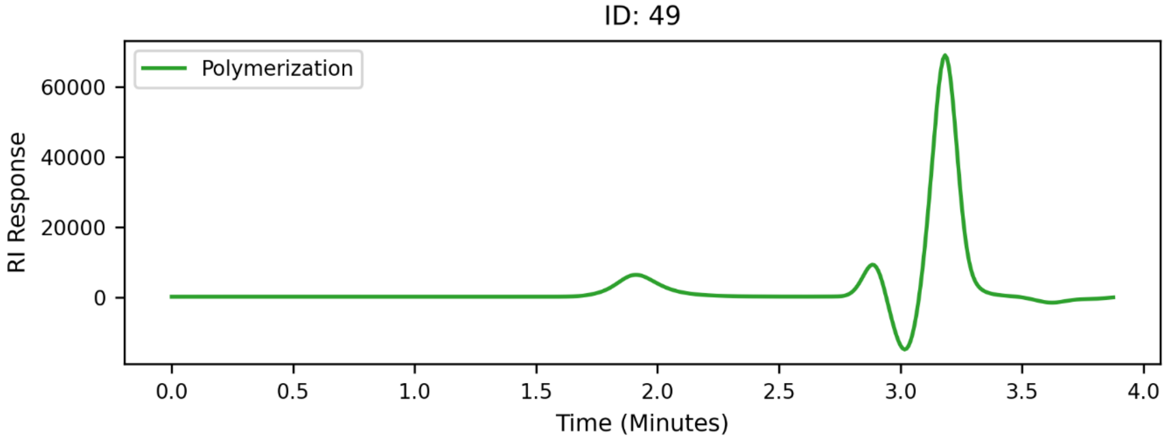
ID: 49
Polymerization
RI Response
Time (Minutes)

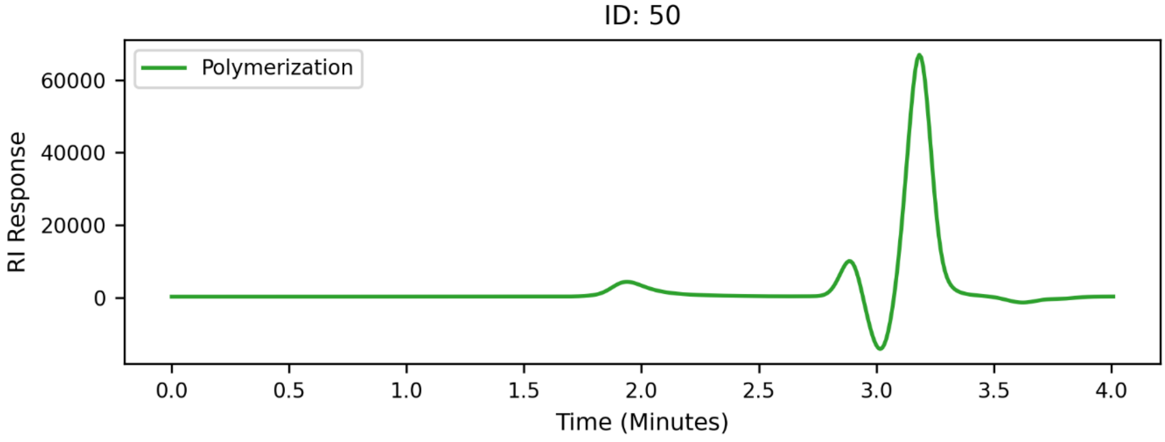
ID: 50
Polymerization
RI Response
Time (Minutes)

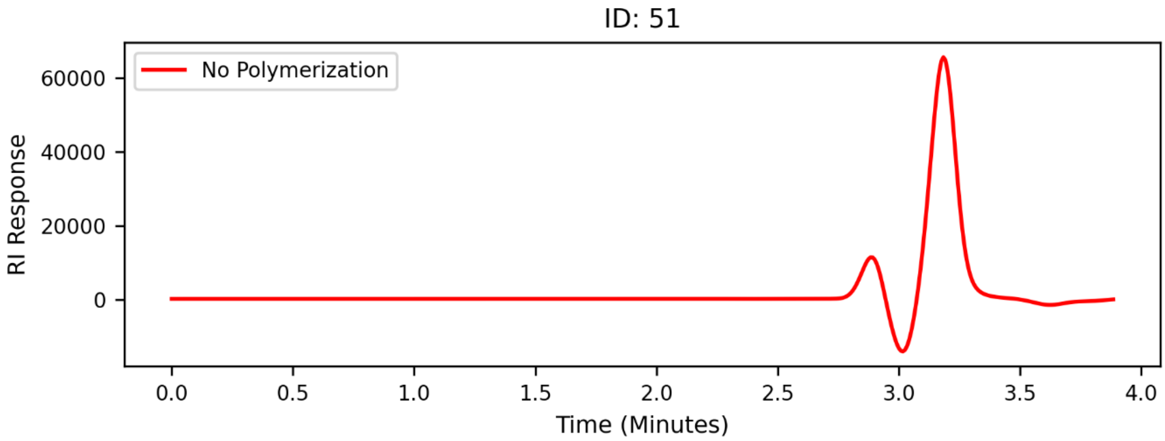
ID: 51
No Polymerization
RI Response
60000
40000
20000
0
0.0
0.5
1.0
1.5
2.0
2.5
3.0
3.5
4.0
Time (Minutes)

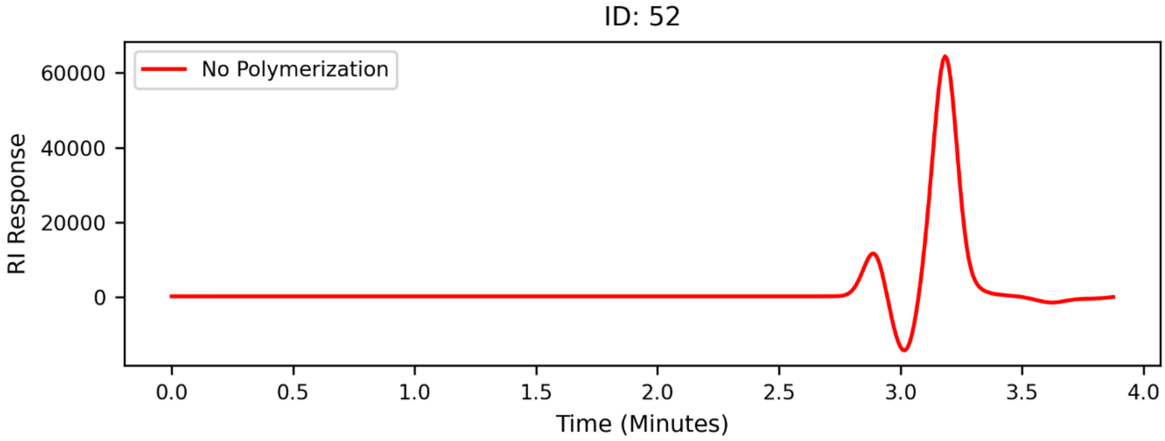
ID: 52
No Polymerization
RI Response
60000
40000
20000
0
0.0
0.5
1.0
1.5
2.0
2.5
3.0
3.5
4.0
Time (Minutes)

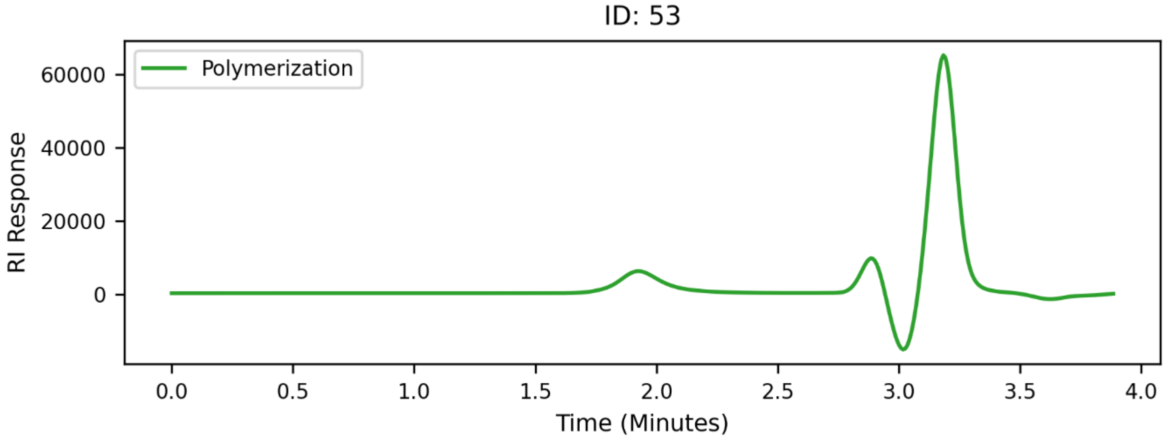
ID: 53
Polymerization
RI Response
60000
40000
20000
0
0.0
0.5
1.0
1.5
2.0
2.5
3.0
3.5
4.0
Time (Minutes)

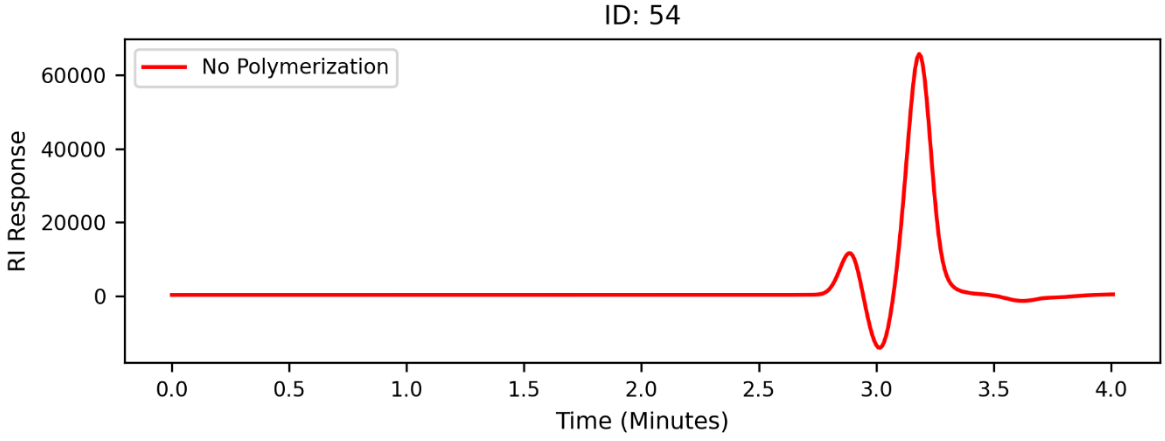
ID: 54
No Polymerization
RI Response
60000
40000
20000
0
0.0
0.5
1.0
1.5
2.0
2.5
3.0
3.5
4.0
Time (Minutes)

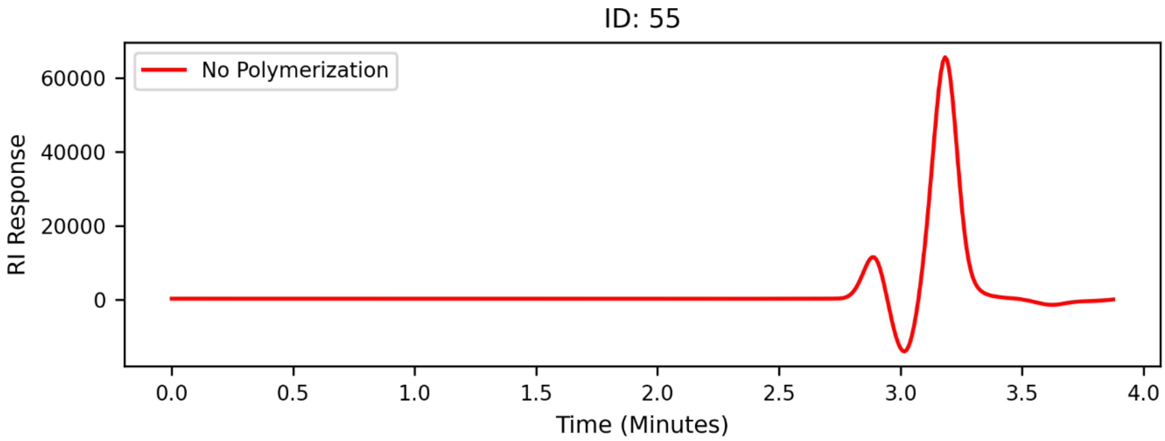
ID: 55
No Polymerization
RI Response
60000
40000
20000
0
0.0
0.5
1.0
1.5
2.0
2.5
3.0
3.5
4.0
Time (Minutes)

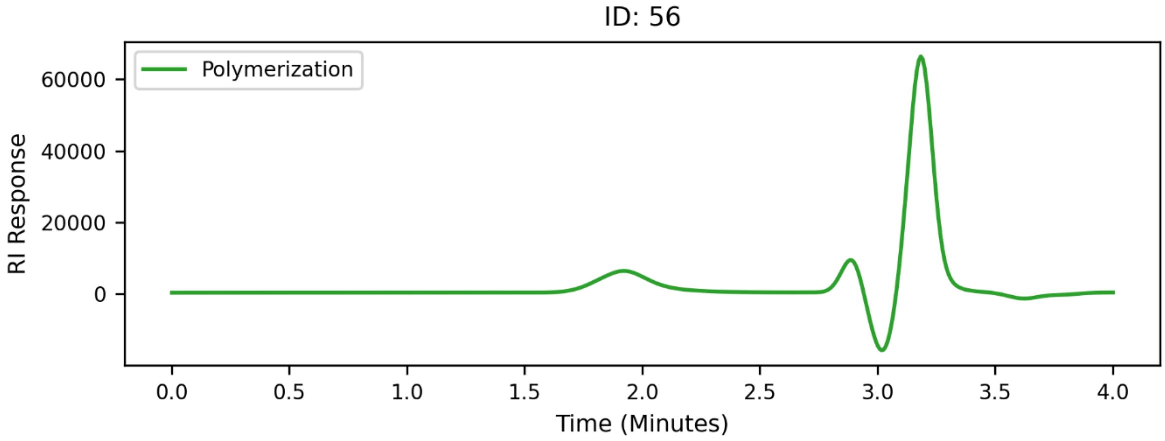
ID: 56
Polymerization
RI Response
60000
40000
20000
0
0.0
0.5
1.0
1.5
2.0
2.5
3.0
3.5
4.0
Time (Minutes)

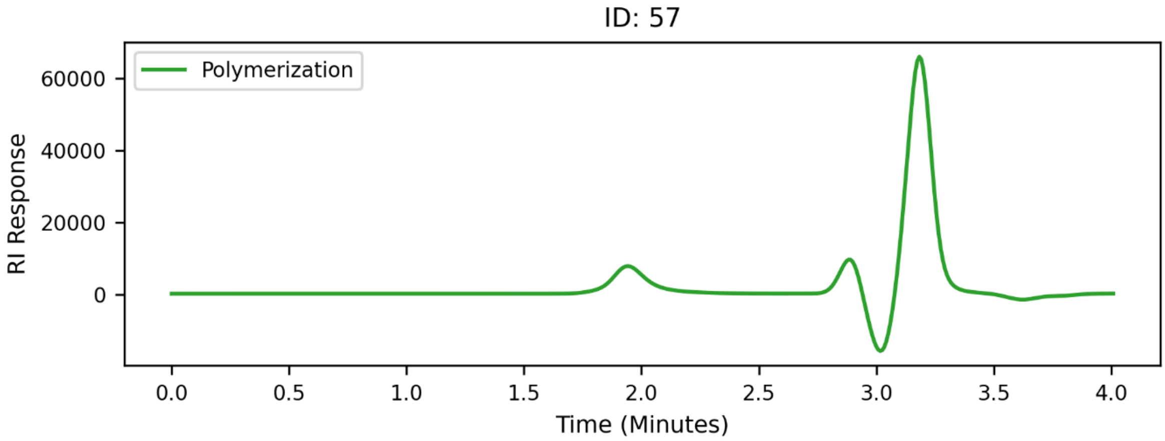
ID: 57
Polymerization
RI Response
60000
40000
20000
0
0.0
0.5
1.0
1.5
2.0
2.5
3.0
3.5
4.0
Time (Minutes)

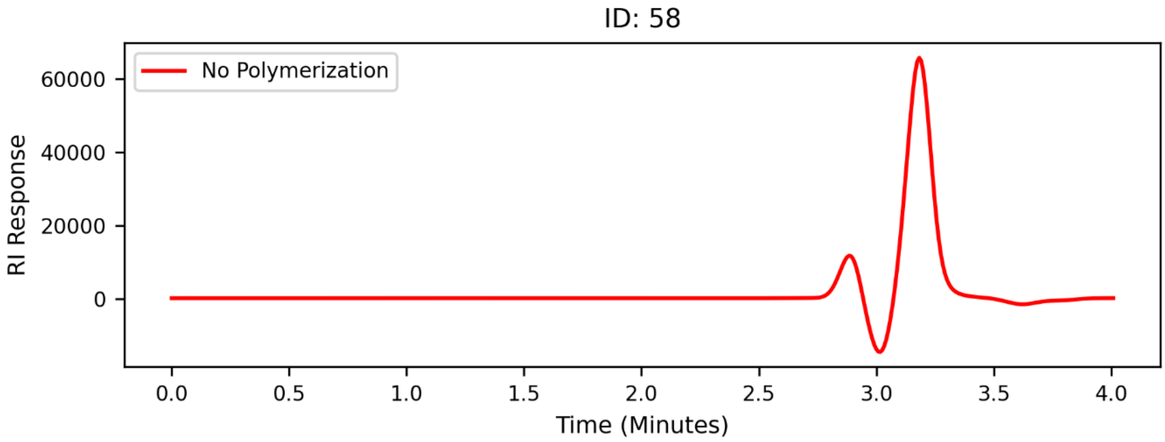
ID: 58
No Polymerization
RI Response
60000
40000
20000
0
0.0
0.5
1.0
1.5
2.0
2.5
3.0
3.5
4.0
Time (Minutes)

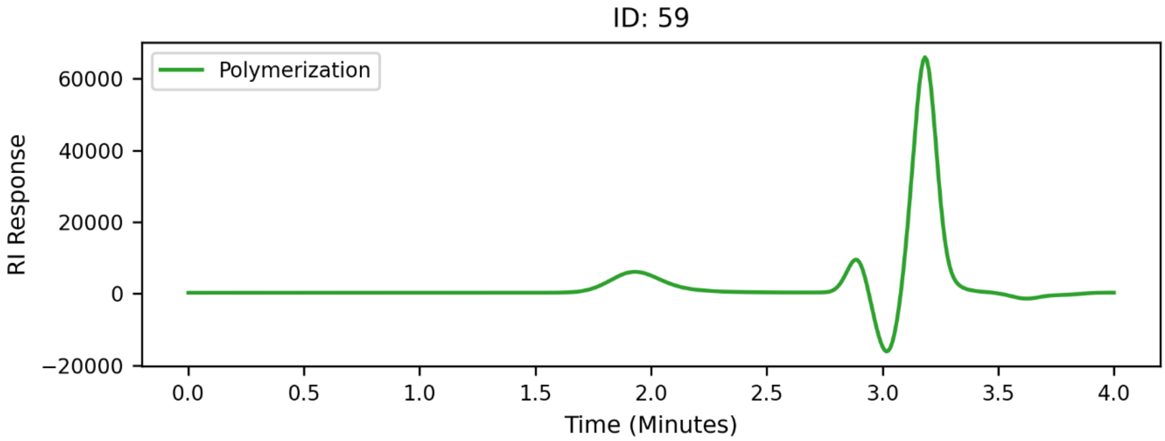
ID: 59
Polymerization
RI Response
60000
40000
20000
0
−20000
0.0
0.5
1.0
1.5
2.0
2.5
3.0
3.5
4.0
Time (Minutes)

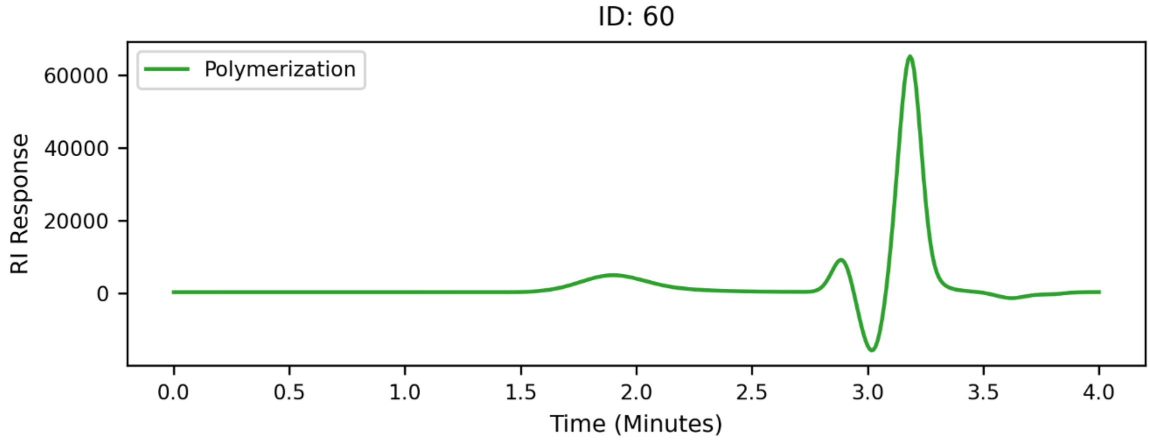
ID: 60
Polymerization
RI Response
60000
40000
20000
0
0.0
0.5
1.0
1.5
2.0
2.5
3.0
3.5
4.0
Time (Minutes)

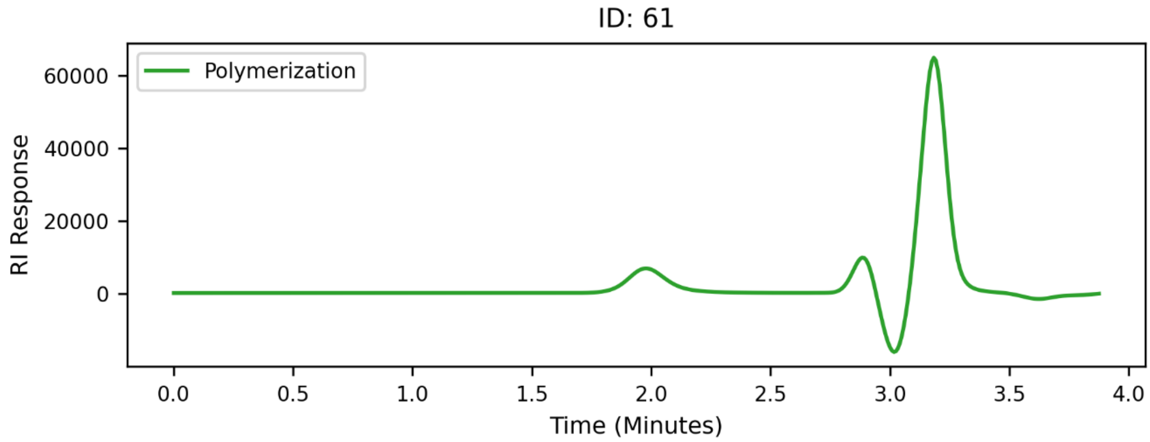
ID: 61
Polymerization
RI Response
60000
40000
20000
0
0.0
0.5
1.0
1.5
2.0
2.5
3.0
3.5
4.0
Time (Minutes)

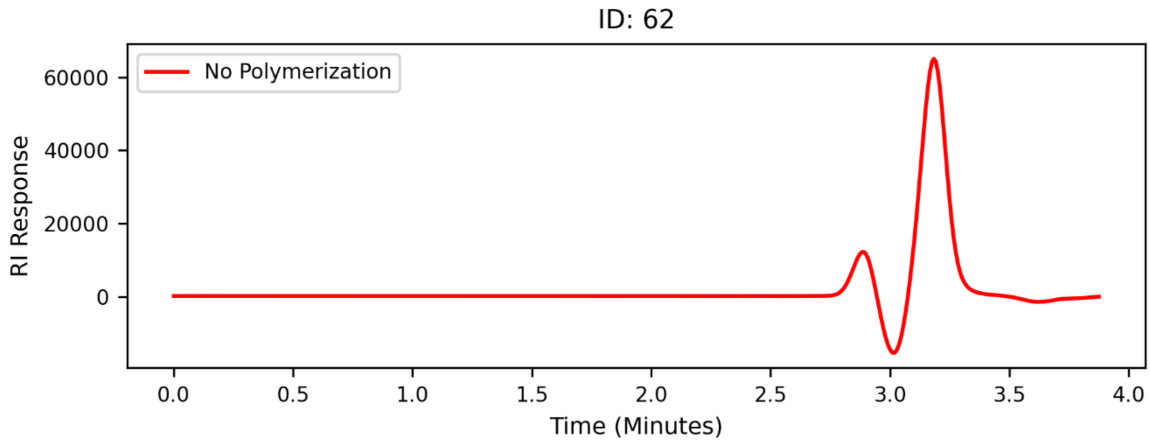
ID: 62
No Polymerization
RI Response
60000
40000
20000
0
0.0
0.5
1.0
1.5
2.0
2.5
3.0
3.5
4.0
Time (Minutes)

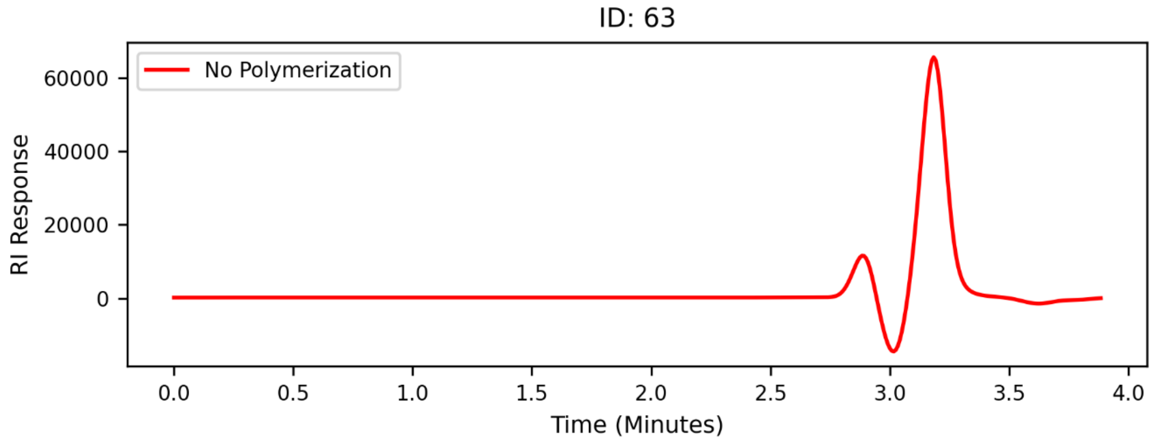
ID: 63
No Polymerization
RI Response
60000
40000
20000
0
0.0
0.5
1.0
1.5
2.0
2.5
3.0
3.5
4.0
Time (Minutes)

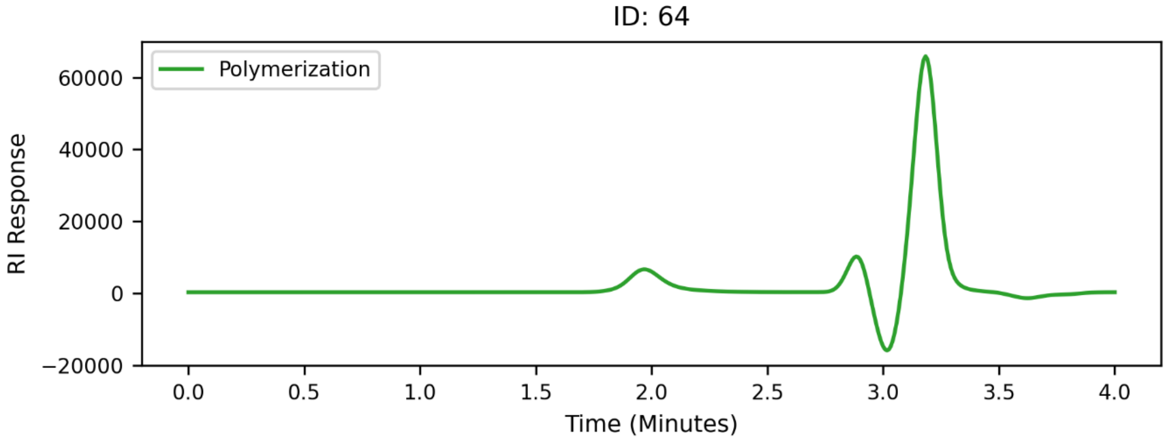
ID: 64
Polymerization
RI Response
60000
40000
20000
0
−20000
0.0
0.5
1.0
1.5
2.0
2.5
3.0
3.5
4.0
Time (Minutes)

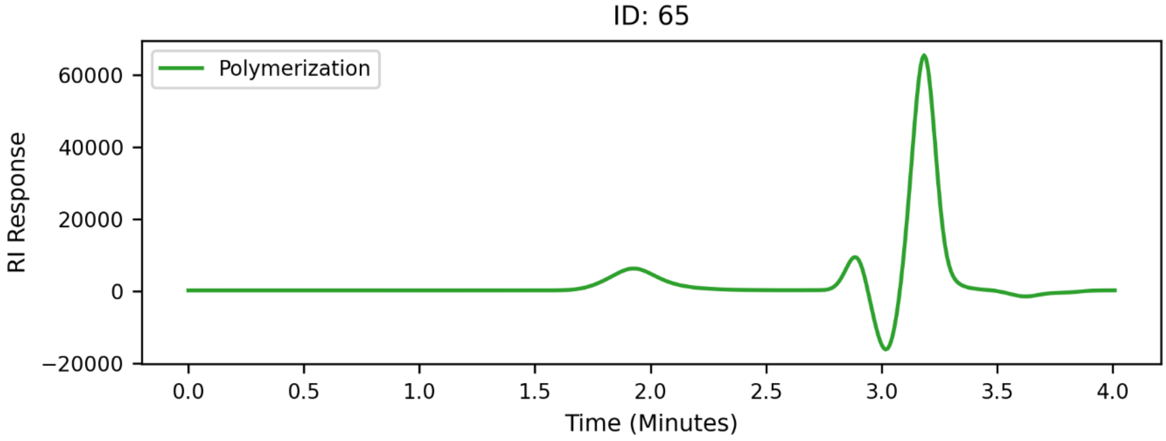
ID: 65
Polymerization
RI Response
60000
40000
20000
0
−20000
0.0
0.5
1.0
1.5
2.0
2.5
3.0
3.5
4.0
Time (Minutes)

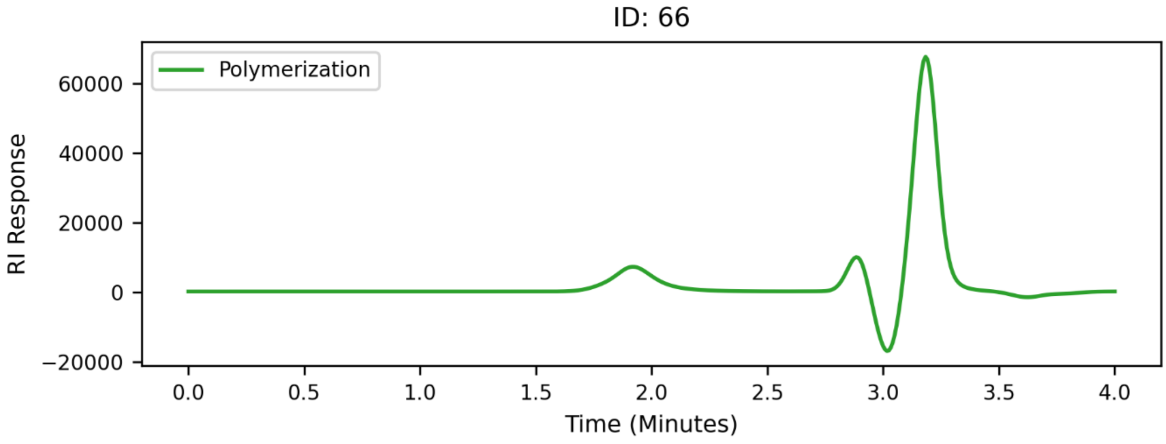
ID: 66
Polymerization
RI Response
60000
40000
20000
0
−20000
0.0
0.5
1.0
1.5
2.0
2.5
3.0
3.5
4.0
Time (Minutes)

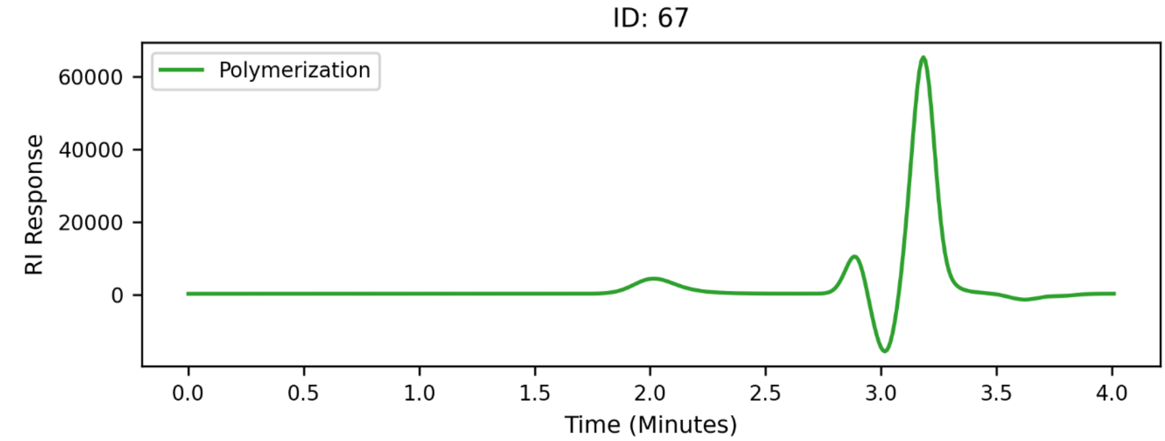
ID: 67
Polymerization
RI Response
Time (Minutes)

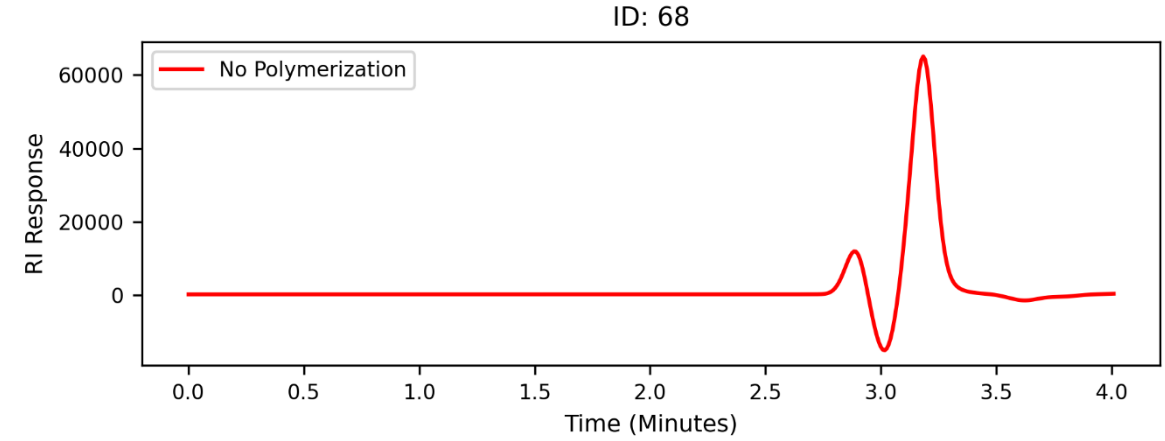
ID: 68
No Polymerization
RI Response
Time (Minutes)

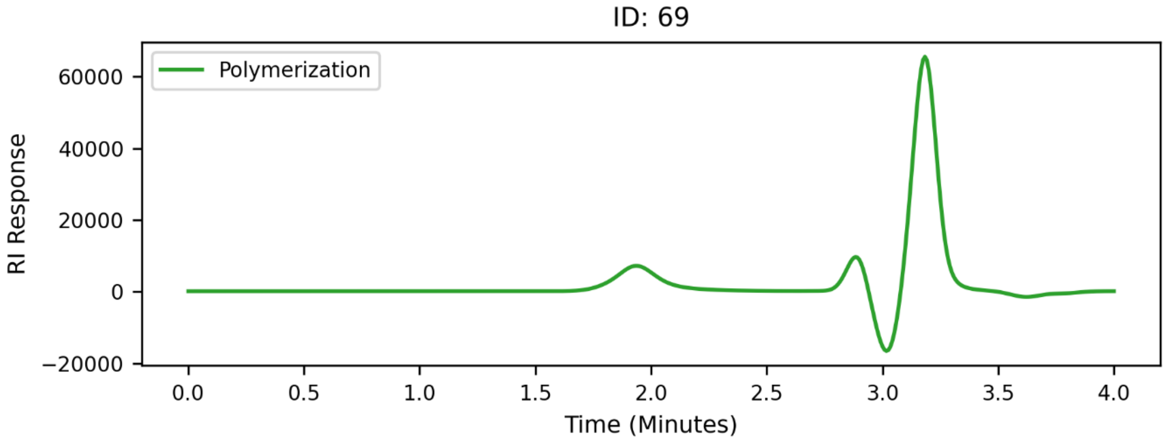
ID: 69
Polymerization
RI Response
Time (Minutes)

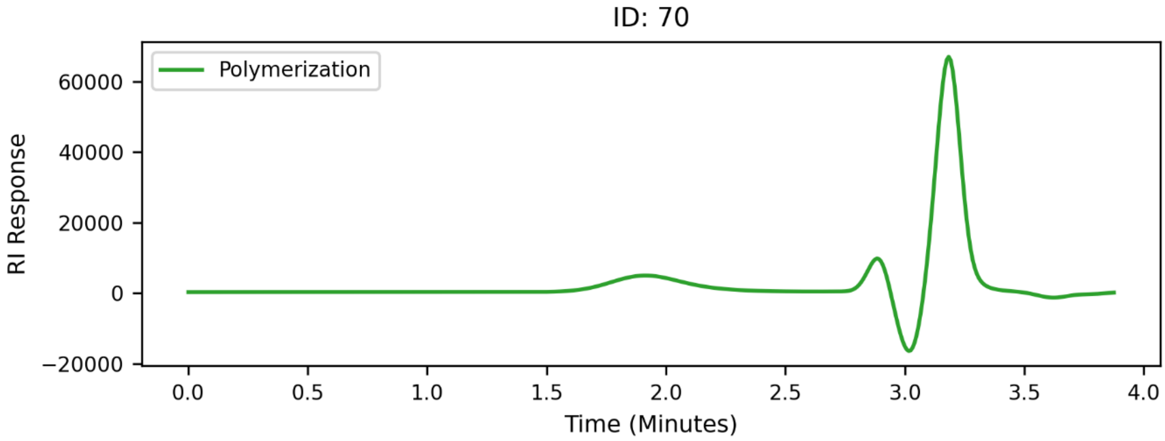
ID: 70
Polymerization
RI Response
Time (Minutes)

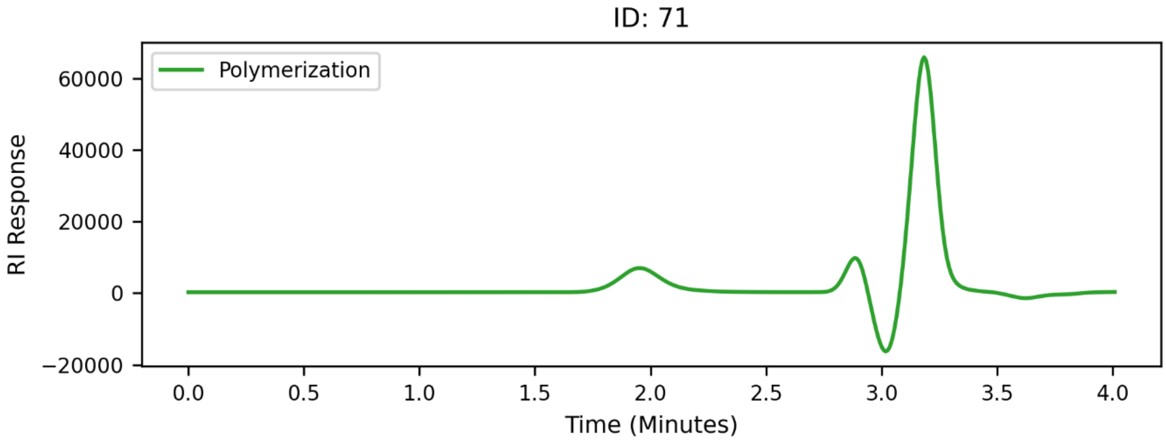
ID: 71
Polymerization
RI Response
60000
40000
20000
0
−20000
0.0
0.5
1.0
1.5
2.0
2.5
3.0
3.5
4.0
Time (Minutes)

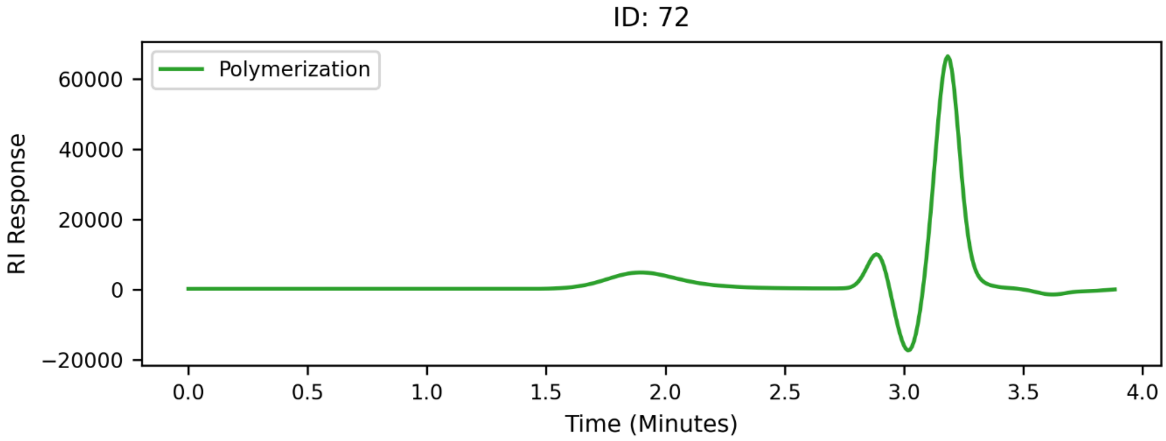
ID: 72
Polymerization
RI Response
60000
40000
20000
0
−20000
0.0
0.5
1.0
1.5
2.0
2.5
3.0
3.5
4.0
Time (Minutes)

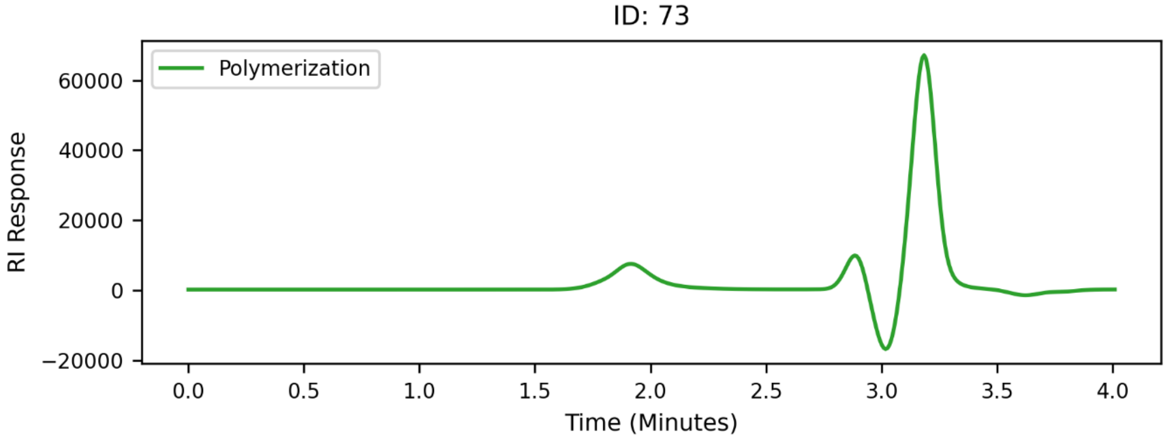
ID: 73
Polymerization
RI Response
60000
40000
20000
0
−20000
0.0
0.5
1.0
1.5
2.0
2.5
3.0
3.5
4.0
Time (Minutes)

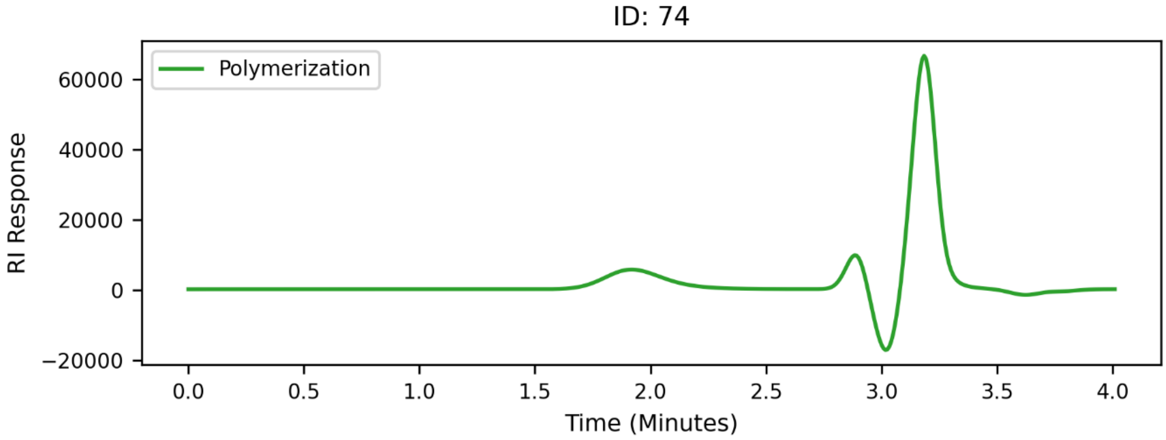
ID: 74
Polymerization
RI Response
60000
40000
20000
0
−20000
0.0
0.5
1.0
1.5
2.0
2.5
3.0
3.5
4.0
Time (Minutes)

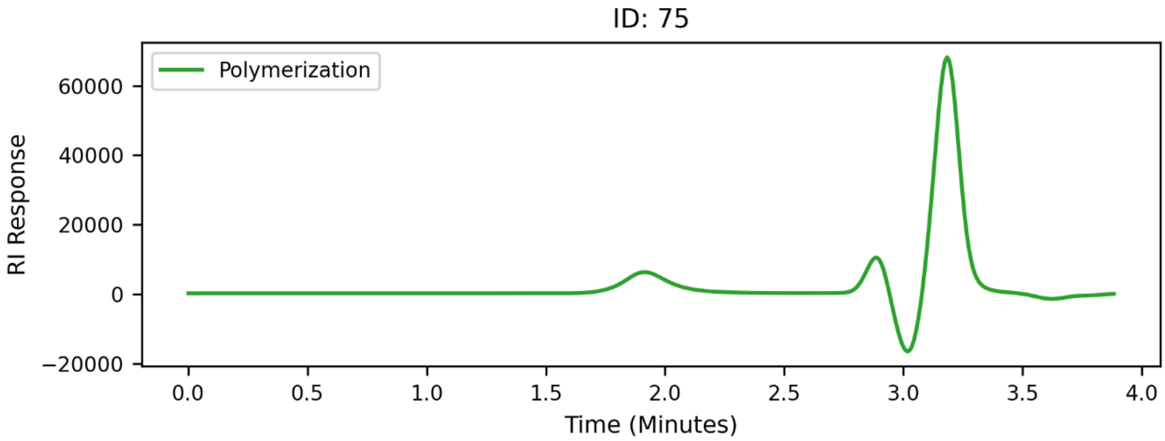
ID: 75
Polymerization
RI Response
Time (Minutes)
60000
40000
20000
0
−20000
0.0
0.5
1.0
1.5
2.0
2.5
3.0
3.5
4.0
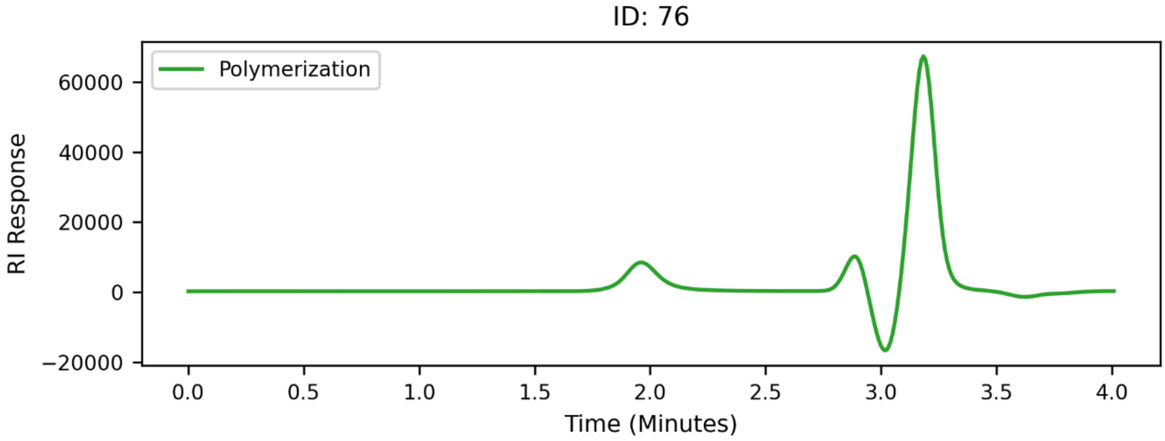
ID: 76
Polymerization
RI Response
Time (Minutes)
60000
40000
20000
0
−20000
0.0
0.5
1.0
1.5
2.0
2.5
3.0
3.5
4.0
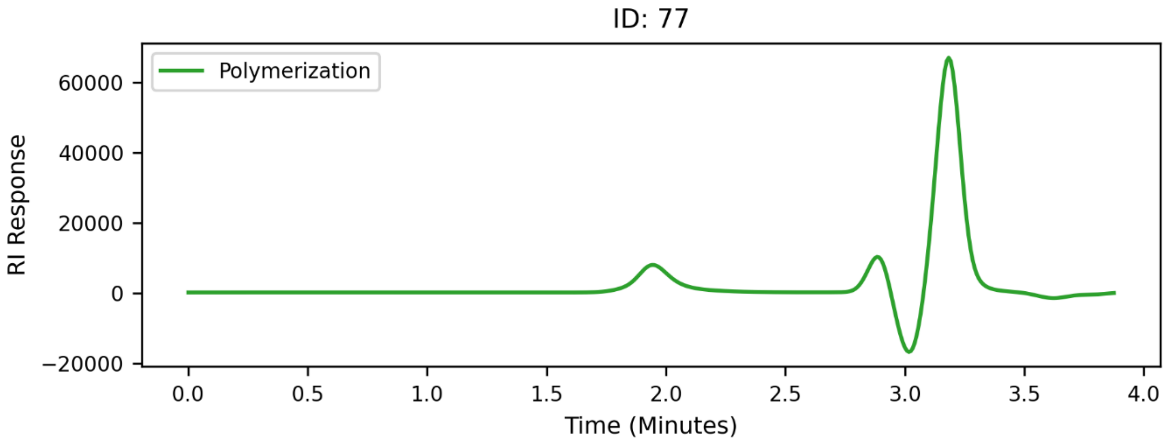
ID: 77
Polymerization
RI Response
Time (Minutes)
60000
40000
20000
0
−20000
0.0
0.5
1.0
1.5
2.0
2.5
3.0
3.5
4.0
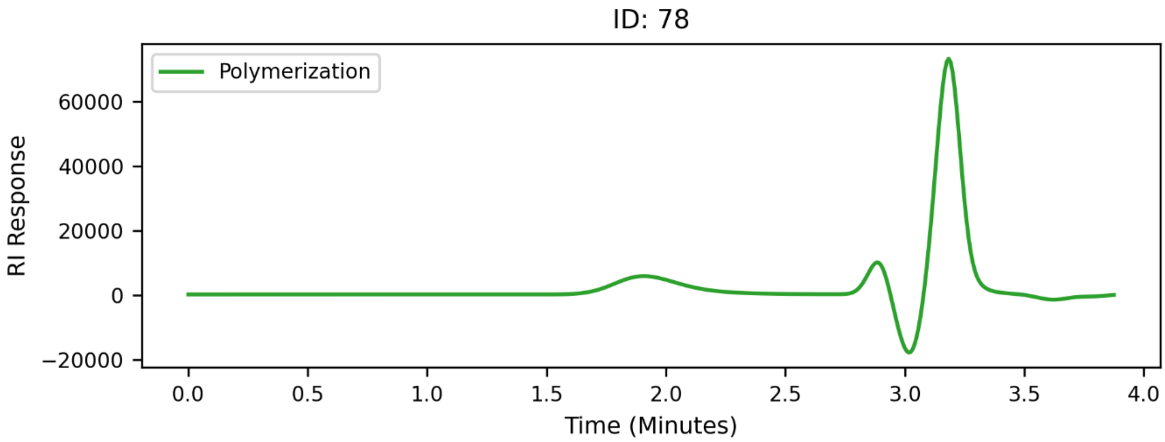
ID: 78
Polymerization
RI Response
Time (Minutes)
60000
40000
20000
0
−20000
0.0
0.5
1.0
1.5
2.0
2.5
3.0
3.5
4.0

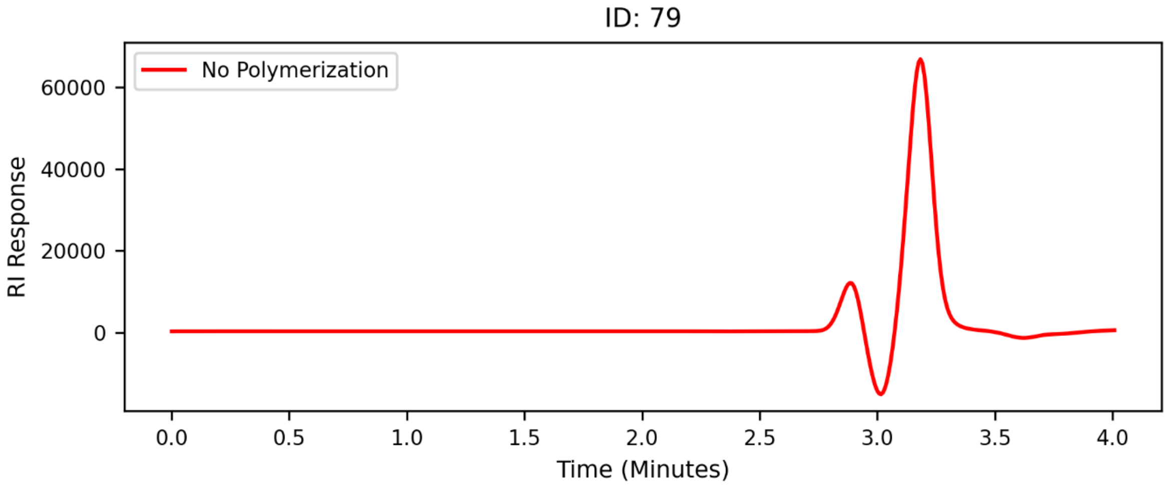
ID: 79
No Polymerization
RI Response
60000
40000
20000
0
0.0
0.5
1.0
1.5
2.0
2.5
3.0
3.5
4.0
Time (Minutes)

Appendix 1C: SEC Traces for Validation/ML Data (Batch 3)

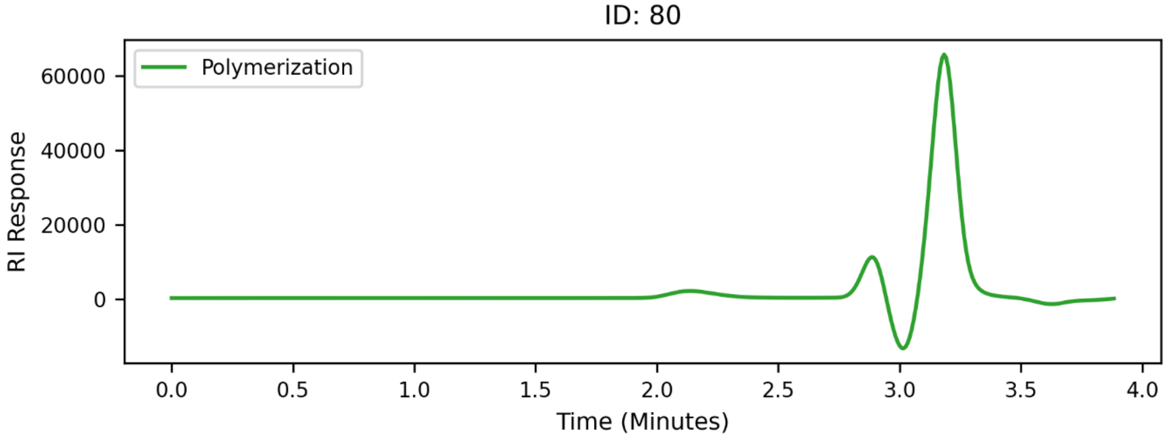

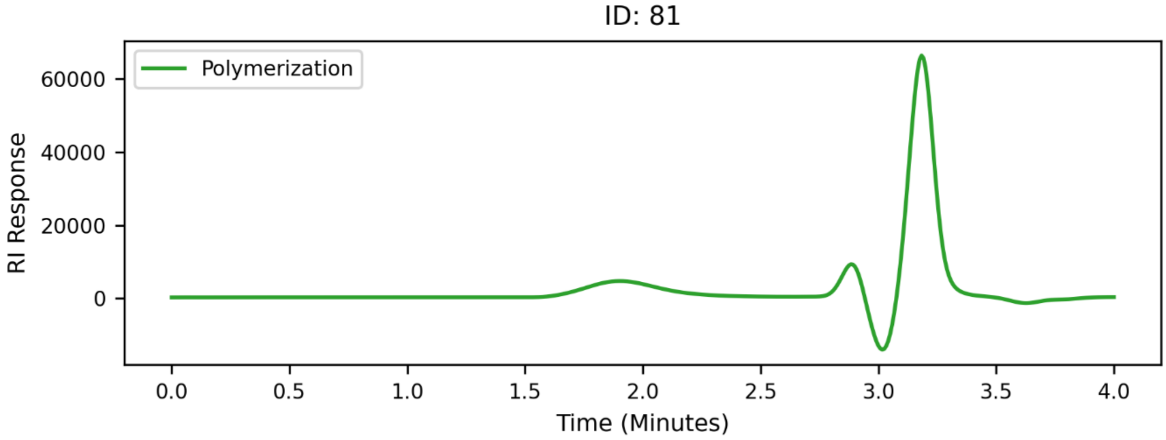

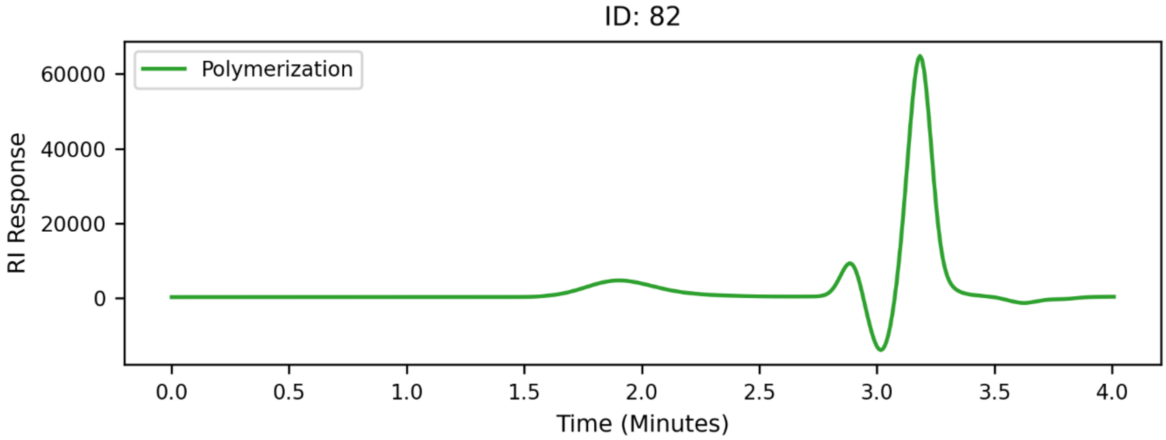

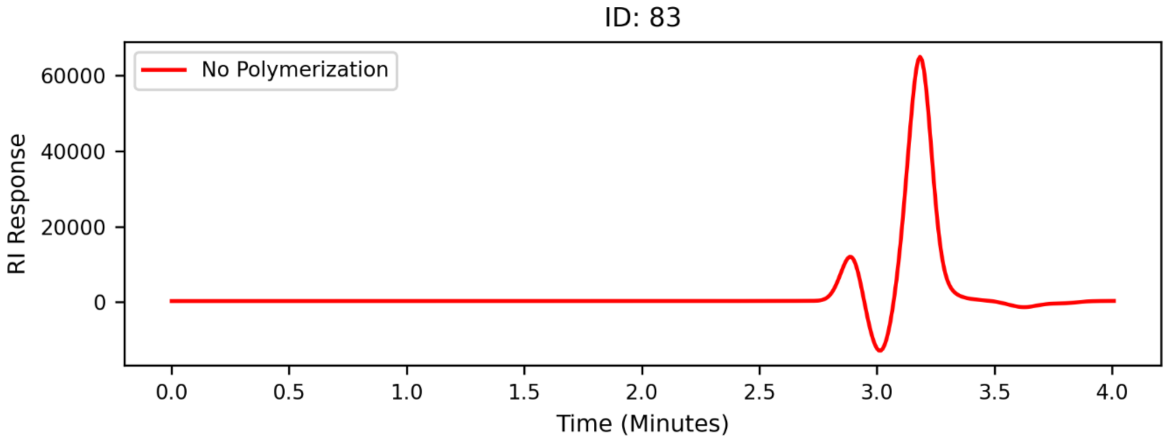

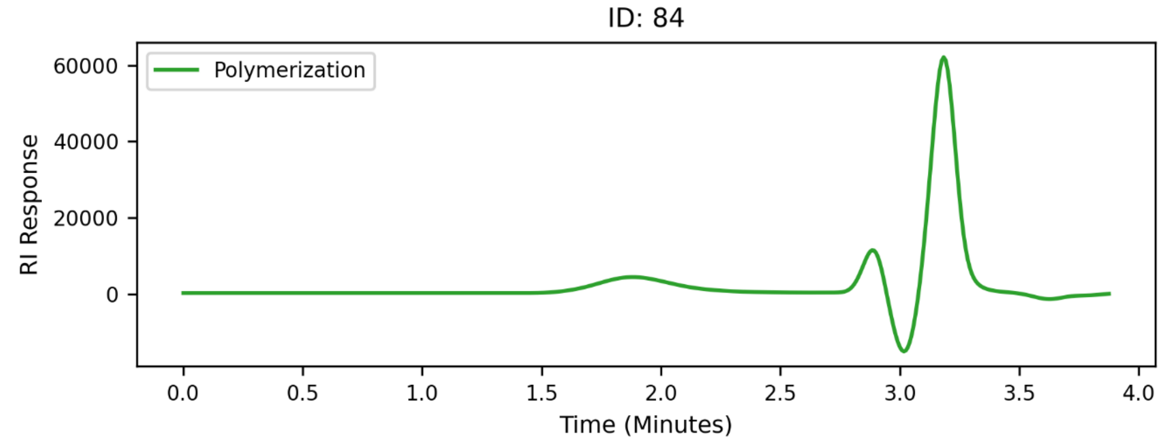
ID: 84
Polymerization
RI Response
Time (Minutes)
60000
40000
20000
0
0.0
0.5
1.0
1.5
2.0
2.5
3.0
3.5
4.0

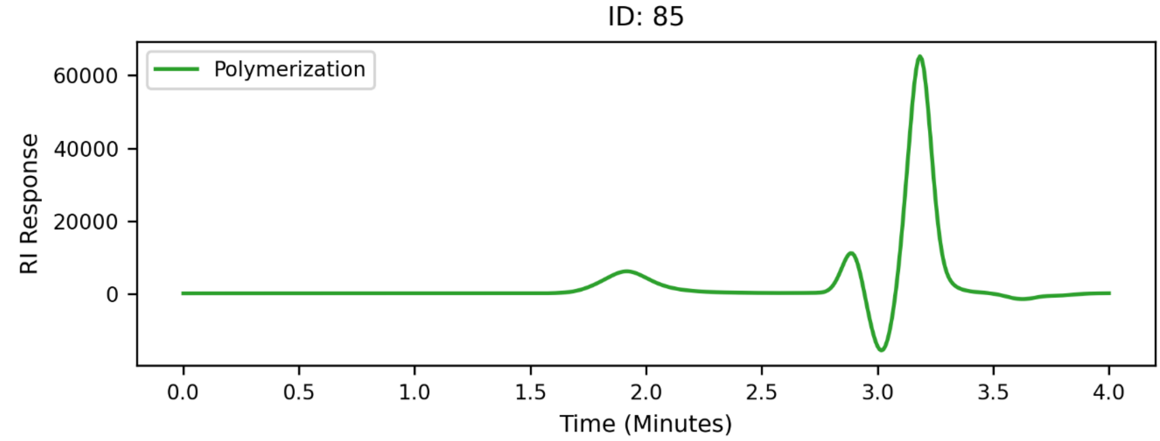
ID: 85
Polymerization
RI Response
Time (Minutes)
60000
40000
20000
0
0.0
0.5
1.0
1.5
2.0
2.5
3.0
3.5
4.0

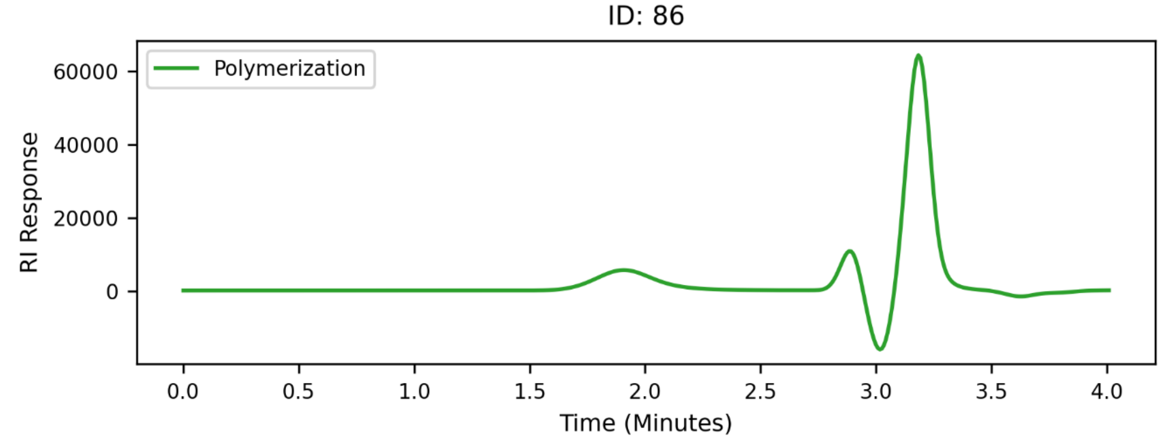
ID: 86
Polymerization
RI Response
Time (Minutes)
60000
40000
20000
0
0.0
0.5
1.0
1.5
2.0
2.5
3.0
3.5
4.0

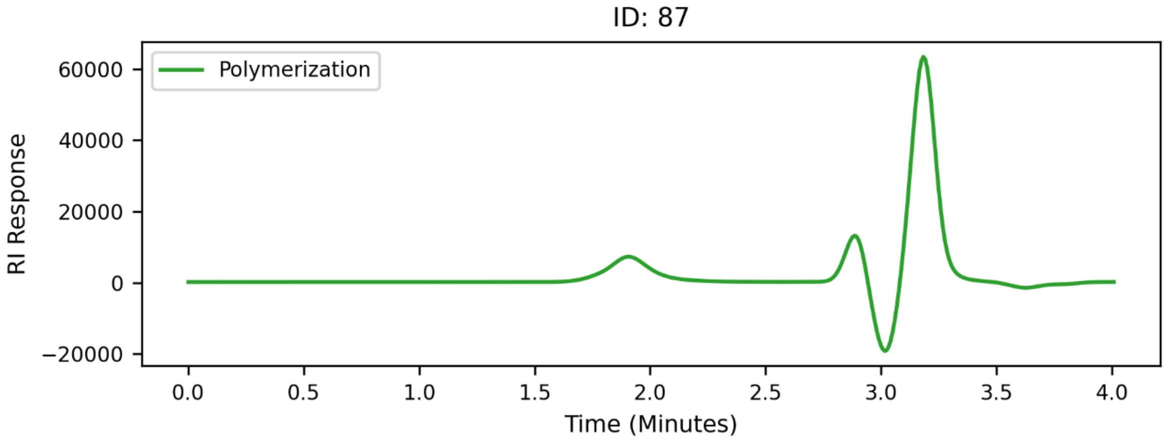
ID: 87
Polymerization
RI Response
Time (Minutes)
60000
40000
20000
0
−20000
0.0
0.5
1.0
1.5
2.0
2.5
3.0
3.5
4.0

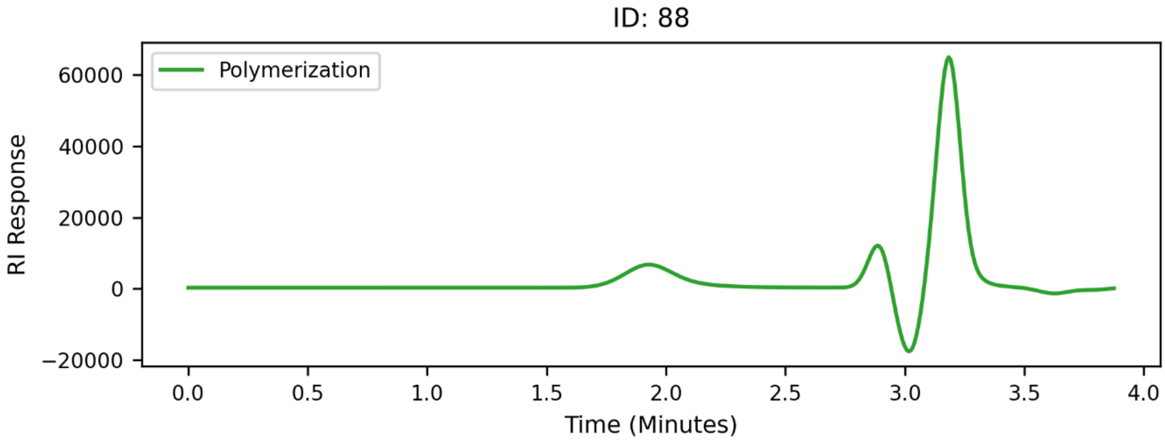
ID: 88
Polymerization
RI Response
60000
40000
20000
0
−20000
0.0
0.5
1.0
1.5
2.0
2.5
3.0
3.5
4.0
Time (Minutes)

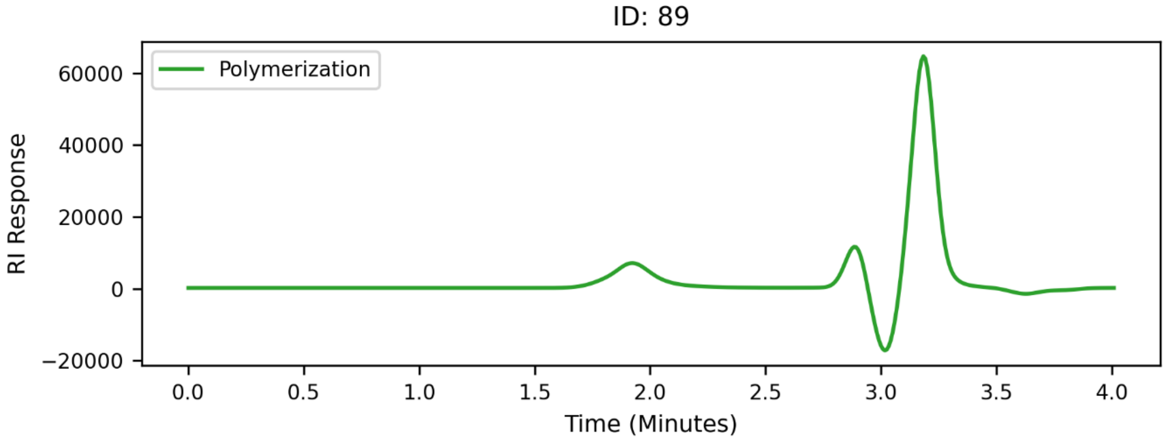
ID: 89
Polymerization
RI Response
60000
40000
20000
0
−20000
0.0
0.5
1.0
1.5
2.0
2.5
3.0
3.5
4.0
Time (Minutes)

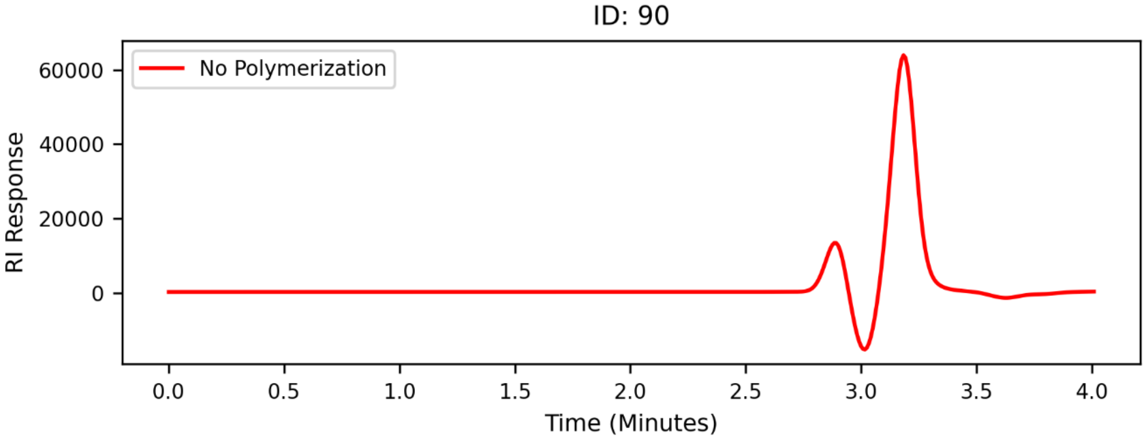
ID: 90
No Polymerization
RI Response
60000
40000
20000
0
0.0
0.5
1.0
1.5
2.0
2.5
3.0
3.5
4.0
Time (Minutes)

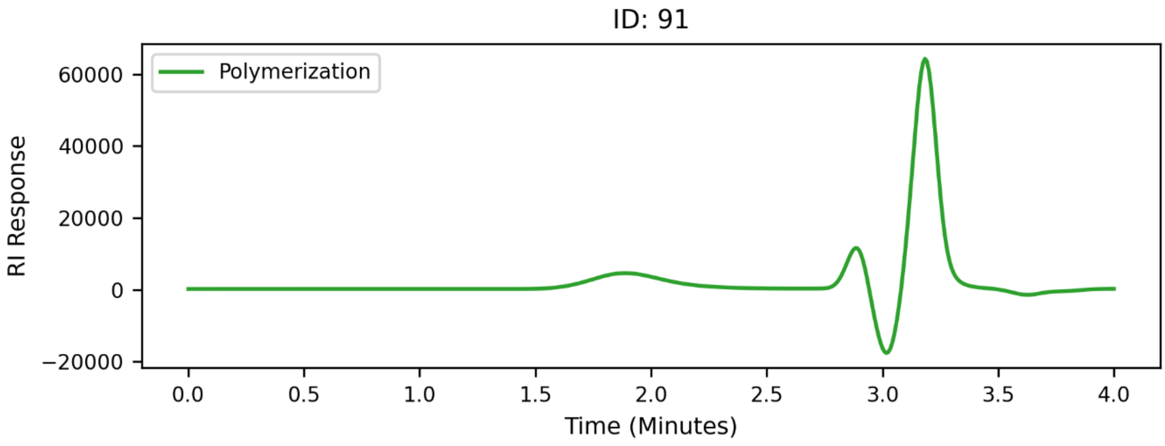
ID: 91
Polymerization
RI Response
60000
40000
20000
0
−20000
0.0
0.5
1.0
1.5
2.0
2.5
3.0
3.5
4.0
Time (Minutes)

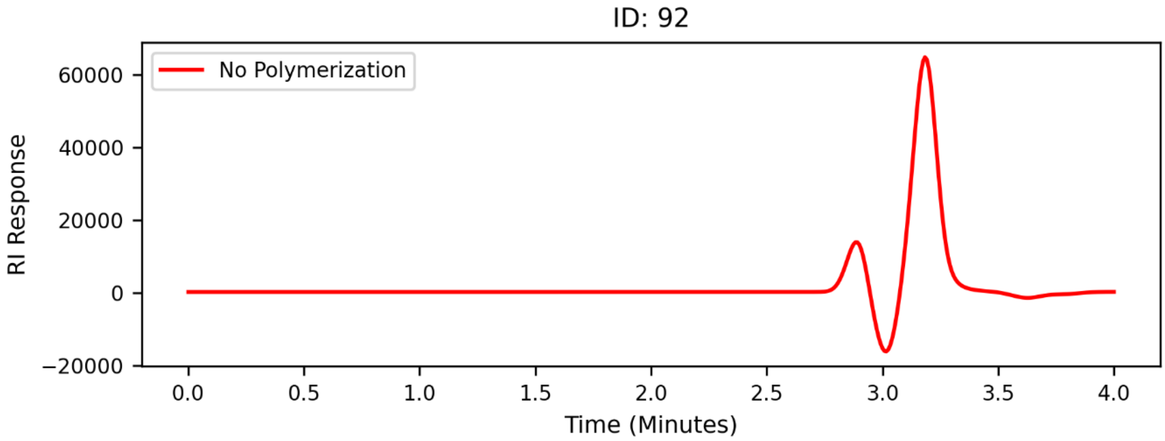
ID: 92
No Polymerization
RI Response
60000
40000
20000
0
−20000
0.0
0.5
1.0
1.5
2.0
2.5
3.0
3.5
4.0
Time (Minutes)

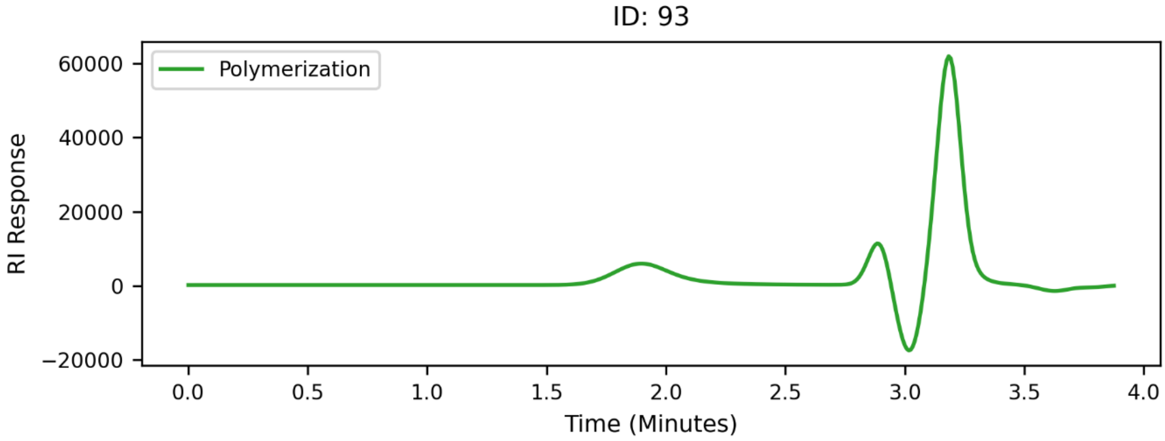
ID: 93
Polymerization
RI Response
60000
40000
20000
0
−20000
0.0
0.5
1.0
1.5
2.0
2.5
3.0
3.5
4.0
Time (Minutes)

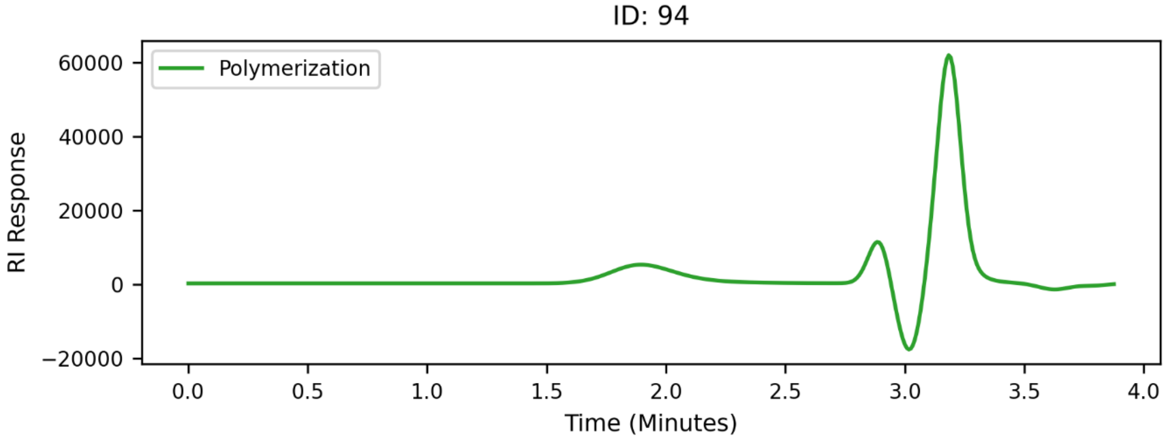
ID: 94
Polymerization
RI Response
60000
40000
20000
0
−20000
0.0
0.5
1.0
1.5
2.0
2.5
3.0
3.5
4.0
Time (Minutes)

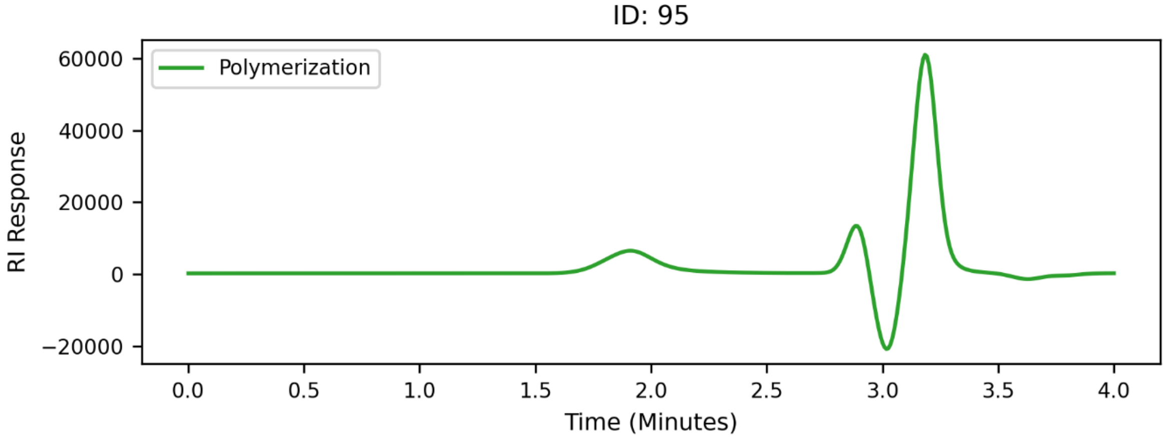
ID: 95
Polymerization
RI Response
60000
40000
20000
0
−20000
0.0
0.5
1.0
1.5
2.0
2.5
3.0
3.5
4.0
Time (Minutes)

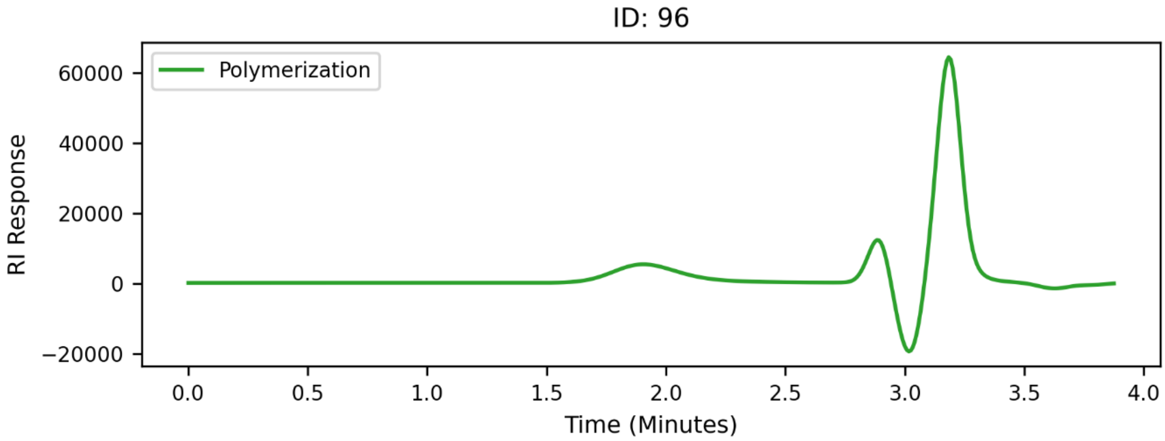
ID: 96
Polymerization
RI Response
Time (Minutes)

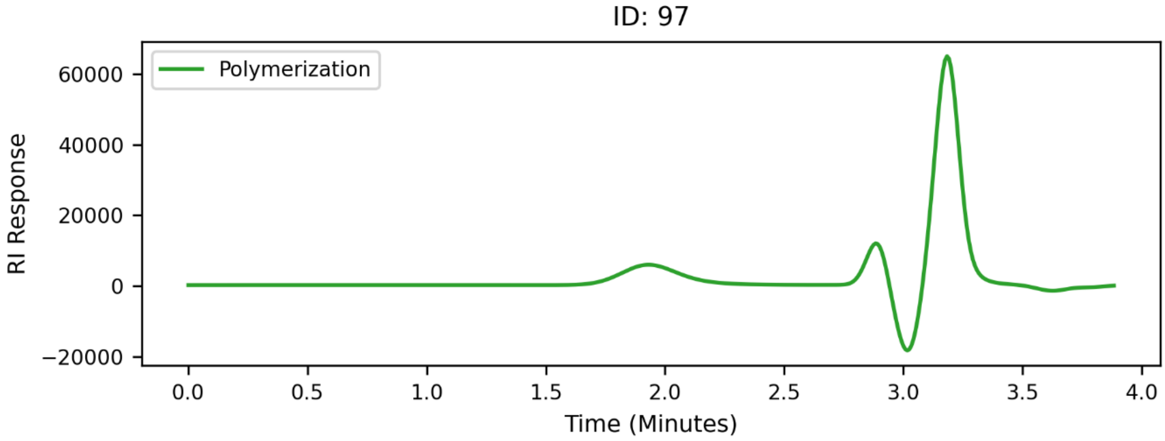
ID: 97
Polymerization
RI Response
Time (Minutes)

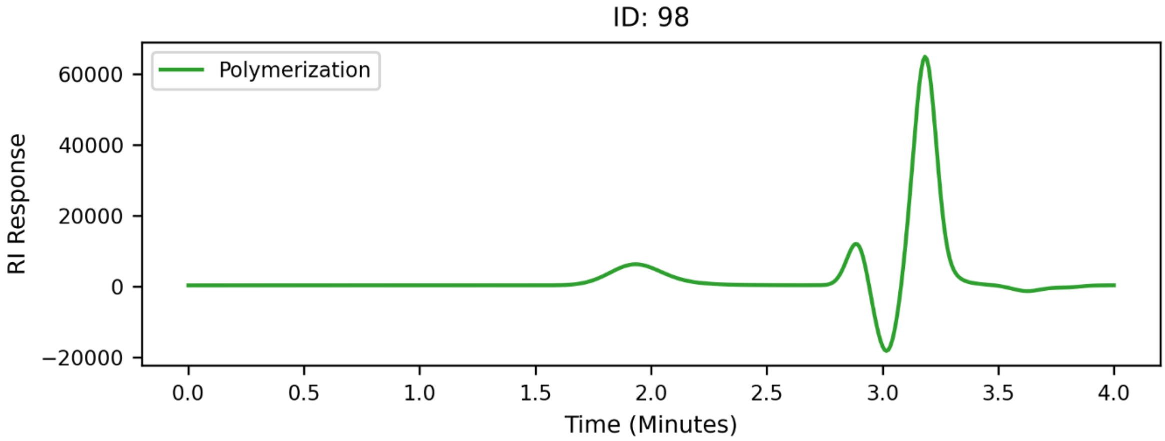
ID: 98
Polymerization
RI Response
Time (Minutes)

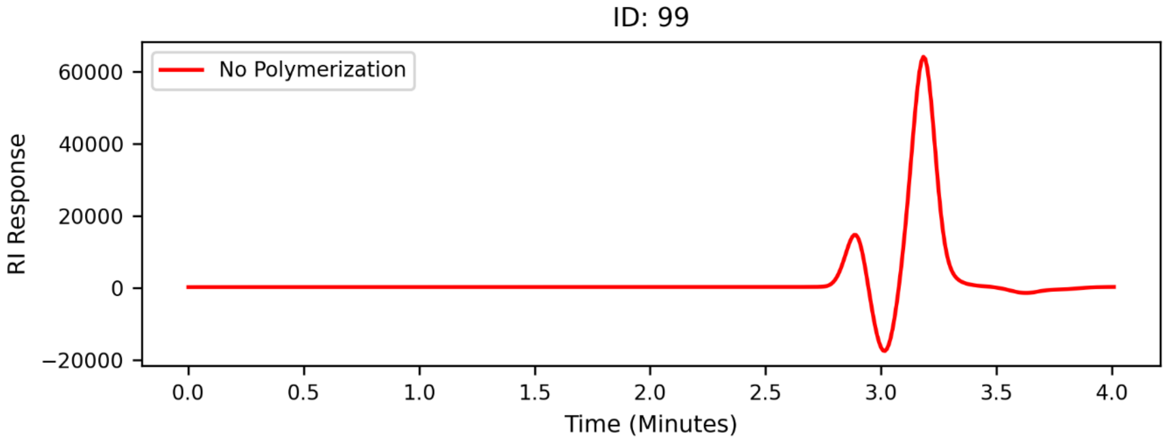
ID: 99
No Polymerization
RI Response
Time (Minutes)

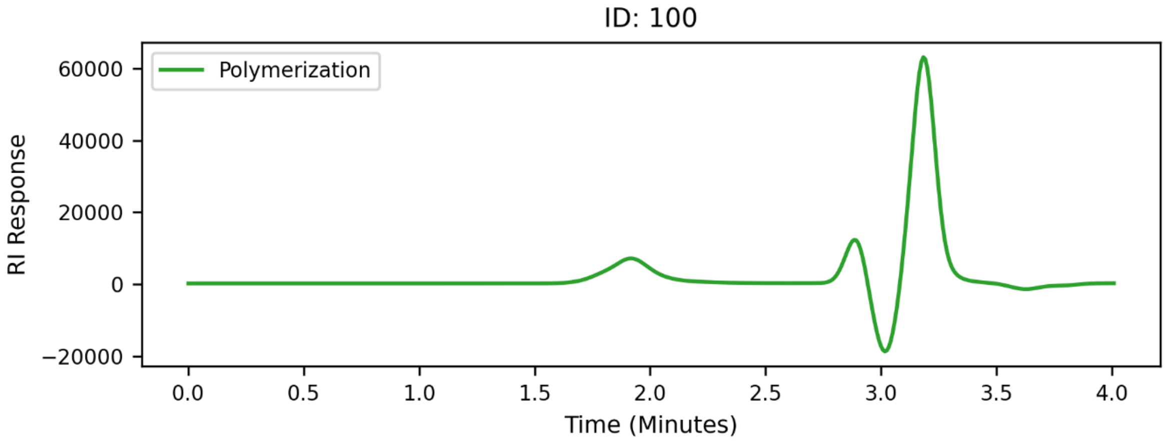
ID: 100
Polymerization
RI Response
60000
40000
20000
0
−20000
0.0
0.5
1.0
1.5
2.0
2.5
3.0
3.5
4.0
Time (Minutes)

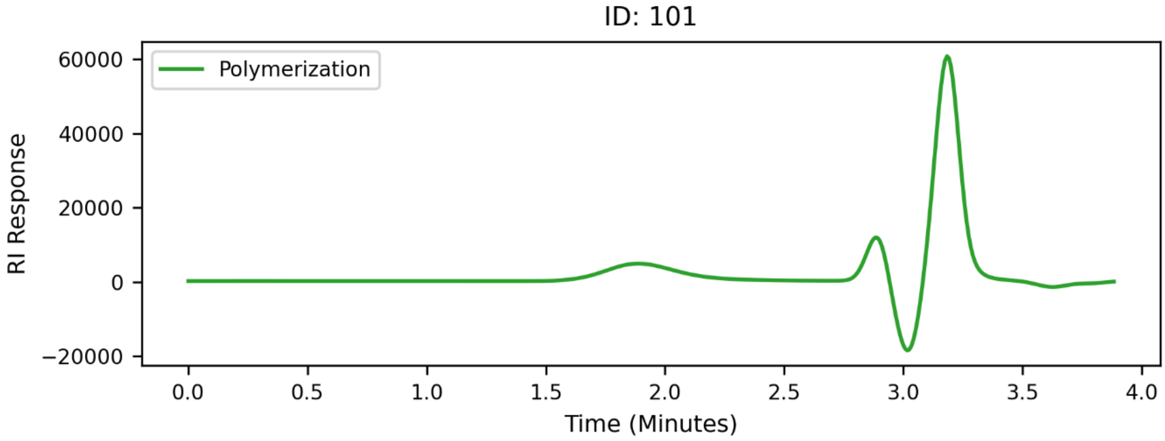
ID: 101
Polymerization
RI Response
60000
40000
20000
0
−20000
0.0
0.5
1.0
1.5
2.0
2.5
3.0
3.5
4.0
Time (Minutes)

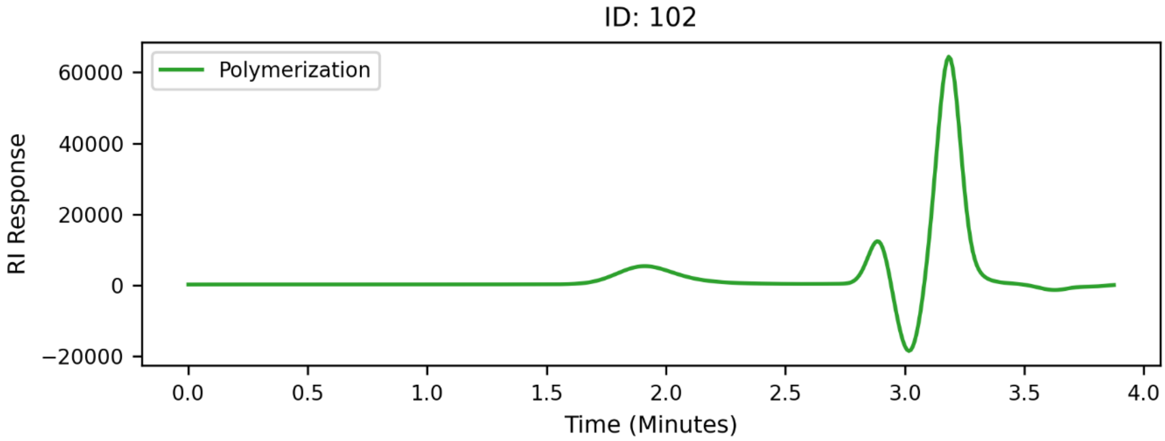
ID: 102
Polymerization
RI Response
60000
40000
20000
0
−20000
0.0
0.5
1.0
1.5
2.0
2.5
3.0
3.5
4.0
Time (Minutes)

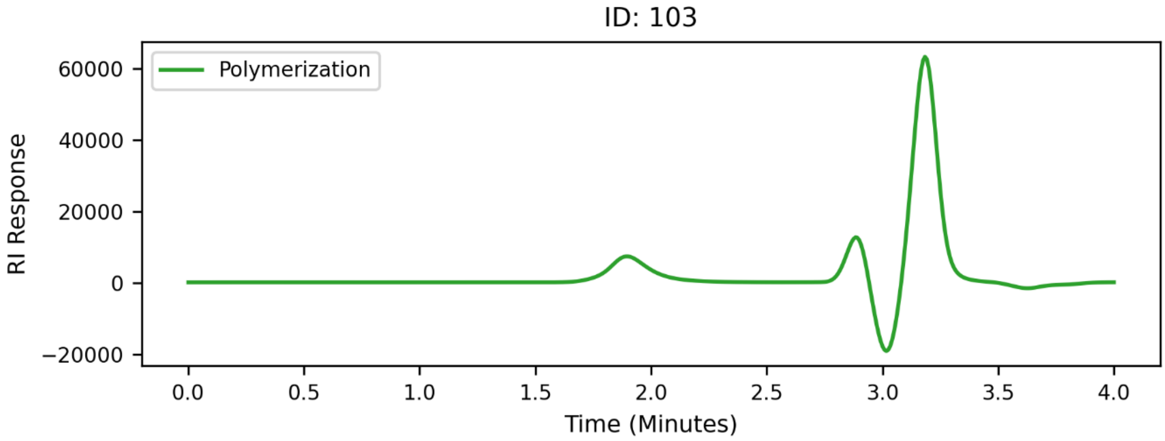
ID: 103
Polymerization
RI Response
60000
40000
20000
0
−20000
0.0
0.5
1.0
1.5
2.0
2.5
3.0
3.5
4.0
Time (Minutes)

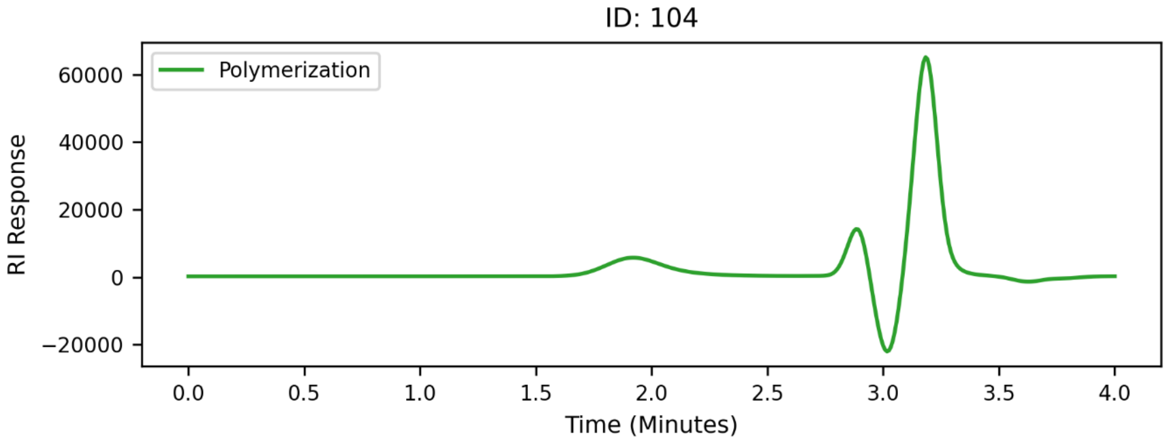
ID: 104
Polymerization
RI Response
Time (Minutes)

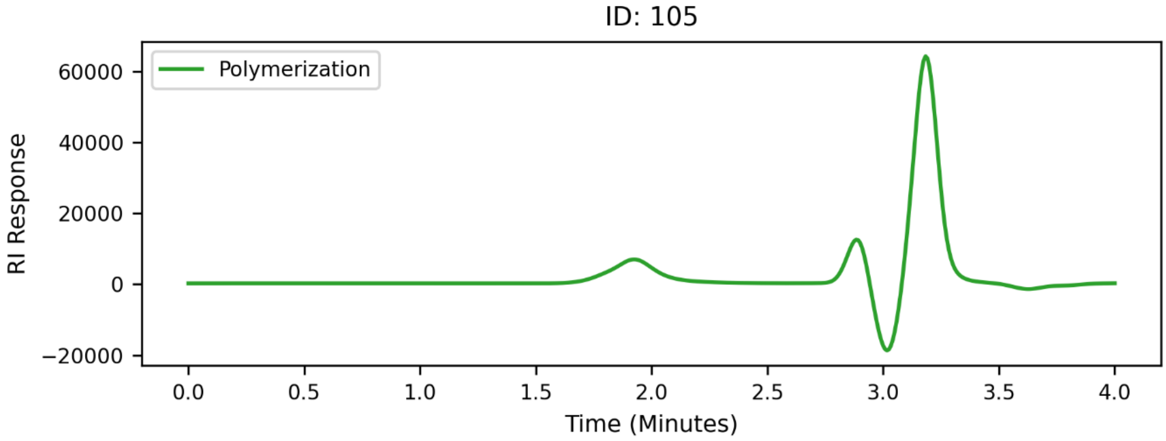
ID: 105
Polymerization
RI Response
Time (Minutes)

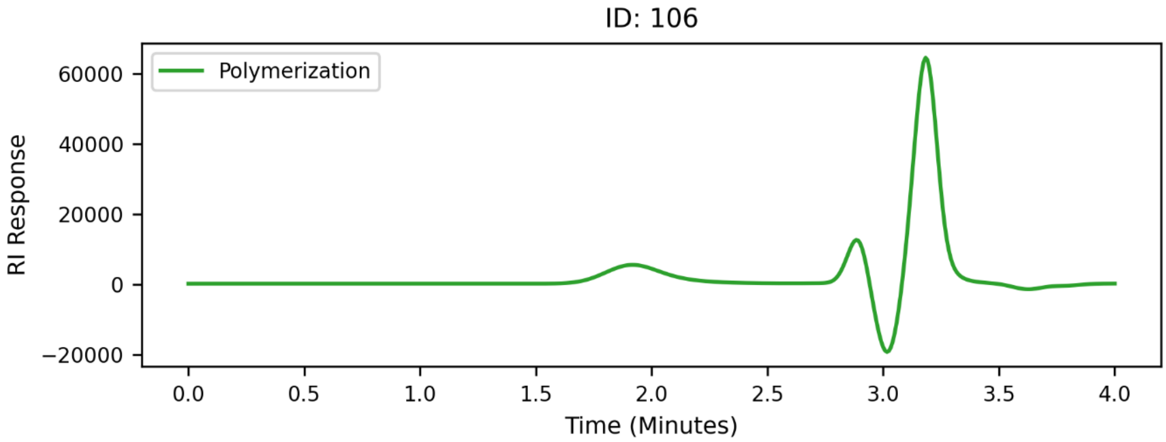
ID: 106
Polymerization
RI Response
Time (Minutes)

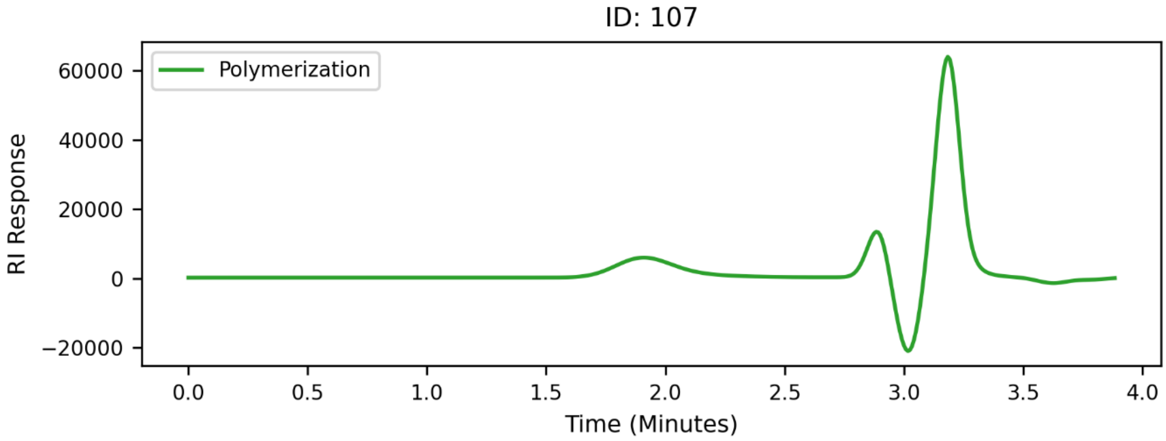
ID: 107
Polymerization
RI Response
Time (Minutes)

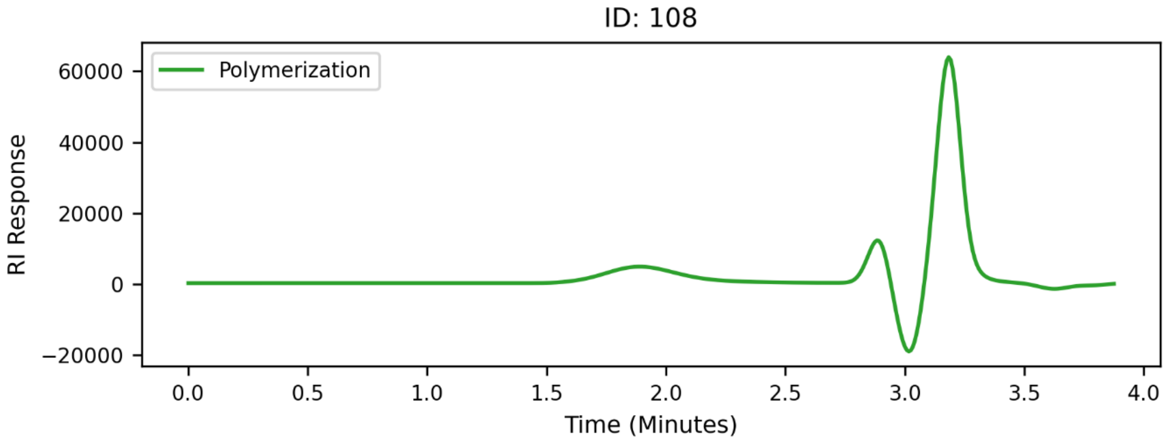
ID: 108
Polymerization
RI Response
60000
40000
20000
0
−20000
0.0
0.5
1.0
1.5
2.0
2.5
3.0
3.5
4.0
Time (Minutes)

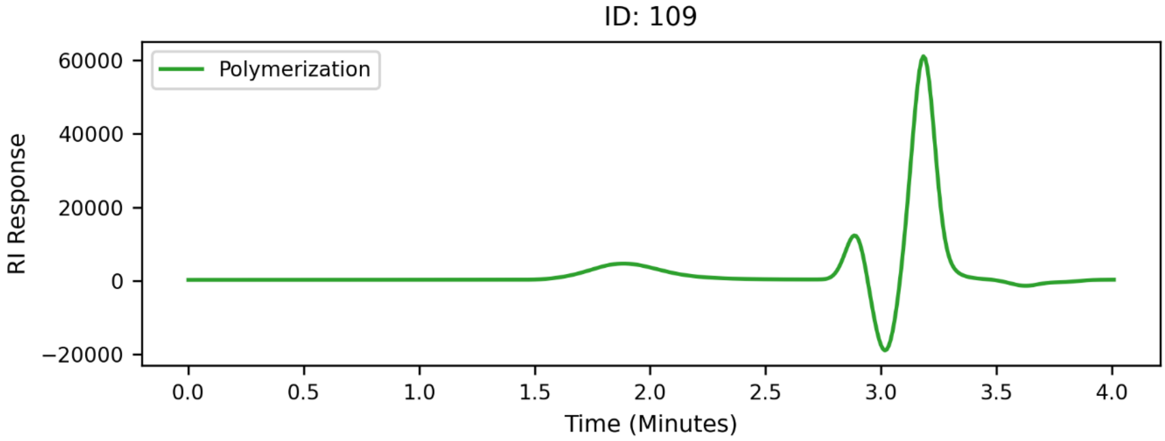
ID: 109
Polymerization
RI Response
60000
40000
20000
0
−20000
0.0
0.5
1.0
1.5
2.0
2.5
3.0
3.5
4.0
Time (Minutes)

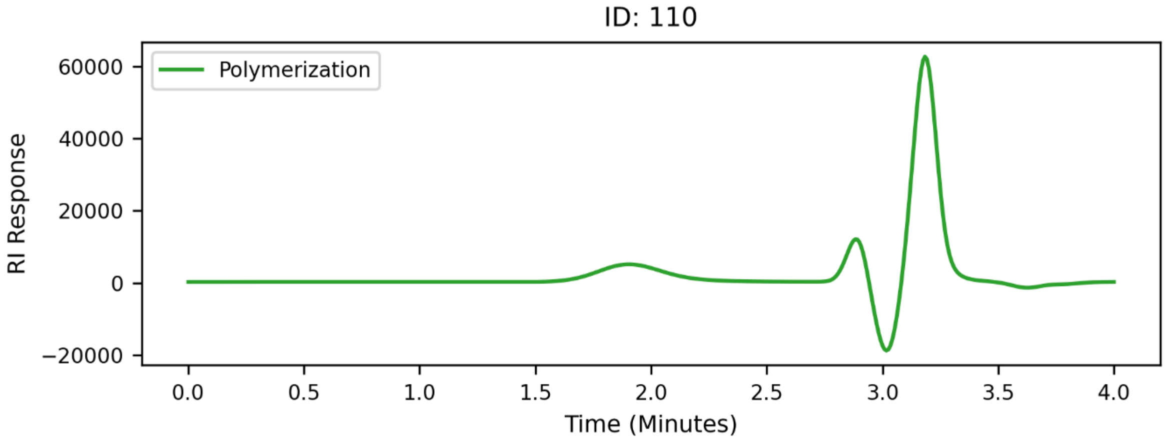
ID: 110
Polymerization
RI Response
60000
40000
20000
0
−20000
0.0
0.5
1.0
1.5
2.0
2.5
3.0
3.5
4.0
Time (Minutes)

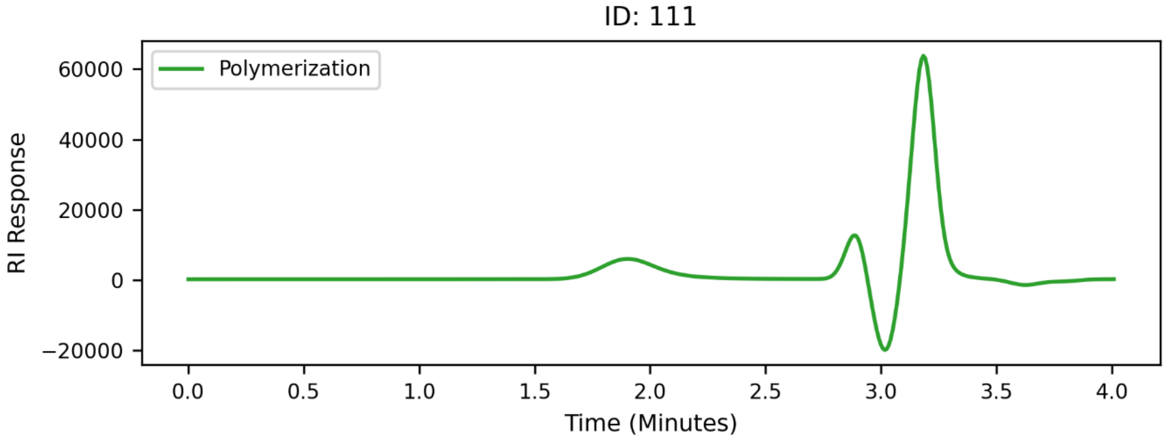
ID: 111
Polymerization
RI Response
60000
40000
20000
0
−20000
0.0
0.5
1.0
1.5
2.0
2.5
3.0
3.5
4.0
Time (Minutes)

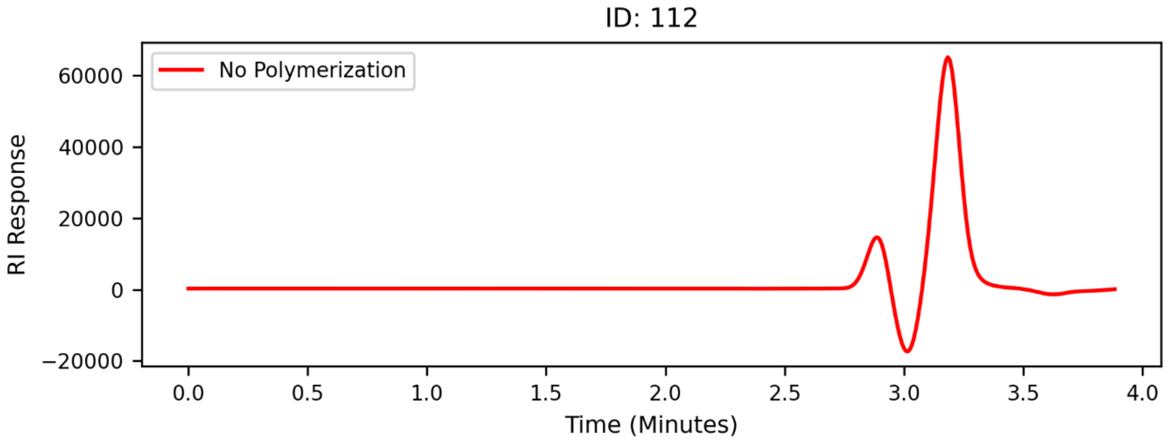
ID: 112
No Polymerization
RI Response
Time (Minutes)

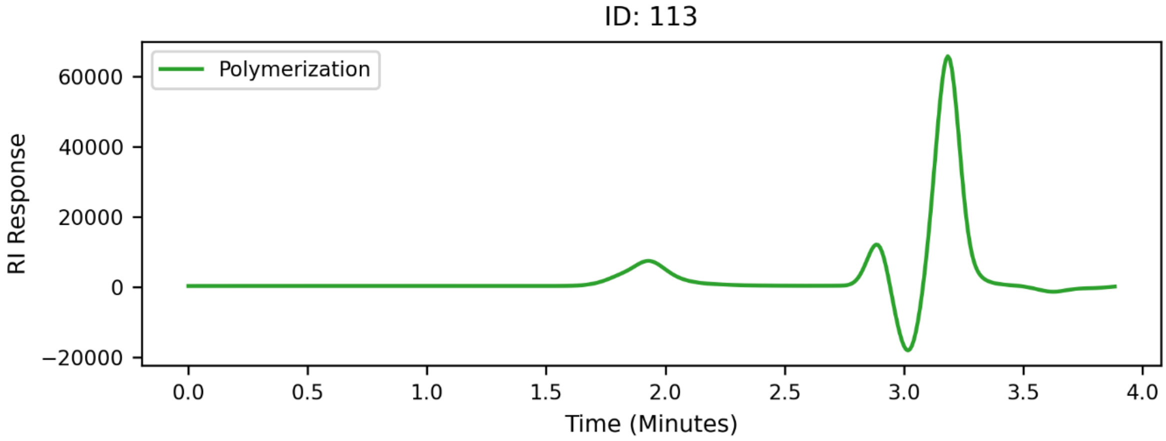
ID: 113
Polymerization
RI Response
Time (Minutes)

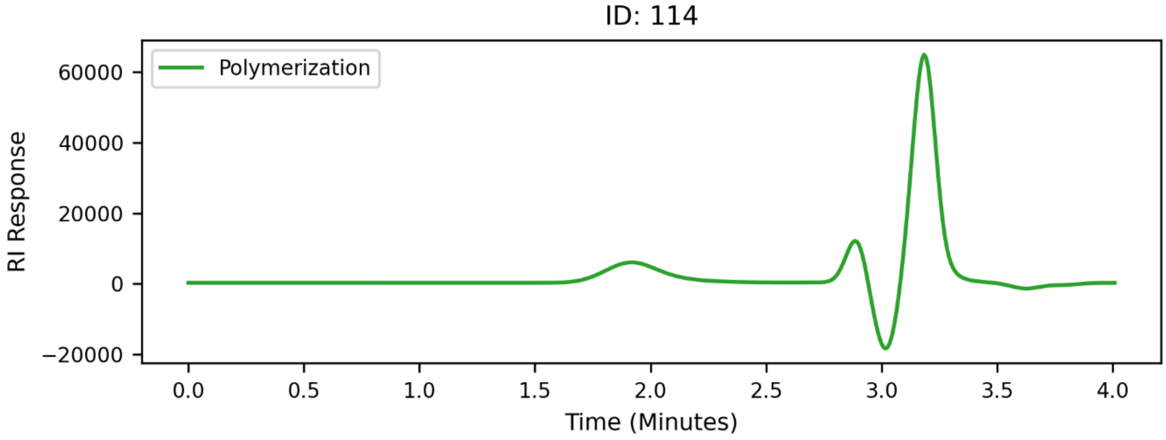
ID: 114
Polymerization
RI Response
Time (Minutes)

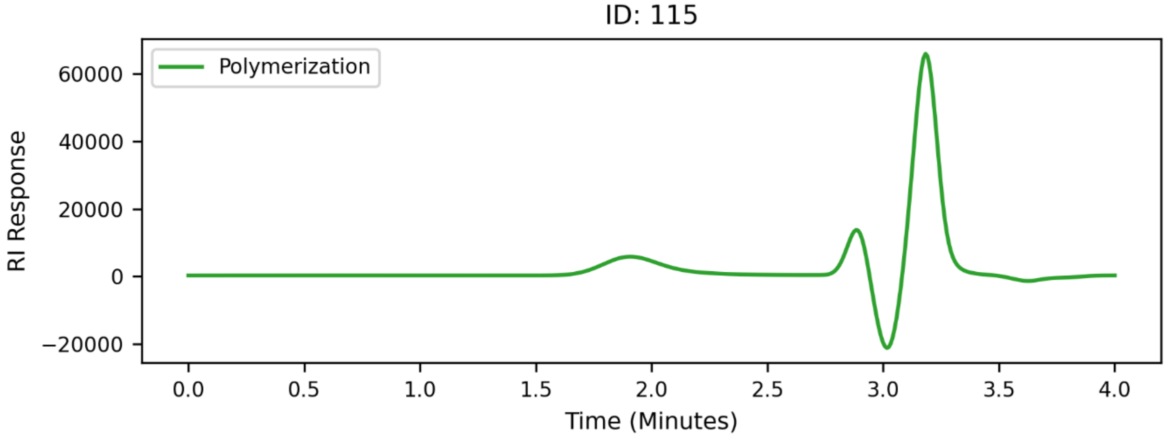
ID: 115
Polymerization
RI Response
Time (Minutes)

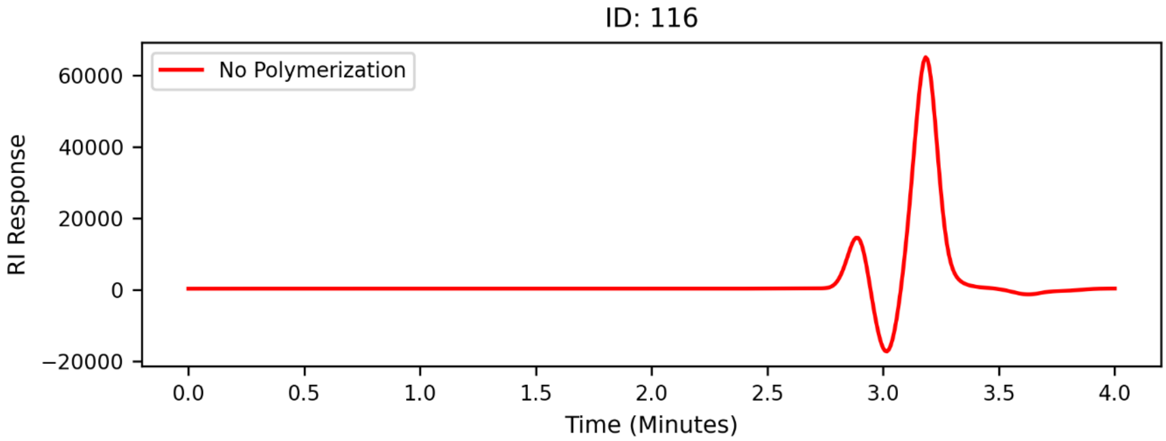
ID: 116
No Polymerization
RI Response
60000
40000
20000
0
−20000
0.0
0.5
1.0
1.5
2.0
2.5
3.0
3.5
4.0
Time (Minutes)

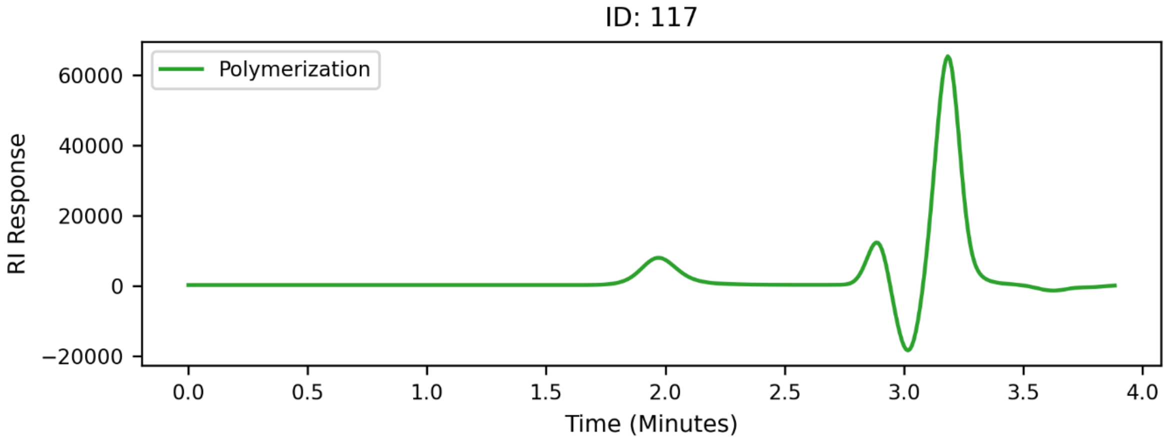
ID: 117
Polymerization
RI Response
60000
40000
20000
0
−20000
0.0
0.5
1.0
1.5
2.0
2.5
3.0
3.5
4.0
Time (Minutes)

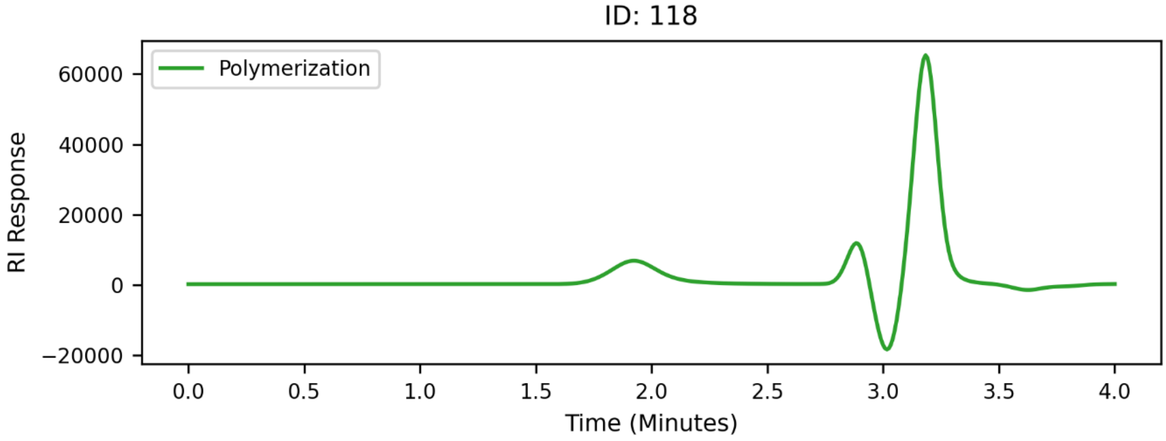
ID: 118
Polymerization
RI Response
60000
40000
20000
0
−20000
0.0
0.5
1.0
1.5
2.0
2.5
3.0
3.5
4.0
Time (Minutes)

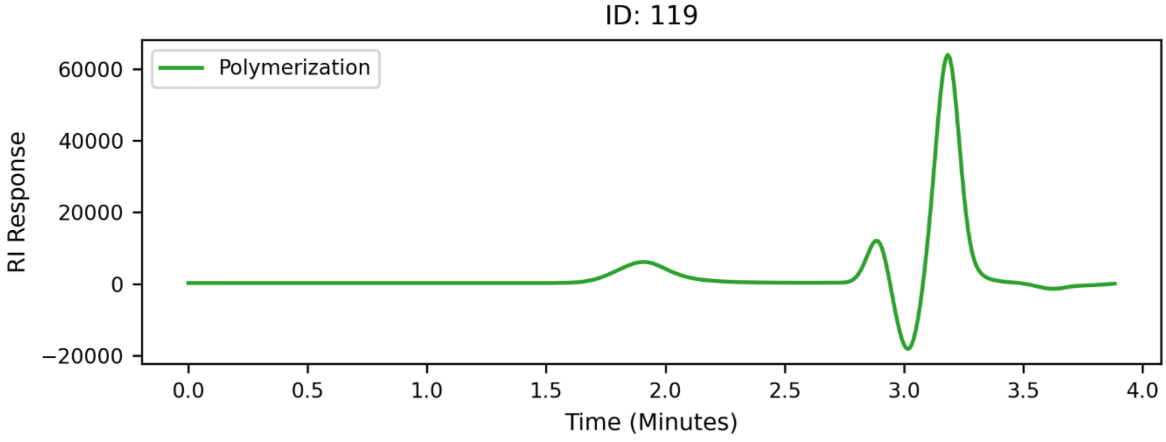
ID: 119
Polymerization
RI Response
60000
40000
20000
0
−20000
0.0
0.5
1.0
1.5
2.0
2.5
3.0
3.5
4.0
Time (Minutes)

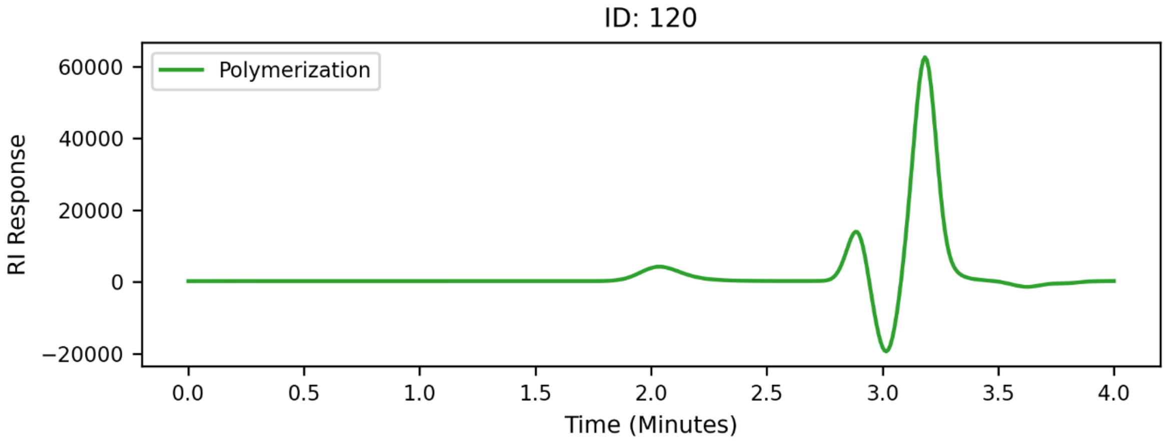
ID: 120
Polymerization
RI Response
Time (Minutes)
60000
40000
20000
0
−20000
0.0
0.5
1.0
1.5
2.0
2.5
3.0
3.5
4.0

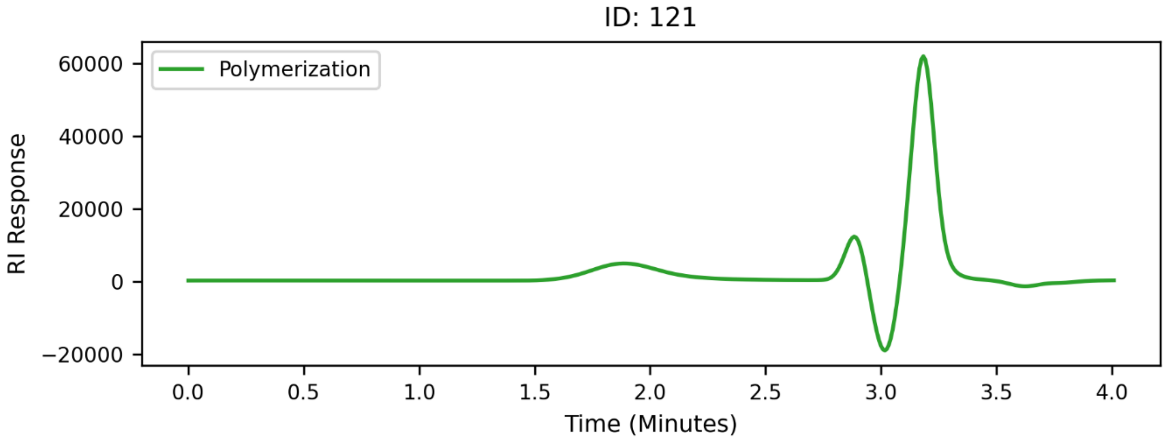
ID: 121
Polymerization
RI Response
Time (Minutes)
60000
40000
20000
0
−20000
0.0
0.5
1.0
1.5
2.0
2.5
3.0
3.5
4.0

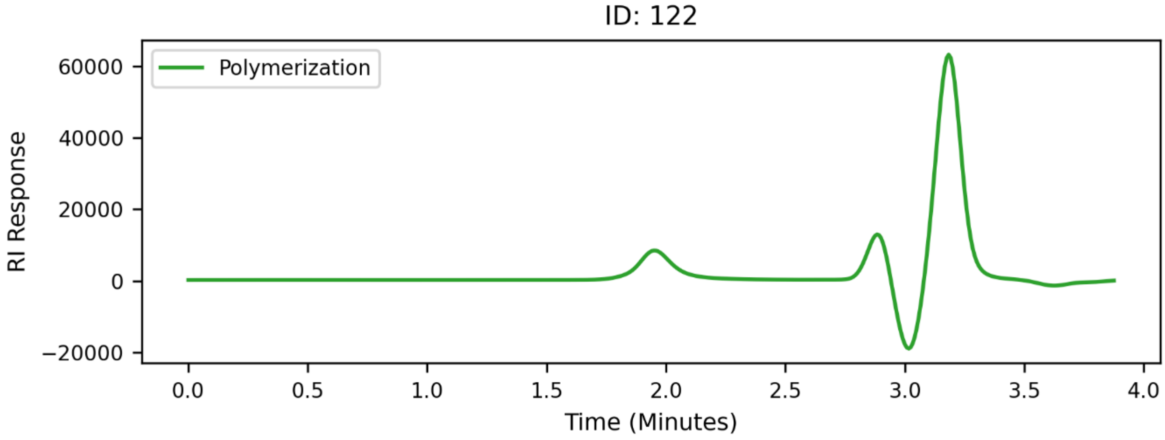
ID: 122
Polymerization
RI Response
Time (Minutes)
60000
40000
20000
0
−20000
0.0
0.5
1.0
1.5
2.0
2.5
3.0
3.5
4.0

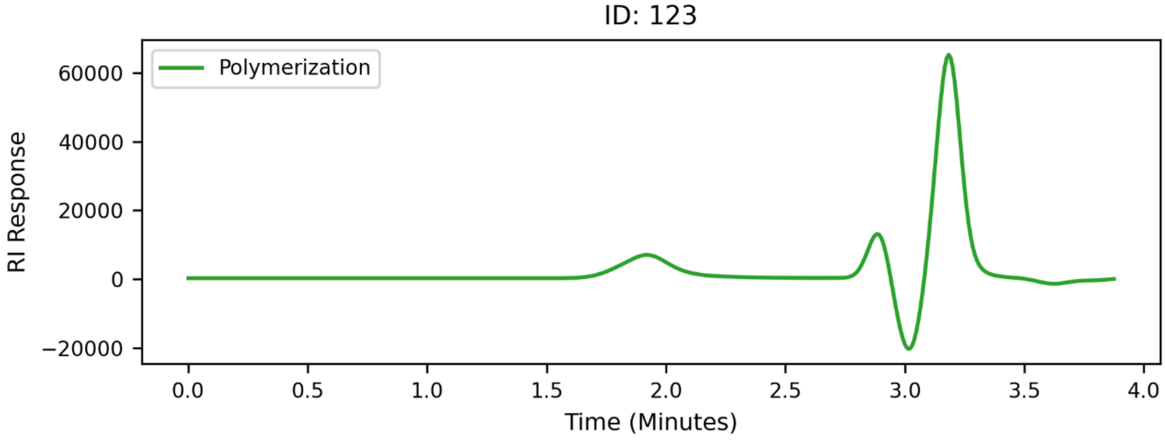
ID: 123
Polymerization
RI Response
Time (Minutes)
60000
40000
20000
0
−20000
0.0
0.5
1.0
1.5
2.0
2.5
3.0
3.5
4.0

ID: 124

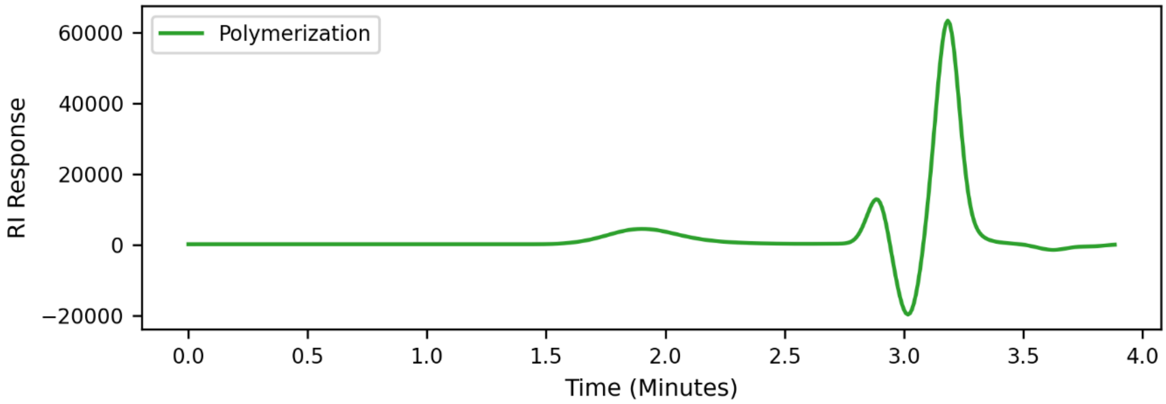

ID: 125

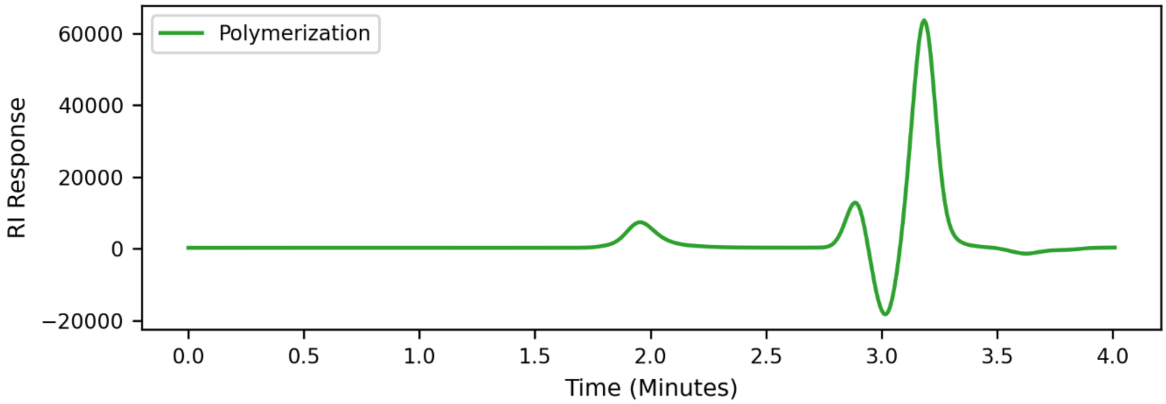

ID: 126

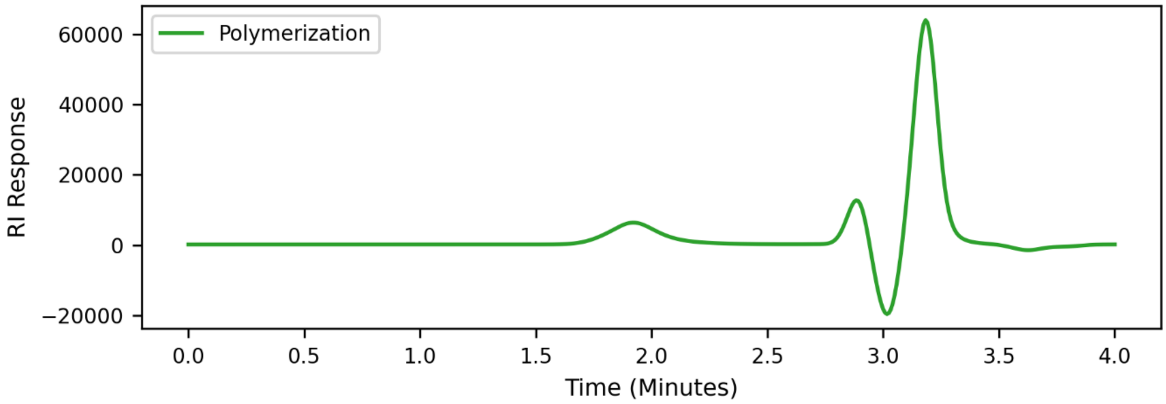

ID: 127

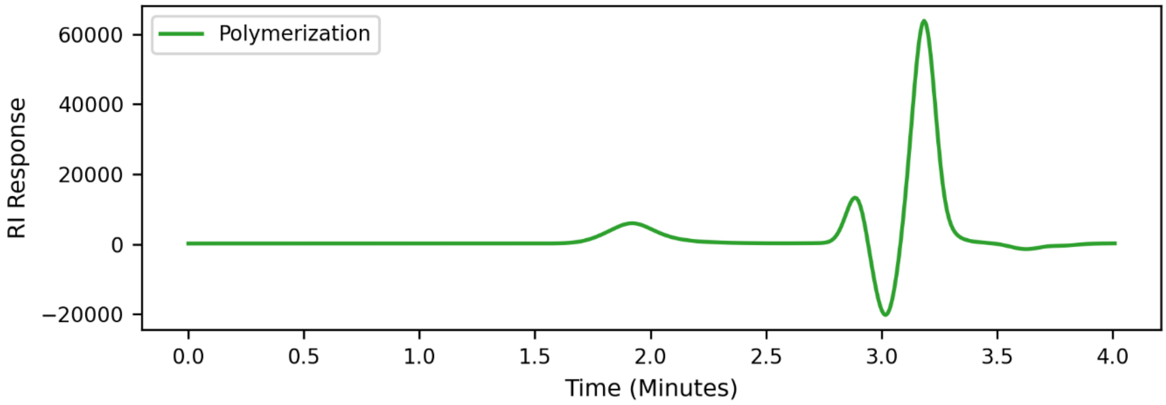

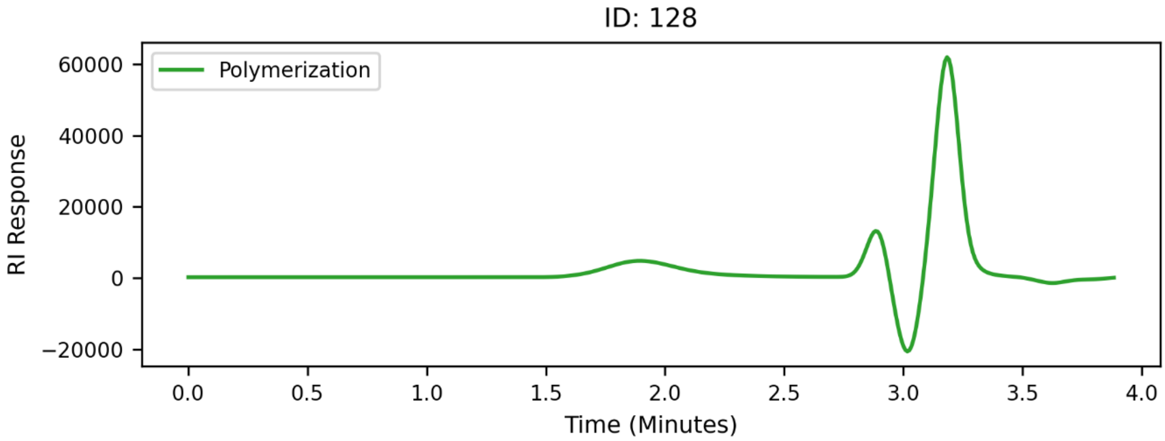
ID: 128
Polymerization
RI Response
Time (Minutes)
60000
40000
20000
0
−20000
0.0
0.5
1.0
1.5
2.0
2.5
3.0
3.5
4.0

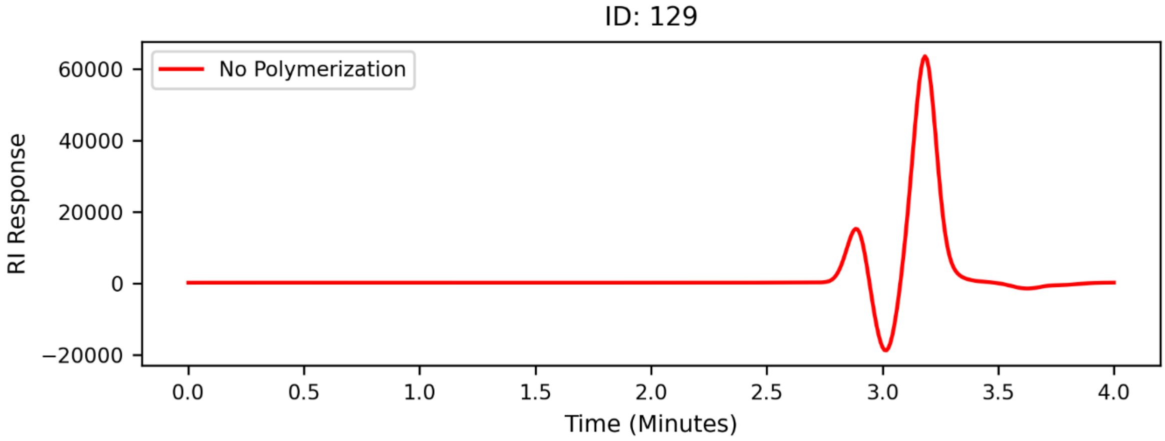
ID: 129
No Polymerization
RI Response
Time (Minutes)
60000
40000
20000
0
−20000
0.0
0.5
1.0
1.5
2.0
2.5
3.0
3.5
4.0

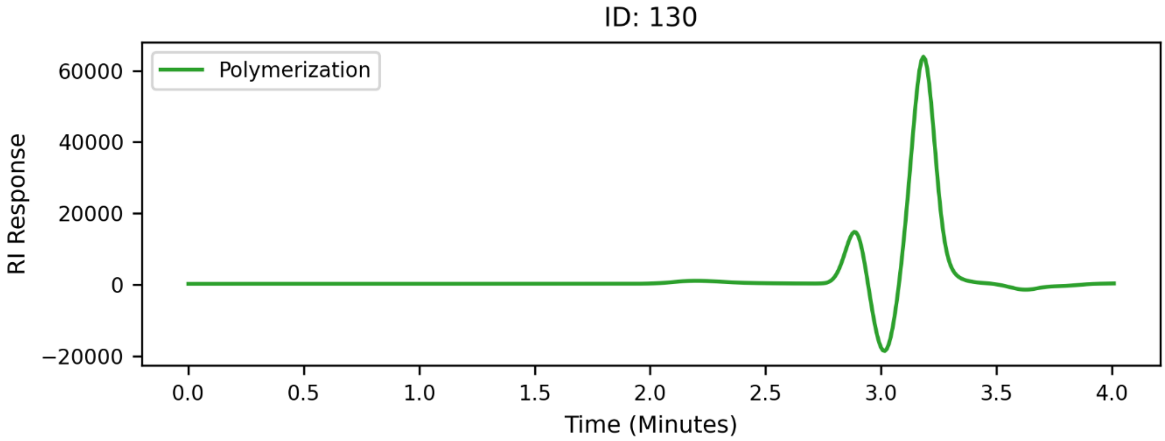
ID: 130
Polymerization
RI Response
Time (Minutes)
60000
40000
20000
0
−20000
0.0
0.5
1.0
1.5
2.0
2.5
3.0
3.5
4.0

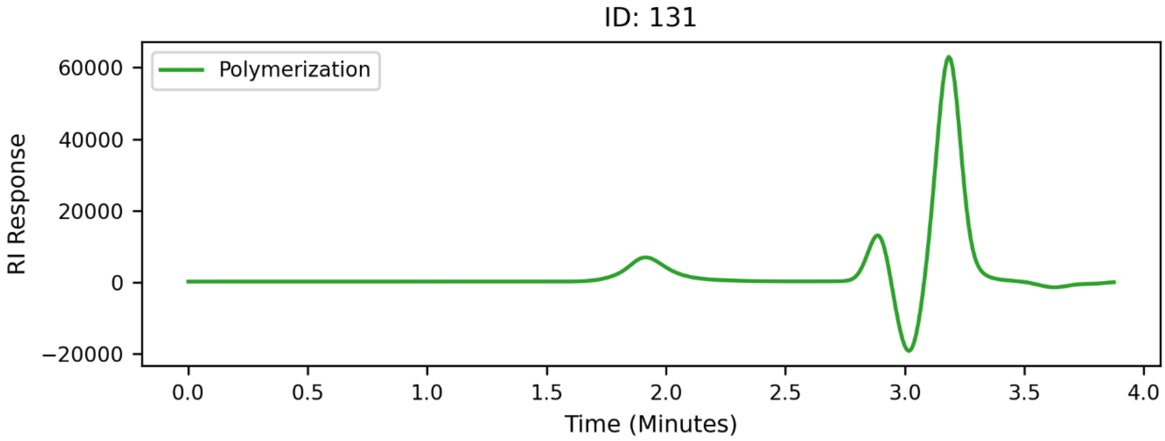
ID: 131
Polymerization
RI Response
Time (Minutes)
60000
40000
20000
0
−20000
0.0
0.5
1.0
1.5
2.0
2.5
3.0
3.5
4.0

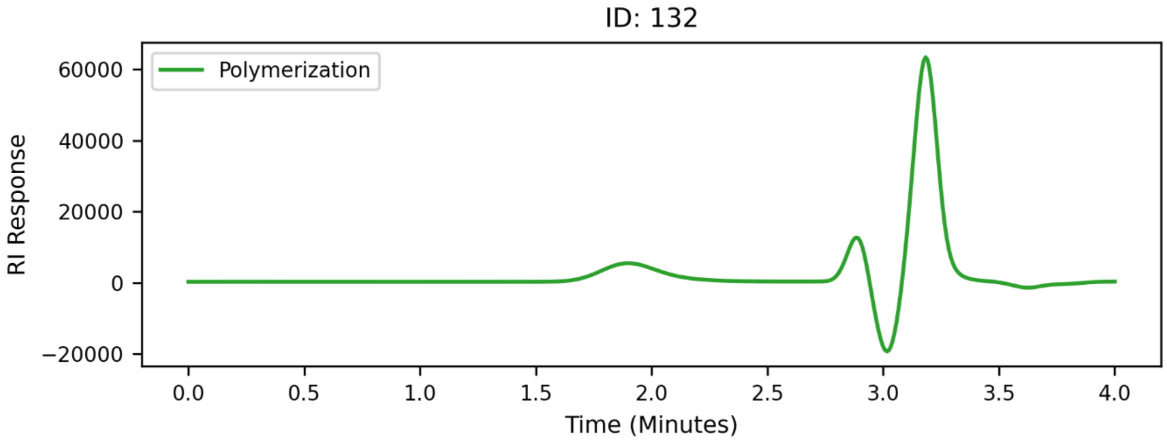
ID: 132
Polymerization
RI Response
60000
40000
20000
0
−20000
0.0
0.5
1.0
1.5
2.0
2.5
3.0
3.5
4.0
Time (Minutes)

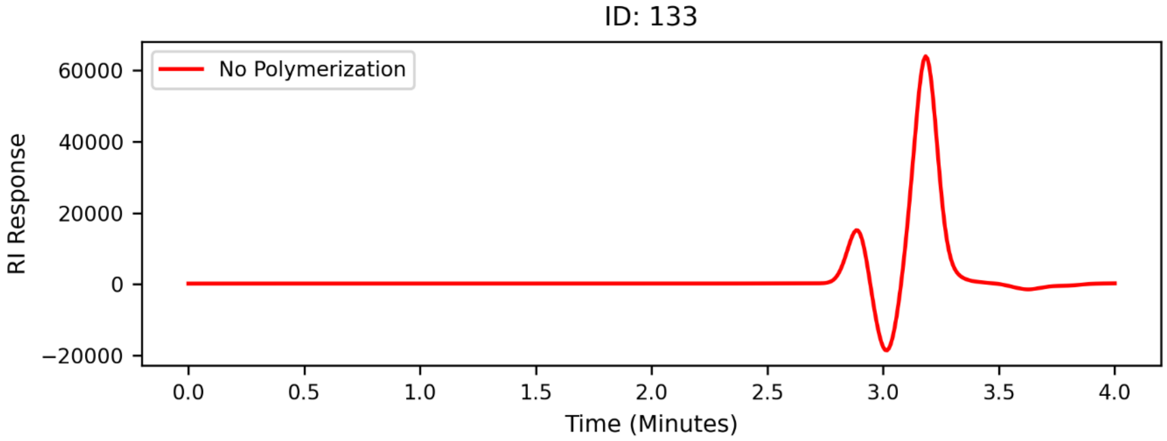
ID: 133
No Polymerization
RI Response
60000
40000
20000
0
−20000
0.0
0.5
1.0
1.5
2.0
2.5
3.0
3.5
4.0
Time (Minutes)

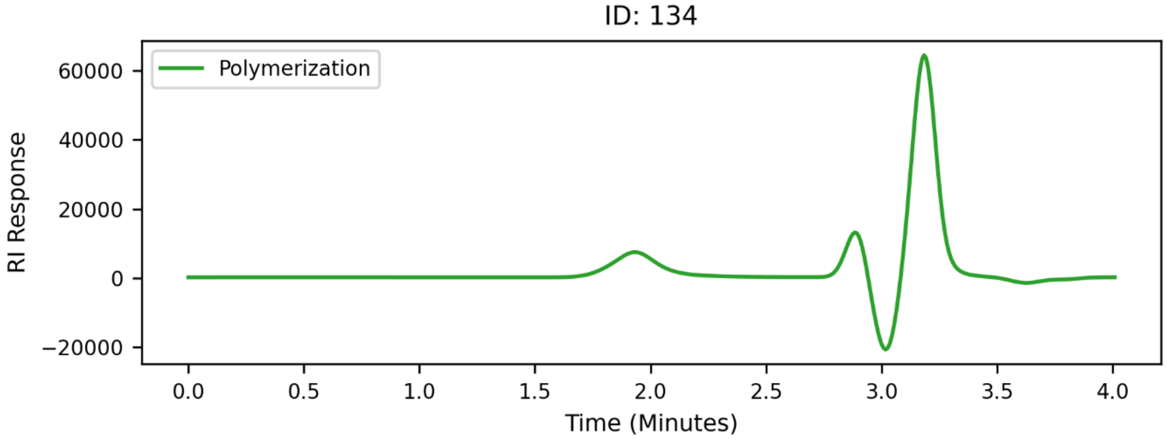
ID: 134
Polymerization
RI Response
60000
40000
20000
0
−20000
0.0
0.5
1.0
1.5
2.0
2.5
3.0
3.5
4.0
Time (Minutes)

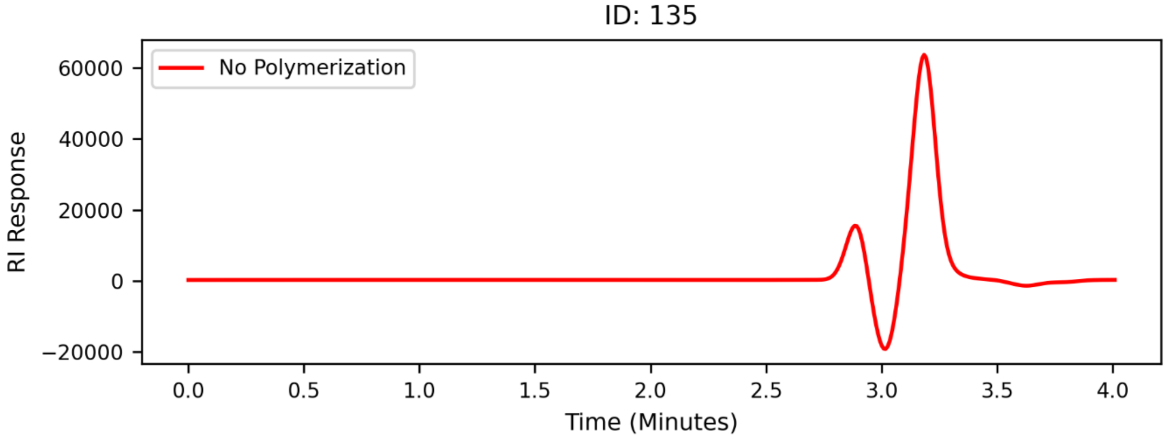
ID: 135
No Polymerization
RI Response
60000
40000
20000
0
−20000
0.0
0.5
1.0
1.5
2.0
2.5
3.0
3.5
4.0
Time (Minutes)

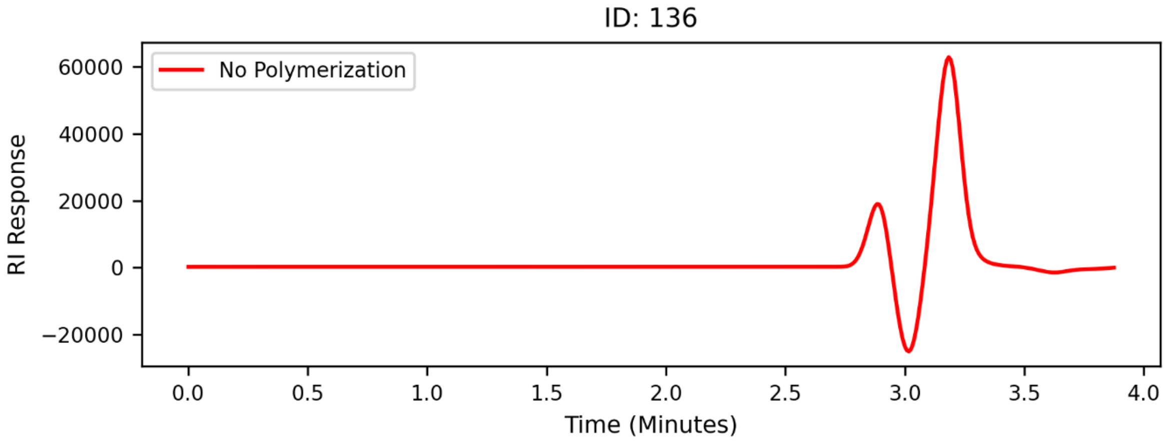
ID: 136
No Polymerization
RI Response
60000
40000
20000
0
−20000
0.0
0.5
1.0
1.5
2.0
2.5
3.0
3.5
4.0
Time (Minutes)

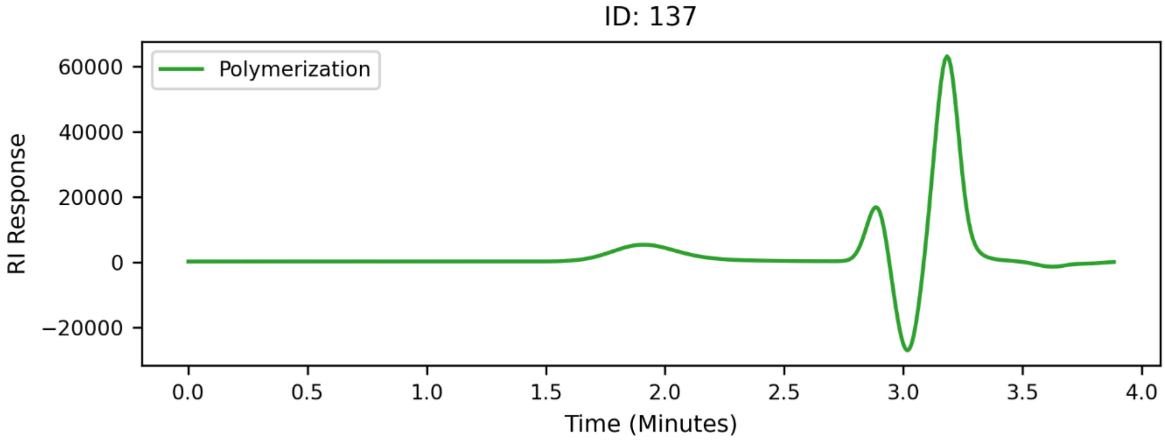
ID: 137
Polymerization
RI Response
60000
40000
20000
0
−20000
0.0
0.5
1.0
1.5
2.0
2.5
3.0
3.5
4.0
Time (Minutes)

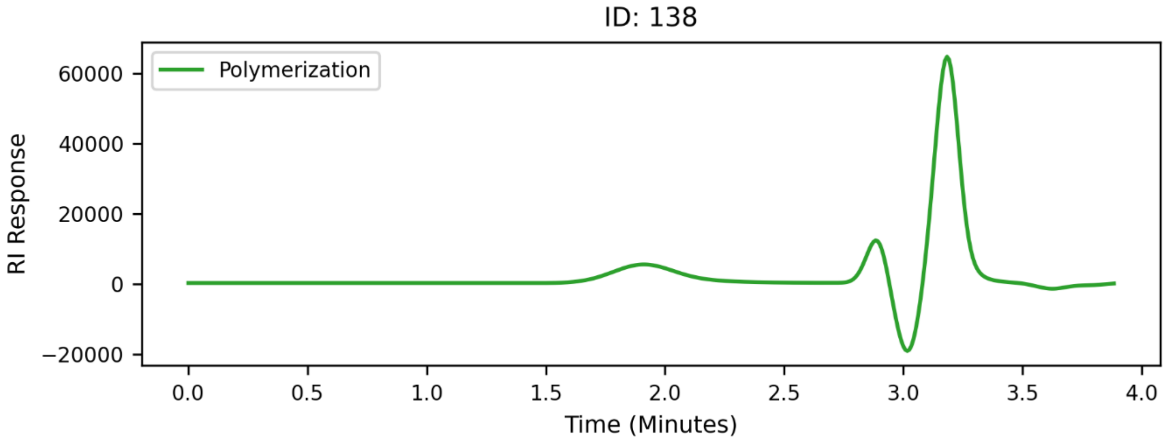
ID: 138
Polymerization
RI Response
60000
40000
20000
0
−20000
0.0
0.5
1.0
1.5
2.0
2.5
3.0
3.5
4.0
Time (Minutes)

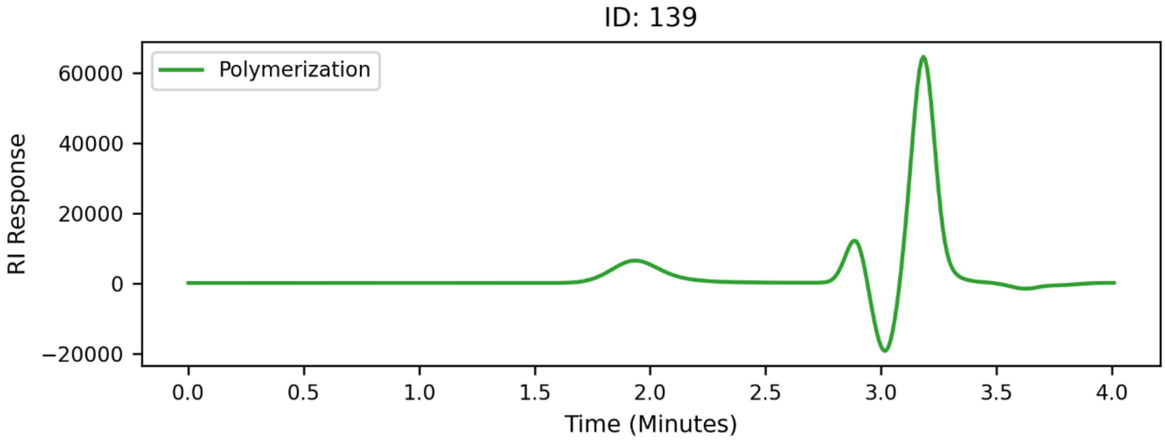
ID: 139
Polymerization
RI Response
60000
40000
20000
0
−20000
0.0
0.5
1.0
1.5
2.0
2.5
3.0
3.5
4.0
Time (Minutes)

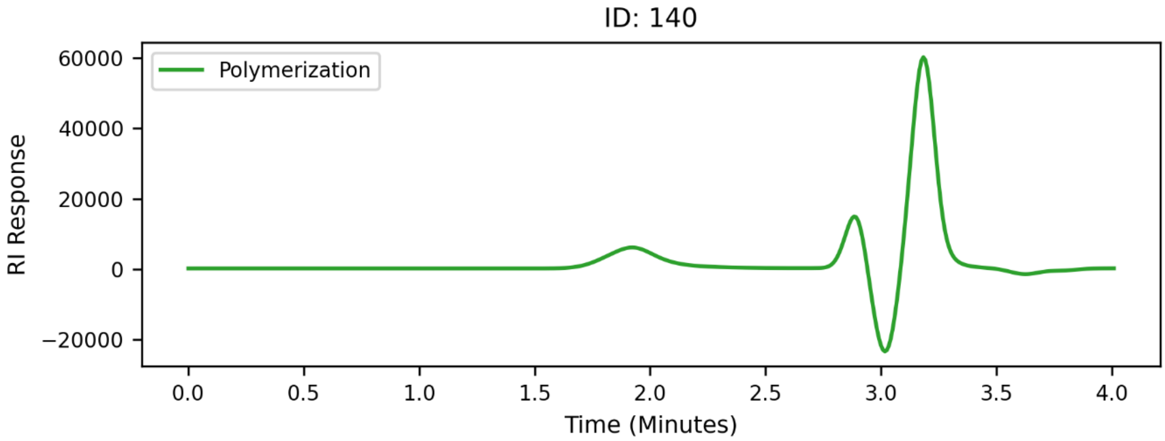
ID: 140
Polymerization
RI Response
60000
40000
20000
0
−20000
0.0
0.5
1.0
1.5
2.0
2.5
3.0
3.5
4.0
Time (Minutes)

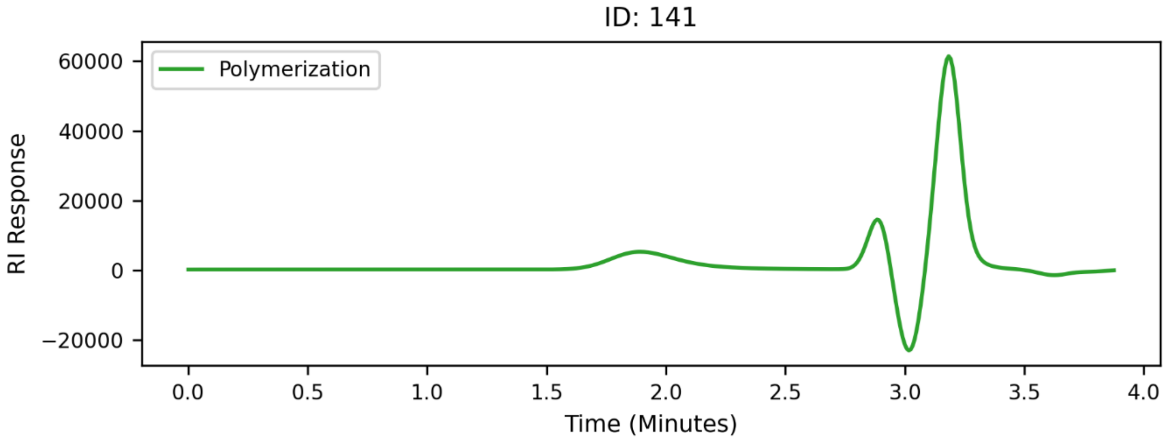
ID: 141
Polymerization
RI Response
60000
40000
20000
0
−20000
0.0
0.5
1.0
1.5
2.0
2.5
3.0
3.5
4.0
Time (Minutes)

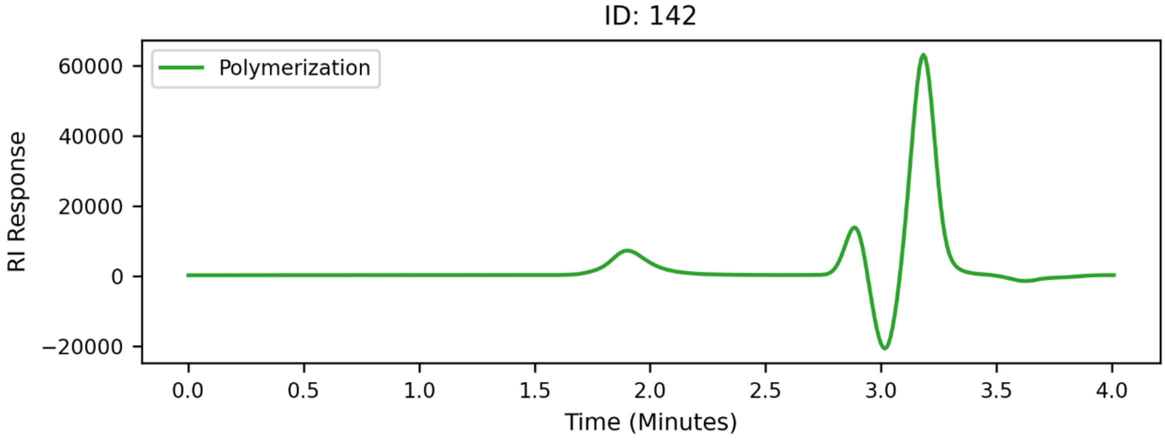
ID: 142
Polymerization
RI Response
60000
40000
20000
0
−20000
0.0
0.5
1.0
1.5
2.0
2.5
3.0
3.5
4.0
Time (Minutes)

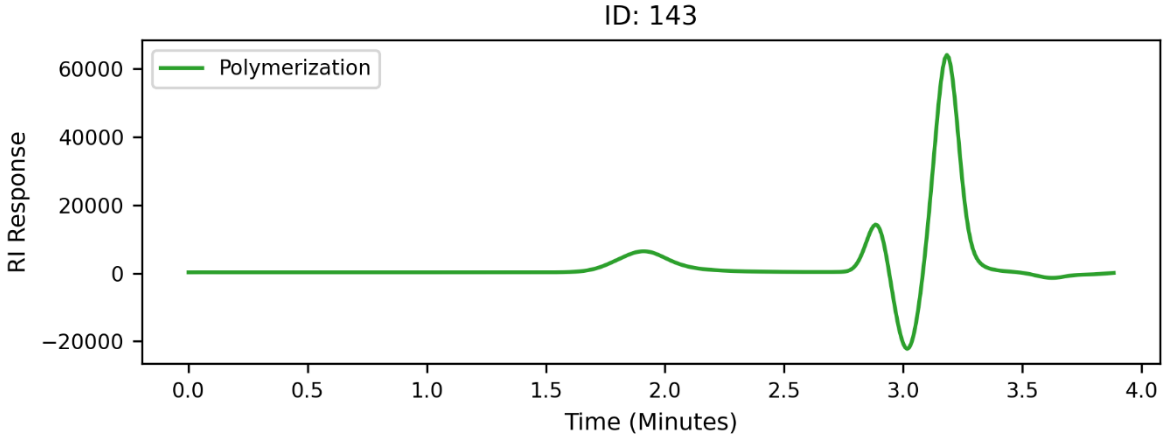
ID: 143
Polymerization
RI Response
60000
40000
20000
0
−20000
0.0
0.5
1.0
1.5
2.0
2.5
3.0
3.5
4.0
Time (Minutes)

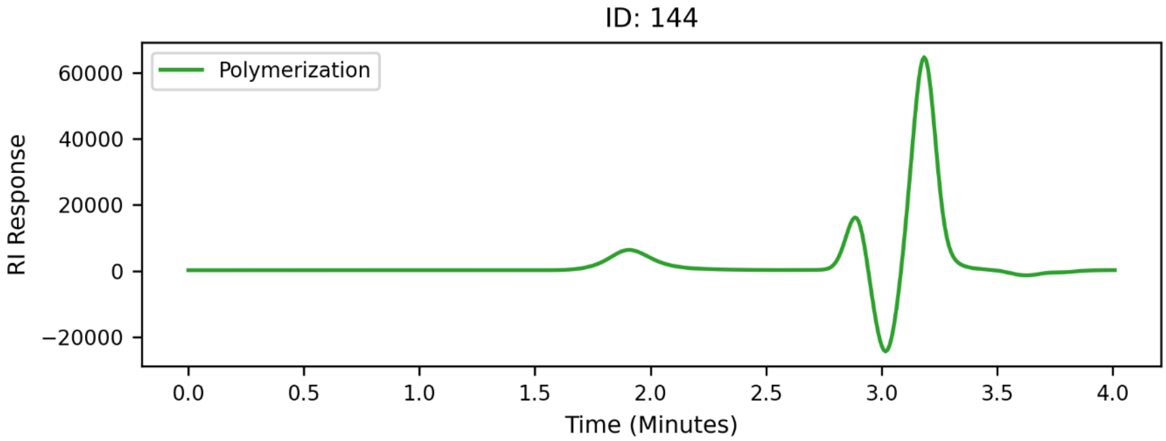
ID: 144
Polymerization
60000
40000
20000
0
−20000
RI Response
0.0
0.5
1.0
1.5
2.0
2.5
3.0
3.5
4.0
Time (Minutes)

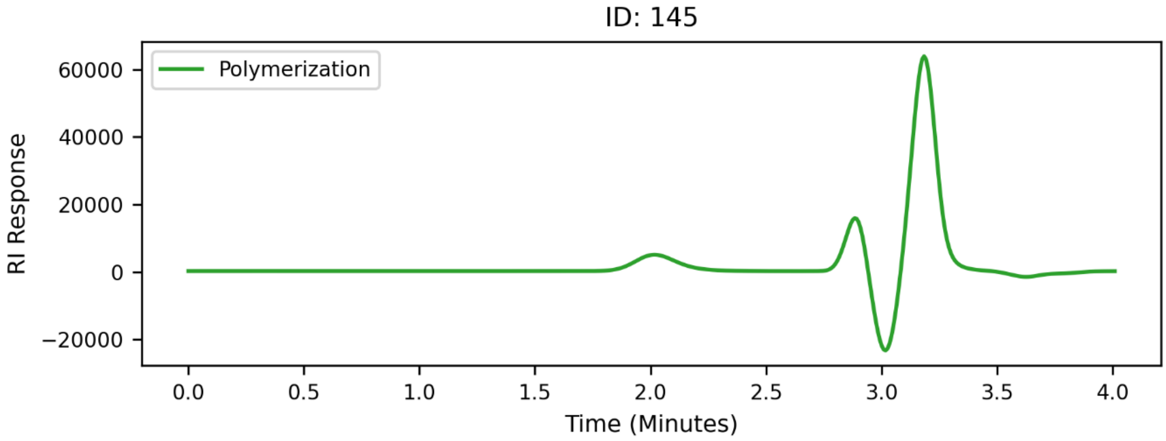
ID: 145
Polymerization
60000
40000
20000
0
−20000
RI Response
0.0
0.5
1.0
1.5
2.0
2.5
3.0
3.5
4.0
Time (Minutes)

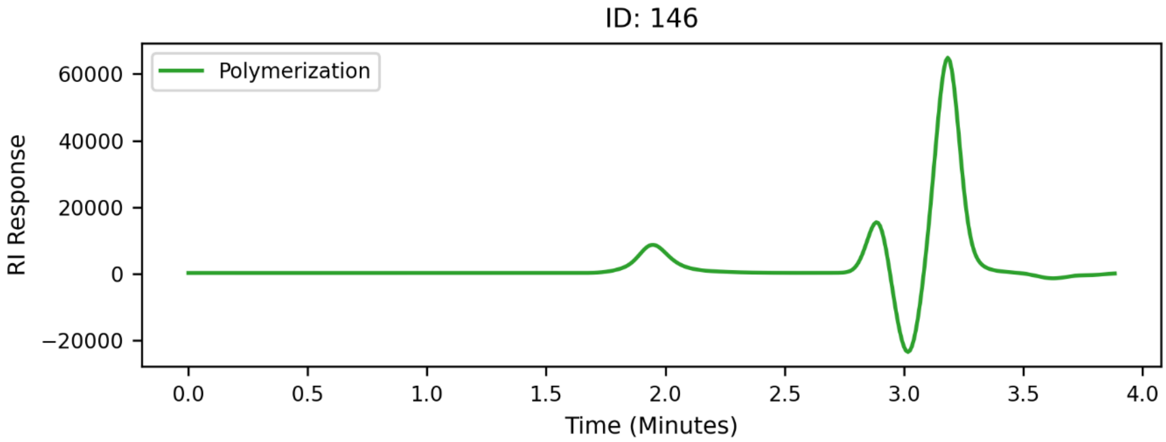
ID: 146
Polymerization
60000
40000
20000
0
−20000
RI Response
0.0
0.5
1.0
1.5
2.0
2.5
3.0
3.5
4.0
Time (Minutes)

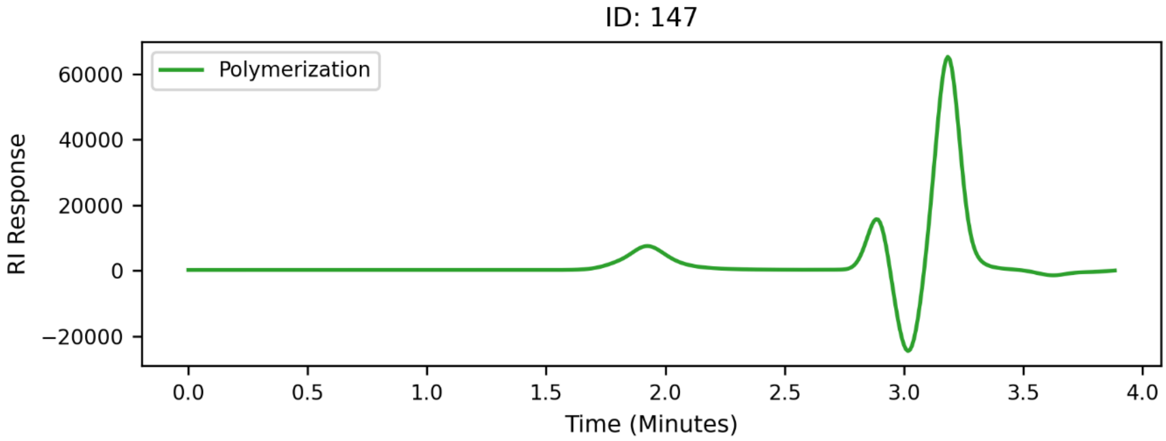
ID: 147
Polymerization
60000
40000
20000
0
−20000
RI Response
0.0
0.5
1.0
1.5
2.0
2.5
3.0
3.5
4.0
Time (Minutes)

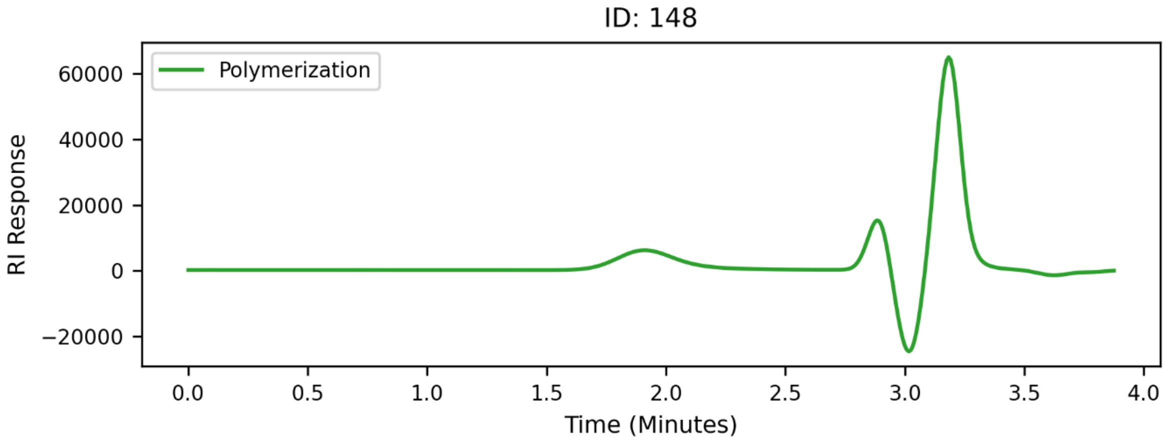
ID: 148
Polymerization
60000
40000
20000
0
−20000
RI Response
0.0
0.5
1.0
1.5
2.0
2.5
3.0
3.5
4.0
Time (Minutes)

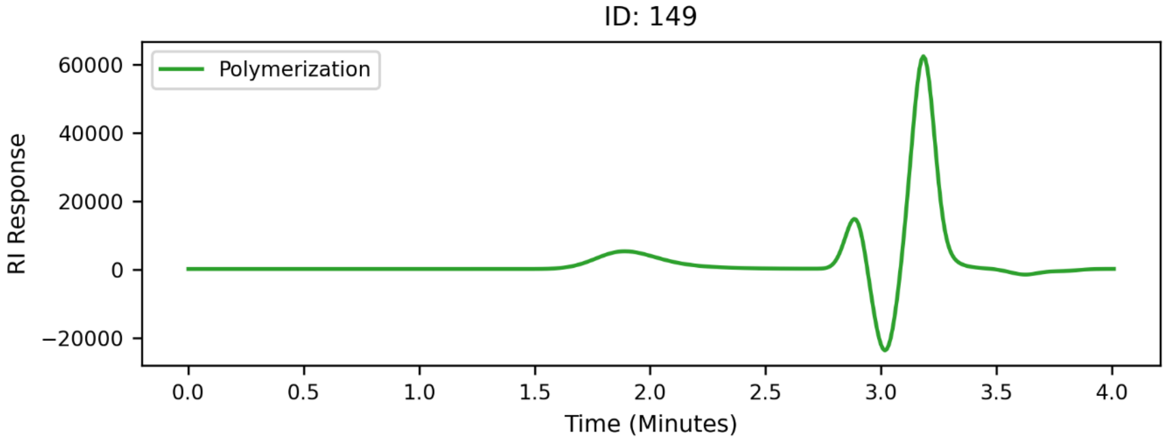
ID: 149
Polymerization
60000
40000
20000
0
−20000
RI Response
0.0
0.5
1.0
1.5
2.0
2.5
3.0
3.5
4.0
Time (Minutes)

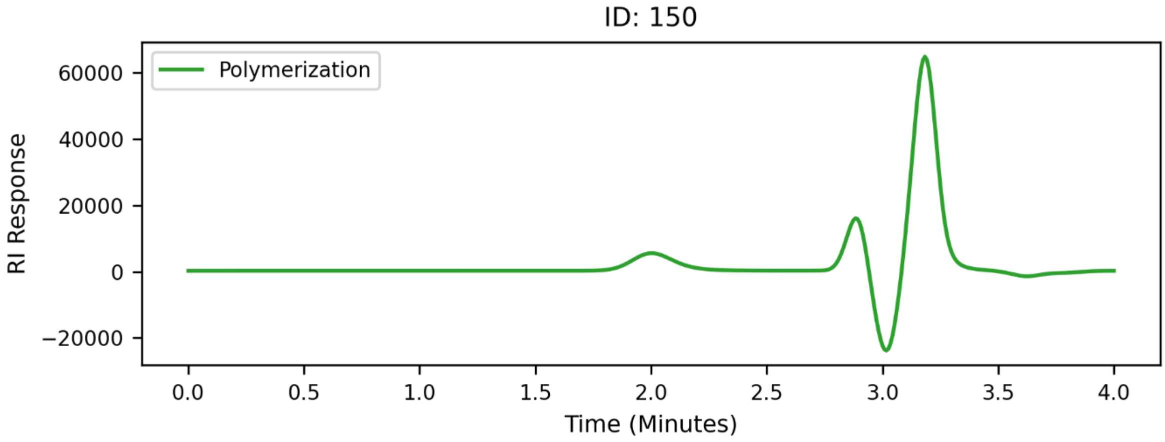
ID: 150
Polymerization
60000
40000
20000
0
−20000
RI Response
0.0
0.5
1.0
1.5
2.0
2.5
3.0
3.5
4.0
Time (Minutes)

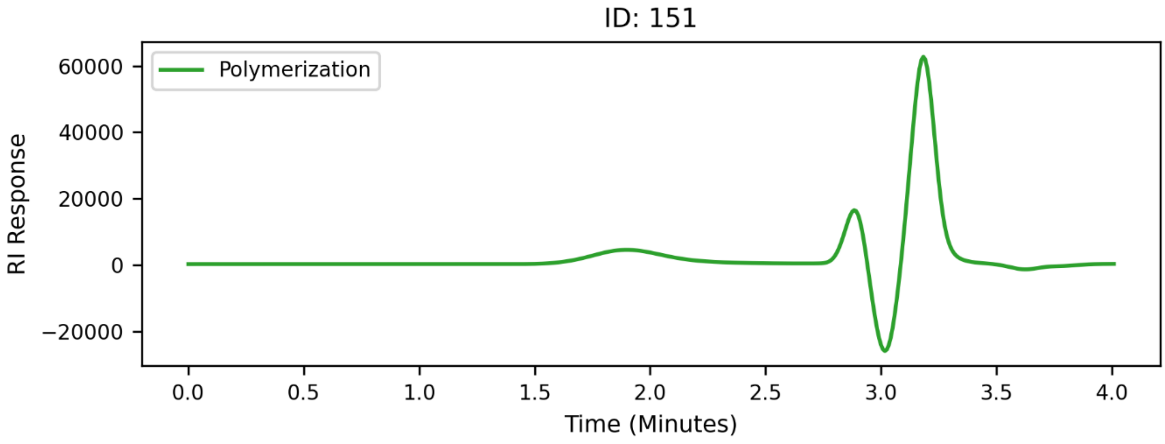
ID: 151
Polymerization
60000
40000
20000
0
−20000
RI Response
0.0
0.5
1.0
1.5
2.0
2.5
3.0
3.5
4.0
Time (Minutes)

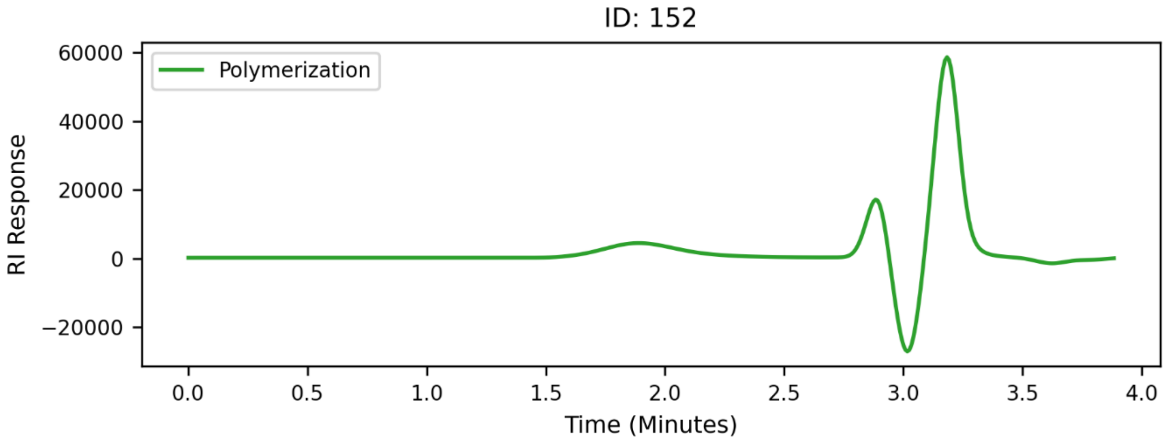
ID: 152
Polymerization
60000
40000
20000
0
−20000
RI Response
0.0
0.5
1.0
1.5
2.0
2.5
3.0
3.5
4.0
Time (Minutes)

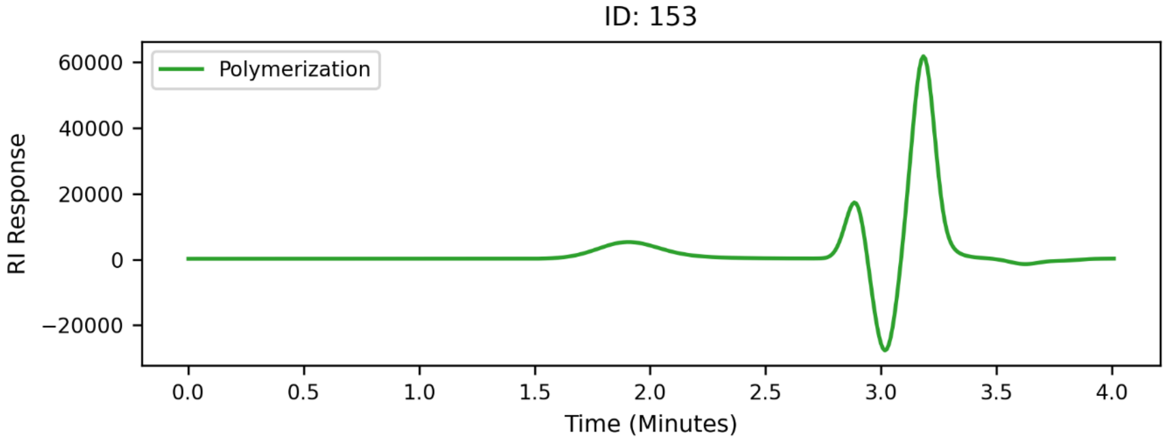
ID: 153
Polymerization
60000
40000
20000
0
−20000
RI Response
0.0
0.5
1.0
1.5
2.0
2.5
3.0
3.5
4.0
Time (Minutes)

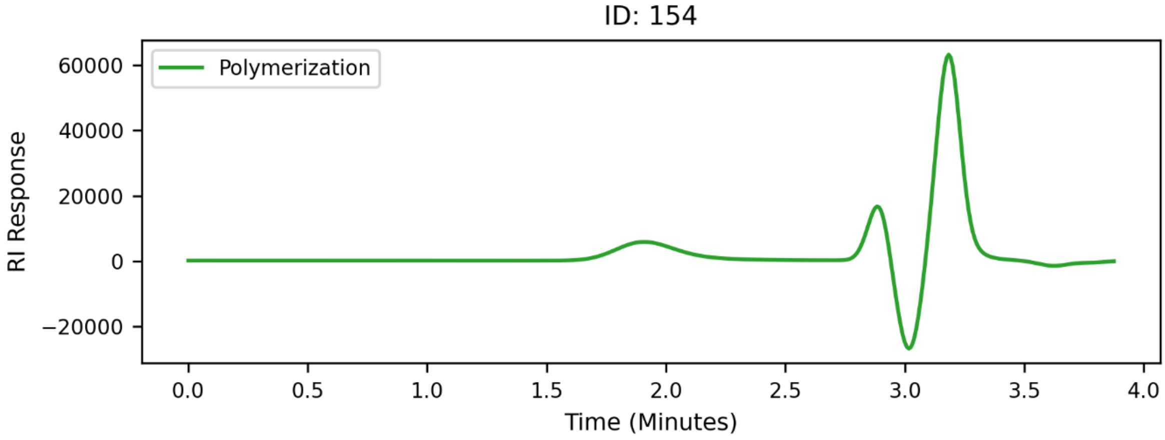
ID: 154
Polymerization
60000
40000
20000
0
−20000
RI Response
0.0
0.5
1.0
1.5
2.0
2.5
3.0
3.5
4.0
Time (Minutes)

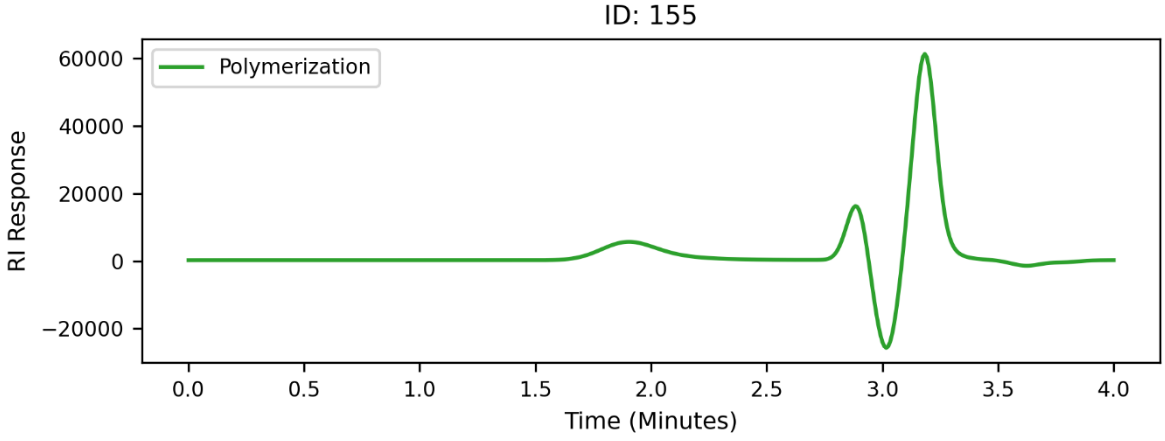
ID: 155
Polymerization
60000
40000
20000
0
−20000
RI Response
0.0
0.5
1.0
1.5
2.0
2.5
3.0
3.5
4.0
Time (Minutes)

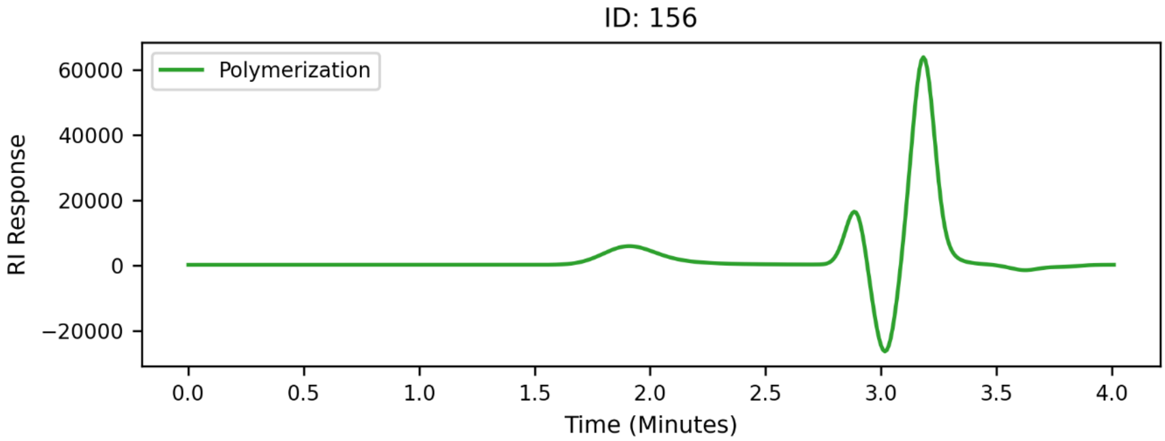
ID: 156
Polymerization
RI Response
60000
40000
20000
0
−20000
0.0
0.5
1.0
1.5
2.0
2.5
3.0
3.5
4.0
Time (Minutes)

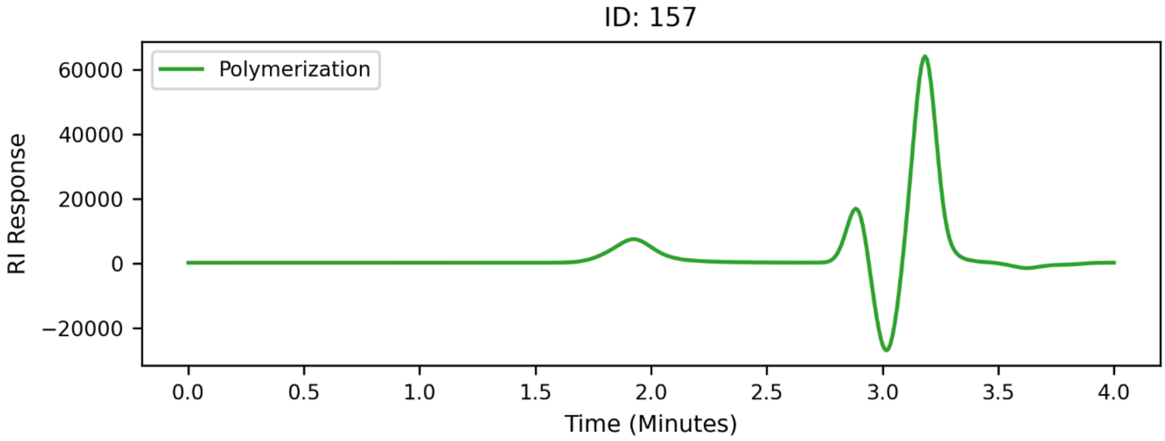
ID: 157
Polymerization
RI Response
60000
40000
20000
0
−20000
0.0
0.5
1.0
1.5
2.0
2.5
3.0
3.5
4.0
Time (Minutes)

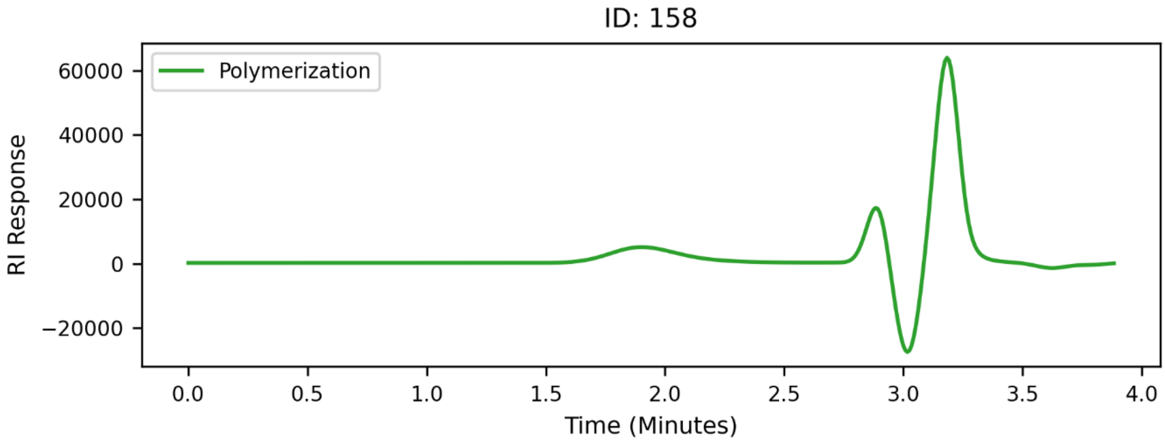
ID: 158
Polymerization
RI Response
60000
40000
20000
0
−20000
0.0
0.5
1.0
1.5
2.0
2.5
3.0
3.5
4.0
Time (Minutes)

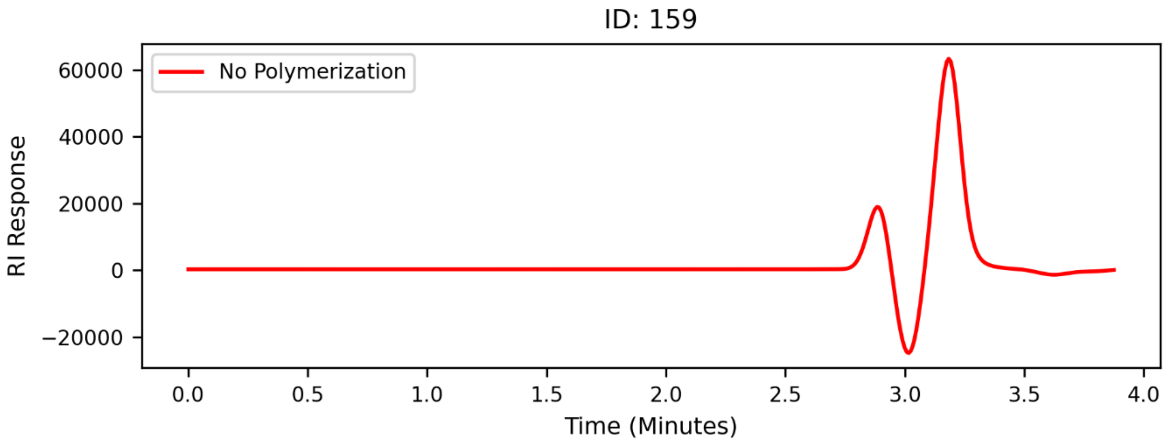
ID: 159
No Polymerization
RI Response
60000
40000
20000
0
−20000
0.0
0.5
1.0
1.5
2.0
2.5
3.0
3.5
4.0
Time (Minutes)

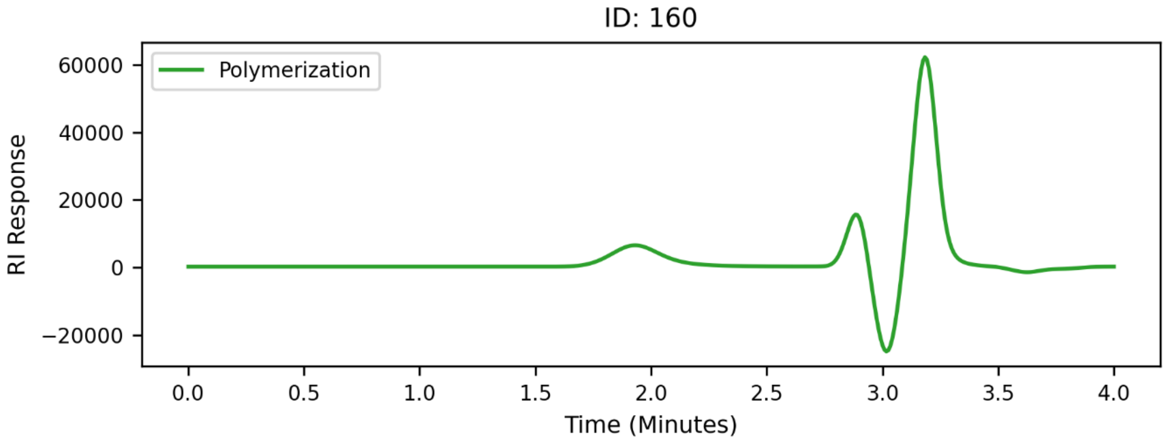
ID: 160
Polymerization
RI Response
60000
40000
20000
0
−20000
0.0
0.5
1.0
1.5
2.0
2.5
3.0
3.5
4.0
Time (Minutes)

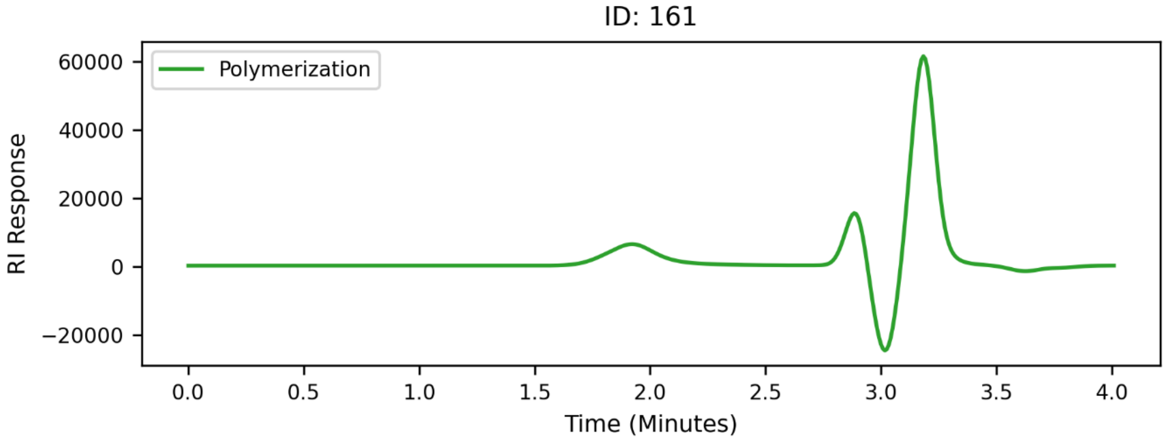
ID: 161
Polymerization
RI Response
60000
40000
20000
0
−20000
0.0
0.5
1.0
1.5
2.0
2.5
3.0
3.5
4.0
Time (Minutes)

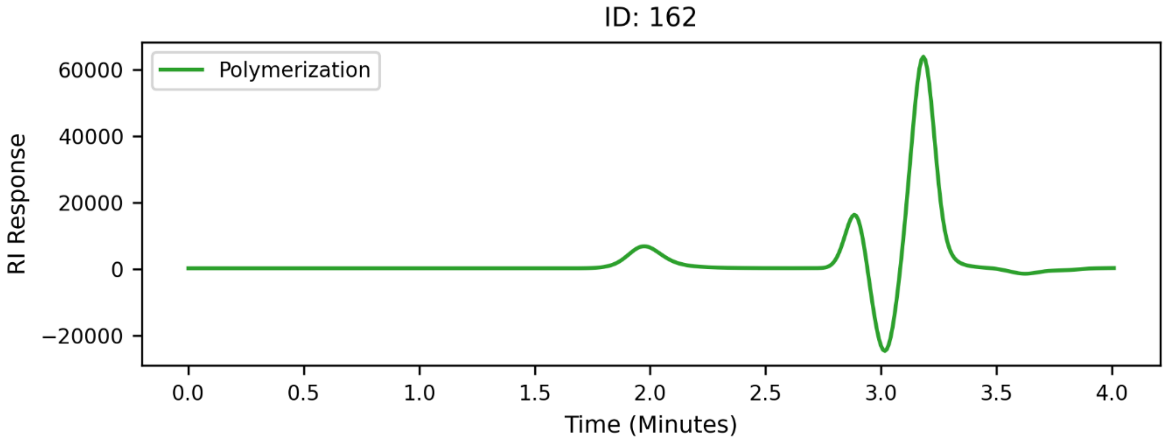
ID: 162
Polymerization
RI Response
60000
40000
20000
0
−20000
0.0
0.5
1.0
1.5
2.0
2.5
3.0
3.5
4.0
Time (Minutes)

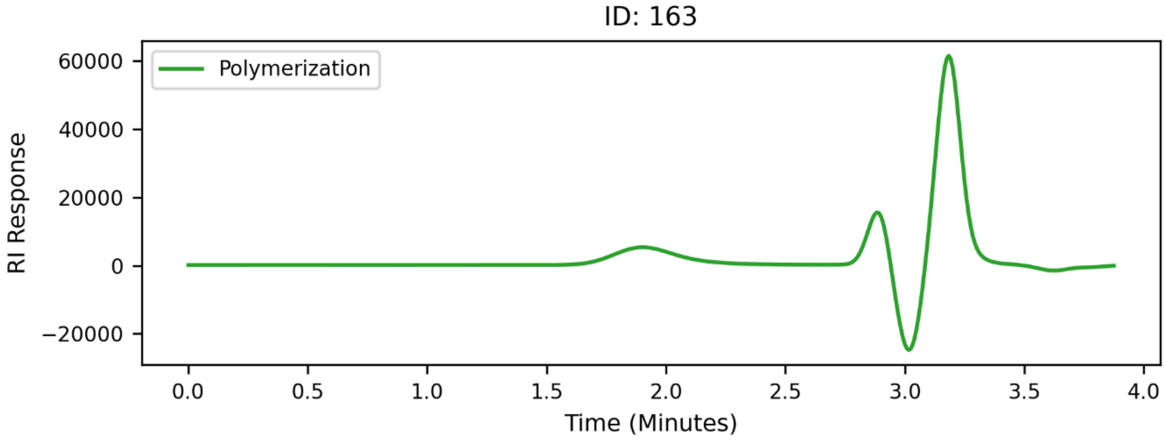
ID: 163
Polymerization
RI Response
60000
40000
20000
0
−20000
0.0
0.5
1.0
1.5
2.0
2.5
3.0
3.5
4.0
Time (Minutes)

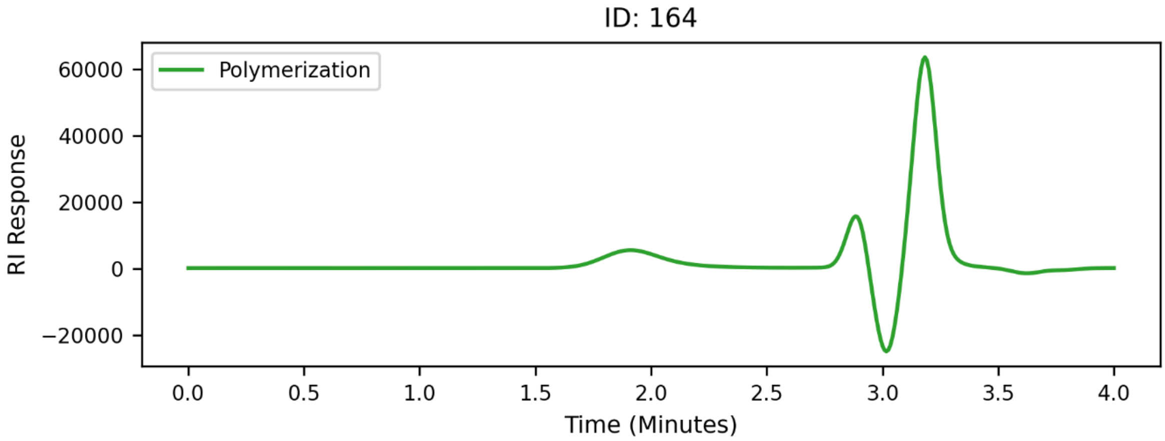
ID: 164
Polymerization
RI Response
60000
40000
20000
0
−20000
0.0
0.5
1.0
1.5
2.0
2.5
3.0
3.5
4.0
Time (Minutes)

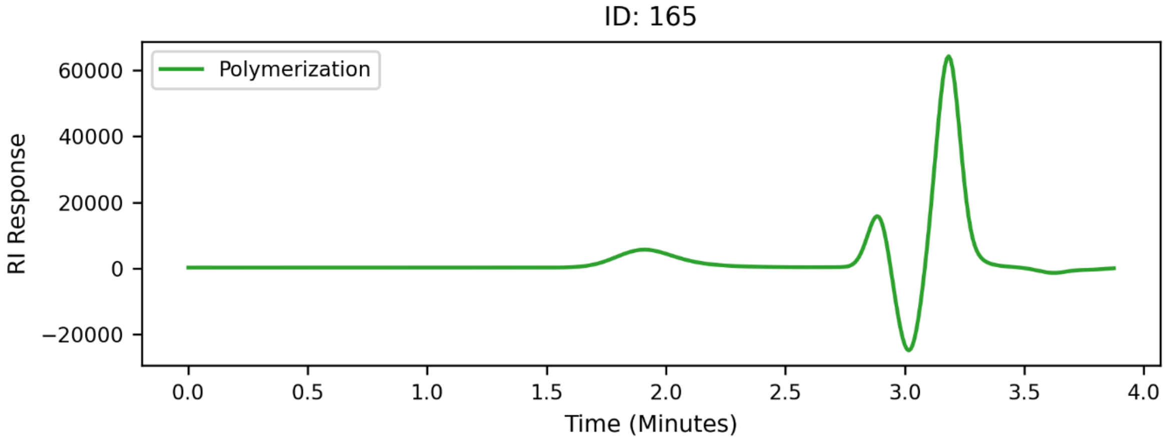
ID: 165
Polymerization
RI Response
60000
40000
20000
0
−20000
0.0
0.5
1.0
1.5
2.0
2.5
3.0
3.5
4.0
Time (Minutes)

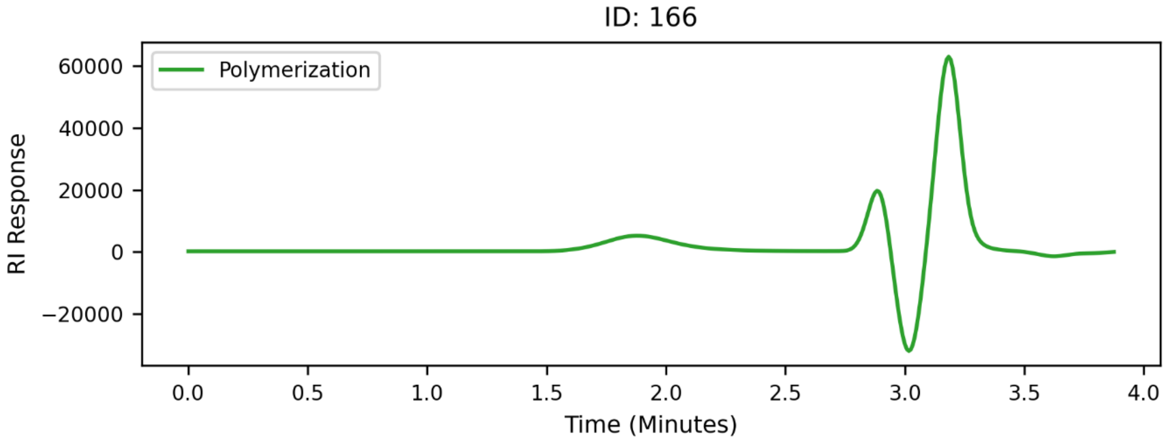
ID: 166
Polymerization
RI Response
60000
40000
20000
0
−20000
0.0
0.5
1.0
1.5
2.0
2.5
3.0
3.5
4.0
Time (Minutes)

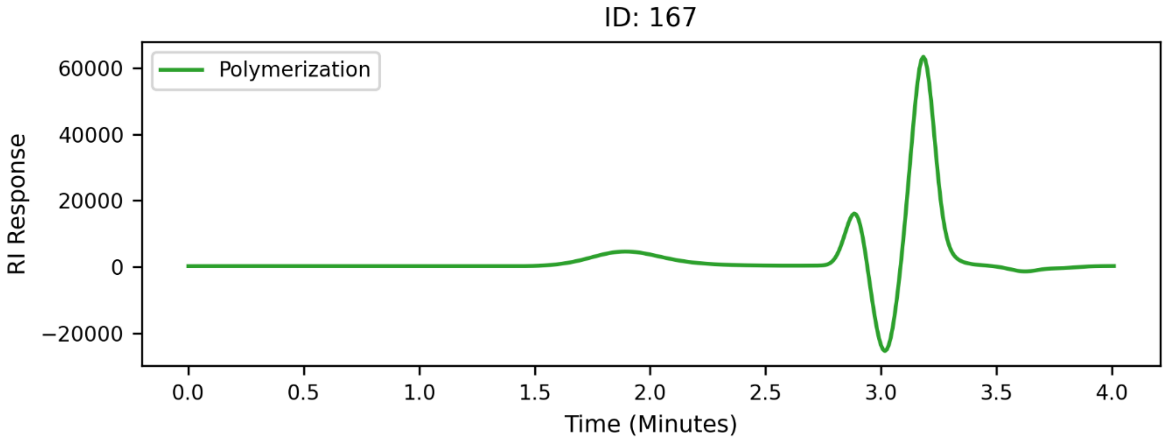
ID: 167
Polymerization
RI Response
60000
40000
20000
0
−20000
0.0
0.5
1.0
1.5
2.0
2.5
3.0
3.5
4.0
Time (Minutes)
